# Supplementary material for: SUMOylation of protein phosphatase 5 regulates phosphatase activity and substrate release
Source: EMBO Rep. 2024 Sep 20;25(11):4. doi: 10.1038/s44319-024-00250-2 (PMC11549447; doi:10.1038/s44319-024-00250-2)
Supplement: Supplementary file 1 — Appendix [file 44319_2024_250_MOESM1_ESM.pdf]

Table of Contents

Appendix Table S1. *Bona fide* PP5 substrates. Related to Fig. 5A…………....1

Appendix Table S2. Genomic prevalence of motifs. Related to Fig. 5………..2

Appendix Table S3. Table of Primers. Related to Reagents and Tools Table….2

Appendix Table S1. *Bona fide* PP5 substrates.

Protein Residue	-7/+7 sequence	Ref
FNIP1-S938	WDIPRNEssDsALGD	(Sager *et al.*, 2019)
FNIP1-S939	DIPRNEssDsALGDs	(Sager *et al.*, 2019)
FNIP1-S941	PRNEssDsALGDsEs	(Sager *et al.*, 2019)
FNIP1-S946	sDsALGDsEsEDTGH	(Sager *et al.*, 2019)
FNIP1-S948	sALGDsEsEDTGHDM	(Sager *et al.*, 2019)
FADD-S194	QNRsGAMsPMsWNsD	(Ahanin *et al.*, 2023)
Cdc37-S13	VWDHIEVsDDEDETH	(Vaughan *et al.*, 2008)
Hsp90α-T90	NkQDRtLtIVDTGIG	(Wang *et al*, 2009)
GR-S203	DLEFSSGsPGkEtNE	(Wang *et al.*, 2007)
GR-S211	PGkEtNEsPWRSDLL	(Wang *et al.*, 2007)
GR-S226	IDENCLLsPLAGEDD	(Wang *et al.*, 2007)
Raf1-S338	RPRGQRDssyyWEIE	(von Kriegsheim *et al*, 2006)
ER-S118	LHPPPQLsPFLQPHG	(Ikeda *et al*, 2004)
PPARγ-S112	AIkVEPAsPPYYSEK	(Hinds & Sanchez, 2008)
Tau-S515	sGDRsGyssPGsPGt	(Liu *et al*, 2002)
Tau-S516	GDRsGyssPGsPGtP	(Liu *et al.*, 2002)
Tau-S519	sGyssPGsPGtPGsR	(Liu *et al.*, 2002)
Tau-S713	GAEIVyKsPVVsGDt	(Liu *et al.*, 2002)
Tau-S721	PVVsGDtsPRHLsNV	(Liu *et al.*, 2002)
Tau-S739	GsIDMVDsPQLAtLA	(Liu *et al.*, 2002)
53BP1-S25	PCLIIEDsQPEsQVL	(Kang *et al*, 2009)
53BP1-S1778	FNkQYTEsQLRAGAG	(Kang *et al.*, 2009)
AMPK-T172	sDGEFLRtsCGsPNY	(Hu *et al*, 2018)
DNA-PK T2609	LTPMFVETQASQGTL	(Wechsler *et al*, 2004)
DNA-PK S2056	VQSYSYSSQDPRPAT	(Wechsler *et al.*, 2004)
p53-S18	PsVEPPLsQEtFsDL	(Amable *et al*, 2011)
Chk1-S345	LVQGIsFsQPtCPDH	(Amable *et al.*, 2011)
Raf1-S289	RsHsEsAsPsALsss	(Mazalouskas *et al*, 2014)
Raf1-S296	sPsALsssPNNLsPt	(Mazalouskas *et al.*, 2014)
Raf1-S301	sssPNNLsPtGWsQP	(Mazalouskas *et al.*, 2014)
Titin-S4010	VRIEEGKsLRFPLAL	(Krysiak *et al*, 2018)

Appendix Table S2. Genomic prevalence of motifs

Gene Motif	Number of Genes	Ref
S-P	8,094	RCSB.org
E/D-S-P	7.994	
**Phosphorylation Motif**	**Number of Proteins**	**Ref**
pS-P	7,780	PhosphoSitePlus v6.6.0.4
E/D-pS-P	2,130	

Appendix Table S3. Table of Primers

Primer Name	Sequence
BamHI-FLAG-PP5-F	CTGTCGGATCCATGGATTACAAGGATGACGATGACAAGGGAGCGGAGGGCGAGAGGACTGA
PP5-ΔαJ-XhoI-R	ATCAGCTCGAGTCAATAGGCCATGGGCTT
PP5-K430R-F	ATCCGCAGCCACGAAGTCCGTGCCGAGGGCTACGAGGTG
PP5-K430R-R	CACCTCGTAGCCCTCGGCACGGACTTCGTGGCAGCGGAT
PP5-K430A-F	ATCCGCAGCCACGAAGTCGCTGCCGAGGGCTACGAGGTG
PP5-K430A-R	CACCTCGTAGCCCTCGGCAGCGACTTCGTGGCAGCGGAT
PP5-K430Q-F	ATCCGCAGCCACGAAGTCCAAGCCGAGGGCTACGAGGTG
PP5-K430Q-R	CACCTCGTAGCCCTCGGCTTGGACTTCGTGGCAGCGGAT
PP5-K97E/R101E-F	GACAAGAAGTACATCGAGGGTTATTACGAGCGGGCTGCCAGCAAC
PP5-K97E/R101E-R	GTTGCTGGCAGCCCGCTCGTAATAACCCTCGATGTACTTCTTGTC
GR-E210A-F	AAAGAGACCAACGCAAGCCCGTGGCGT
GR-E210A-R	ACGCCACGGGCTTGCGTTGGTCTCTTT
GR-P212A-F	ACCAACGAAAGCGCGTGGCGTAGCGAC
GR-P212A-R	GTCGCTACGCCACGCGCTTTCGTTGGT
