# Supplementary figures and images for: SUMOylation of protein phosphatase 5 regulates phosphatase activity and substrate release (part 1 of 2)
Source: EMBO Rep. 2024 Sep 20;25(11):4. doi: 10.1038/s44319-024-00250-2 (PMC11549447; doi:10.1038/s44319-024-00250-2)

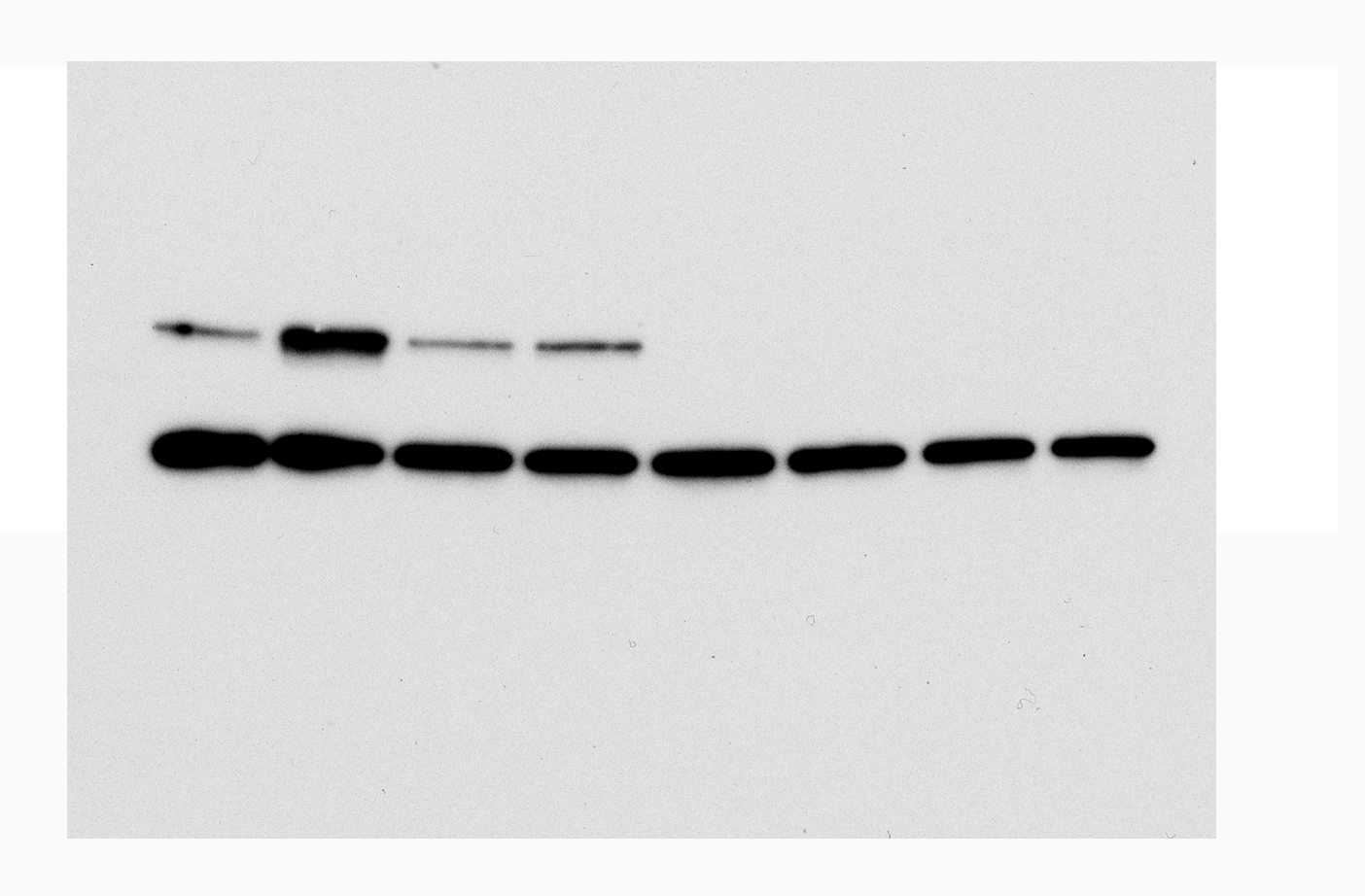

Supplement: Supplementary file 4 — Source data Fig. 1 [file 44319_2024_250_MOESM4_ESM.zip › EMBOR-2024-59387_SourceDataForFigure1/EMBOR-2024-59387_SourceDataForFigure1B/Western FLAG-IP.tif]

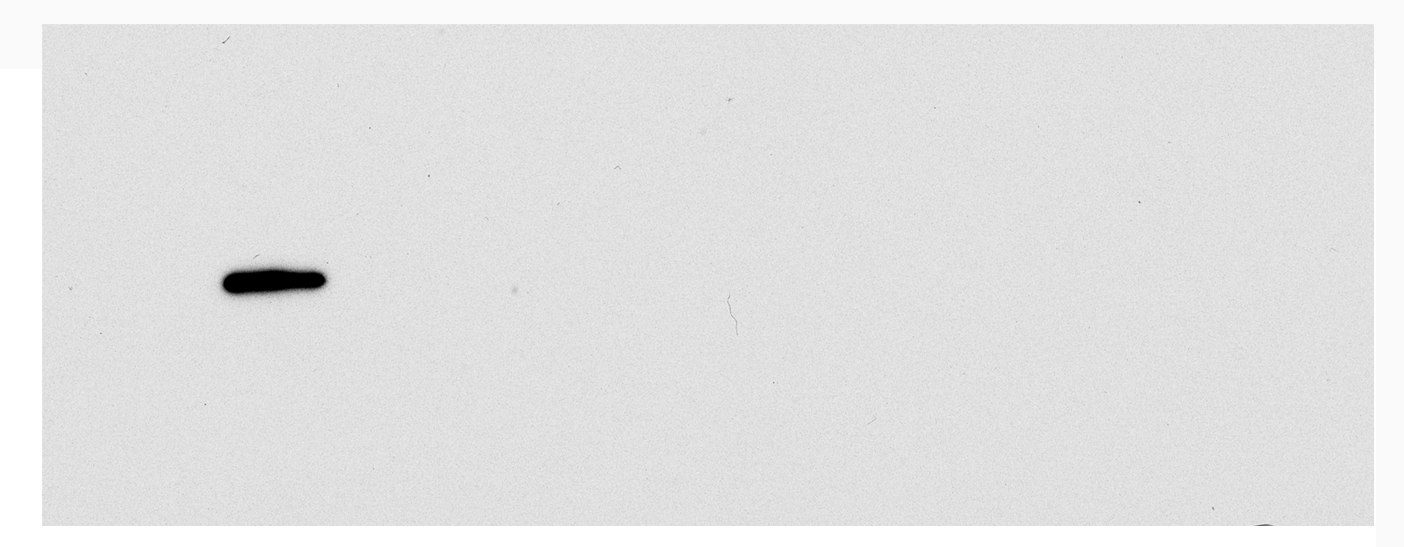

Supplement: Supplementary file 4 — Source data Fig. 1 [file 44319_2024_250_MOESM4_ESM.zip › EMBOR-2024-59387_SourceDataForFigure1/EMBOR-2024-59387_SourceDataForFigure1B/Western HA-tag-coIP.tif]

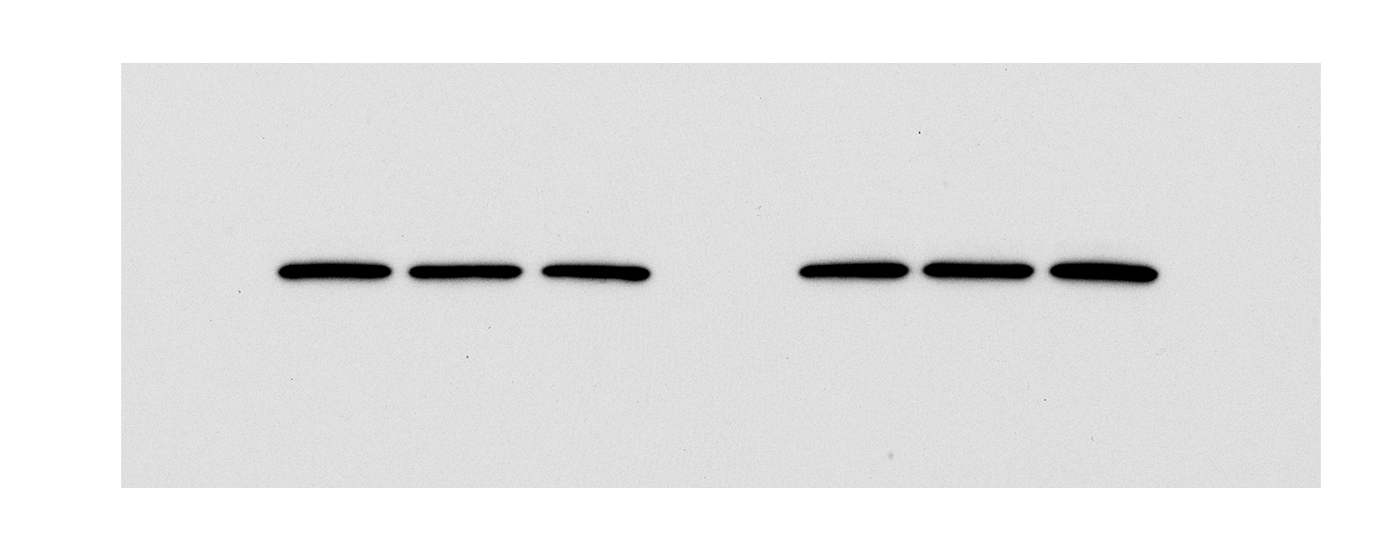

Supplement: Supplementary file 4 — Source data Fig. 1 [file 44319_2024_250_MOESM4_ESM.zip › EMBOR-2024-59387_SourceDataForFigure1/EMBOR-2024-59387_SourceDataForFigure1B/Western HA-tag-total.tif]

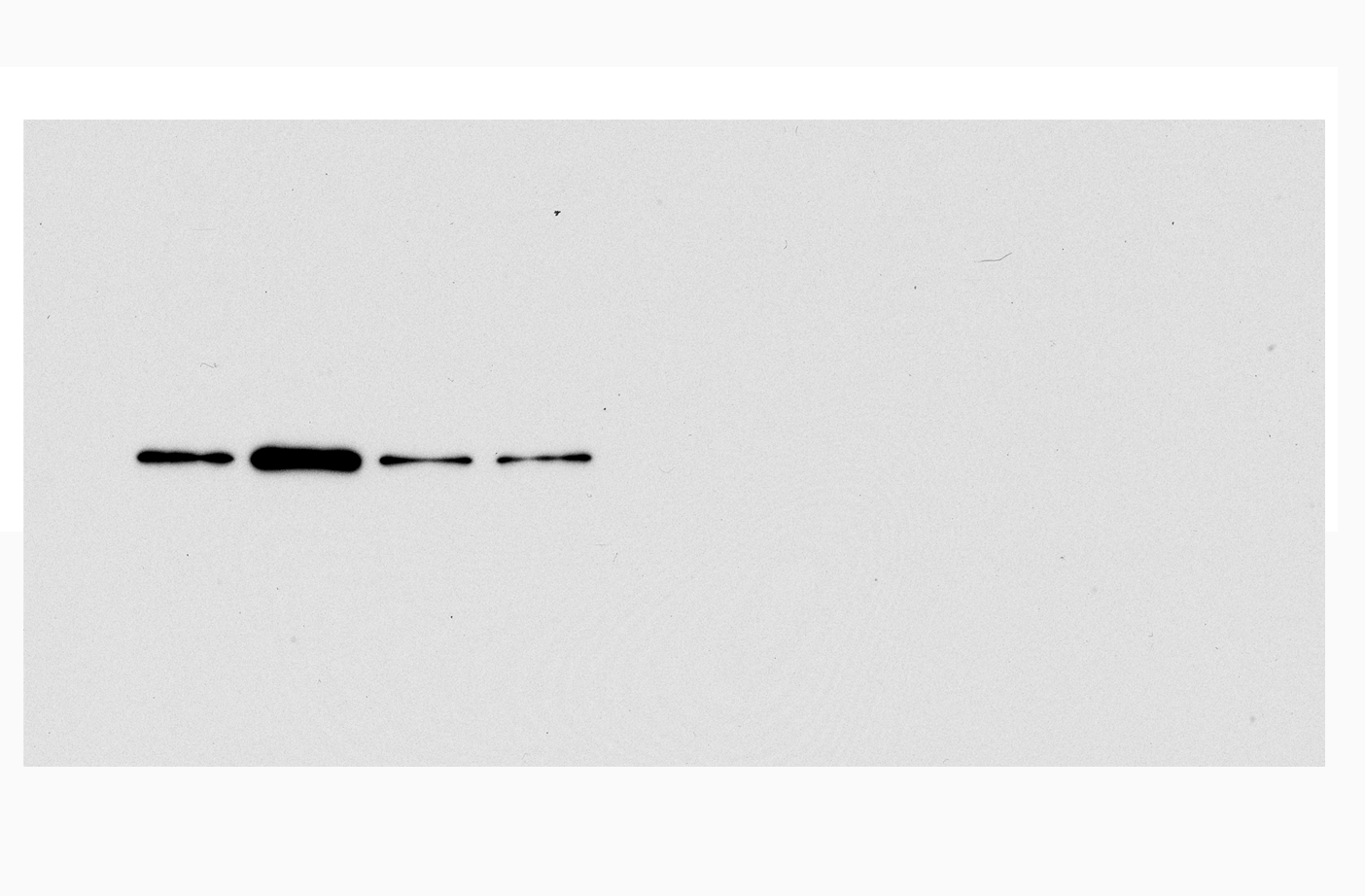

Supplement: Supplementary file 4 — Source data Fig. 1 [file 44319_2024_250_MOESM4_ESM.zip › EMBOR-2024-59387_SourceDataForFigure1/EMBOR-2024-59387_SourceDataForFigure1B/Western SUMO1-coIP.tif]

1B

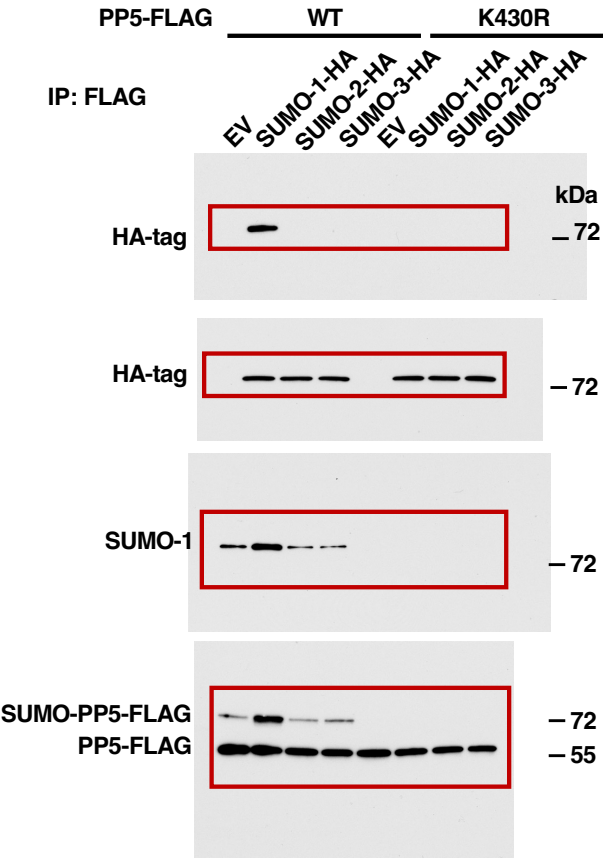

Supplement: Supplementary file 4 — Source data Fig. 1 [file 44319_2024_250_MOESM4_ESM.zip › EMBOR-2024-59387_SourceDataForFigure1/EMBOR-2024-59387_SourceDataForFigure1B/western uncropped annotated.pdf]

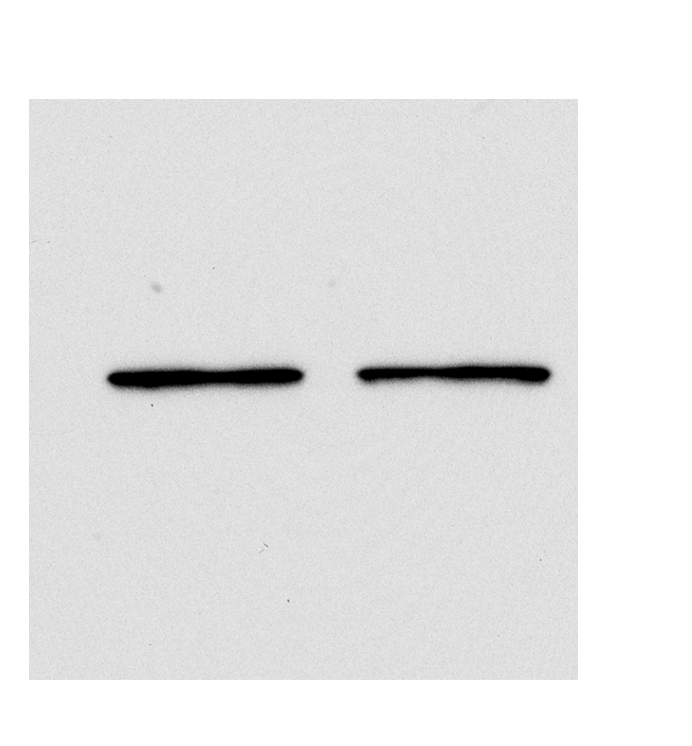

Supplement: Supplementary file 4 — Source data Fig. 1 [file 44319_2024_250_MOESM4_ESM.zip › EMBOR-2024-59387_SourceDataForFigure1/EMBOR-2024-59387_SourceDataForFigure1C/Western GAPDH-Total.tif]

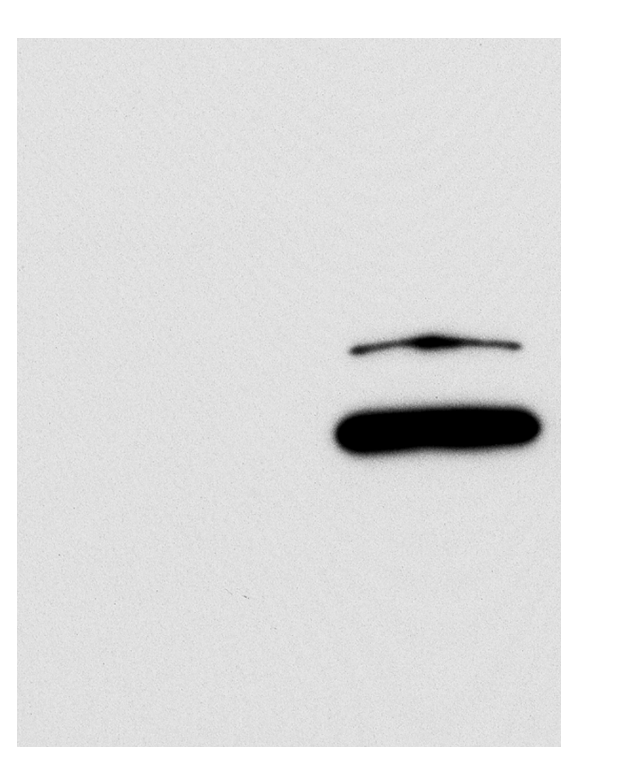

Supplement: Supplementary file 4 — Source data Fig. 1 [file 44319_2024_250_MOESM4_ESM.zip › EMBOR-2024-59387_SourceDataForFigure1/EMBOR-2024-59387_SourceDataForFigure1C/Western PP5-IP.tif]

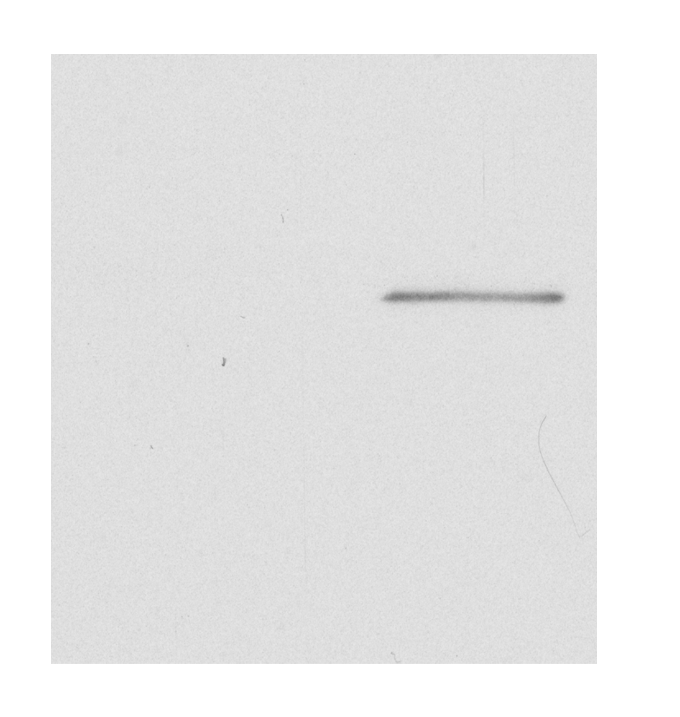

Supplement: Supplementary file 4 — Source data Fig. 1 [file 44319_2024_250_MOESM4_ESM.zip › EMBOR-2024-59387_SourceDataForFigure1/EMBOR-2024-59387_SourceDataForFigure1C/Western SUMO1-IP.tif]

1C

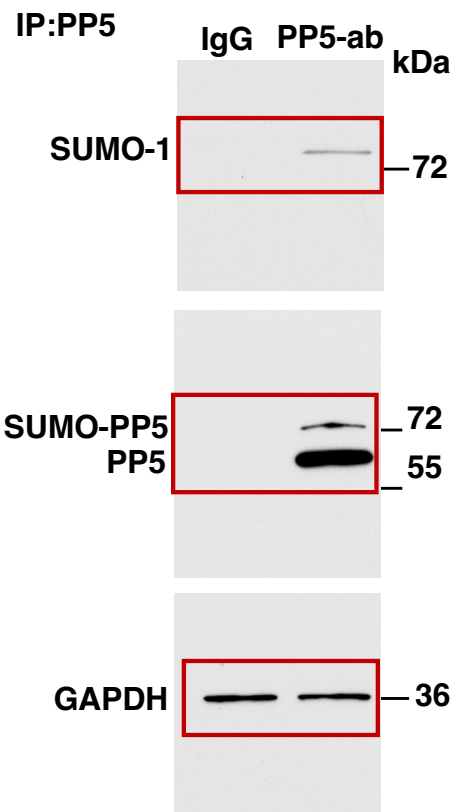

Supplement: Supplementary file 4 — Source data Fig. 1 [file 44319_2024_250_MOESM4_ESM.zip › EMBOR-2024-59387_SourceDataForFigure1/EMBOR-2024-59387_SourceDataForFigure1C/western uncropped annotated.pdf]

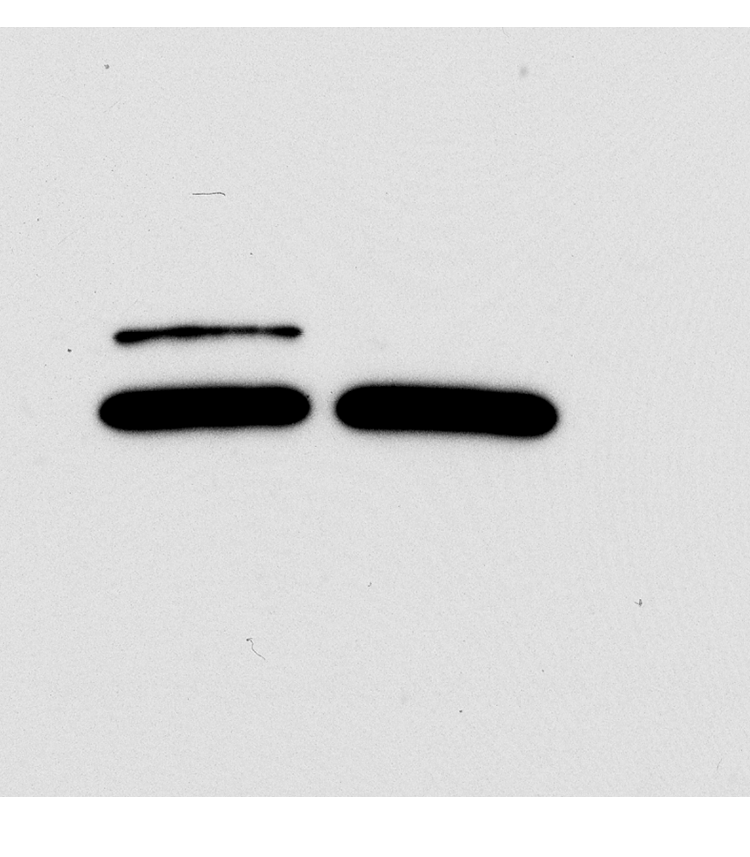

Supplement: Supplementary file 4 — Source data Fig. 1 [file 44319_2024_250_MOESM4_ESM.zip › EMBOR-2024-59387_SourceDataForFigure1/EMBOR-2024-59387_SourceDataForFigure1D/Western FLAG-IP.tif]

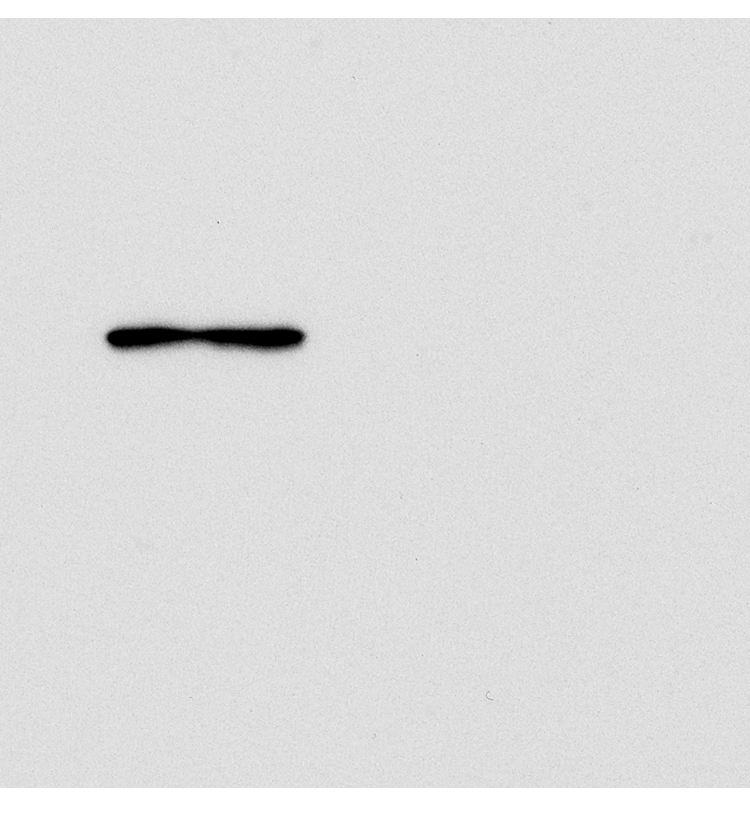

Supplement: Supplementary file 4 — Source data Fig. 1 [file 44319_2024_250_MOESM4_ESM.zip › EMBOR-2024-59387_SourceDataForFigure1/EMBOR-2024-59387_SourceDataForFigure1D/Western SUMO1-IP.tif]

1D

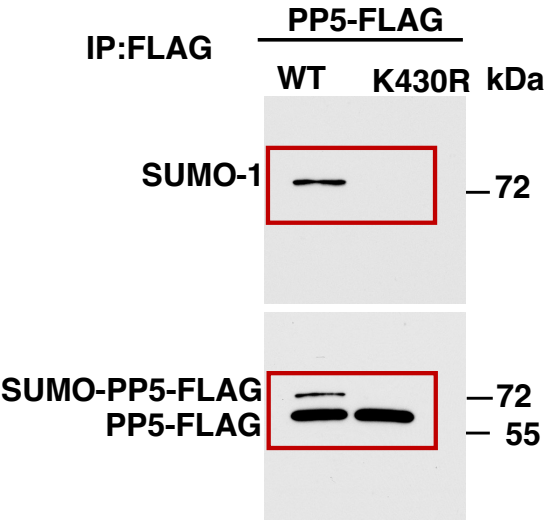

Supplement: Supplementary file 4 — Source data Fig. 1 [file 44319_2024_250_MOESM4_ESM.zip › EMBOR-2024-59387_SourceDataForFigure1/EMBOR-2024-59387_SourceDataForFigure1D/western uncropped annotated.pdf]

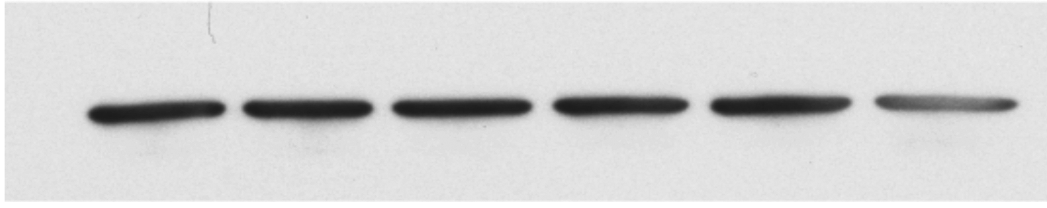

Supplement: Supplementary file 5 — Source data Fig. 2 [file 44319_2024_250_MOESM5_ESM.zip › EMBOR-2024-59387_SourceDataForFigure2/EMBOR-2024-59387_SourceDataForFigure2B/western GAPDH.tif]

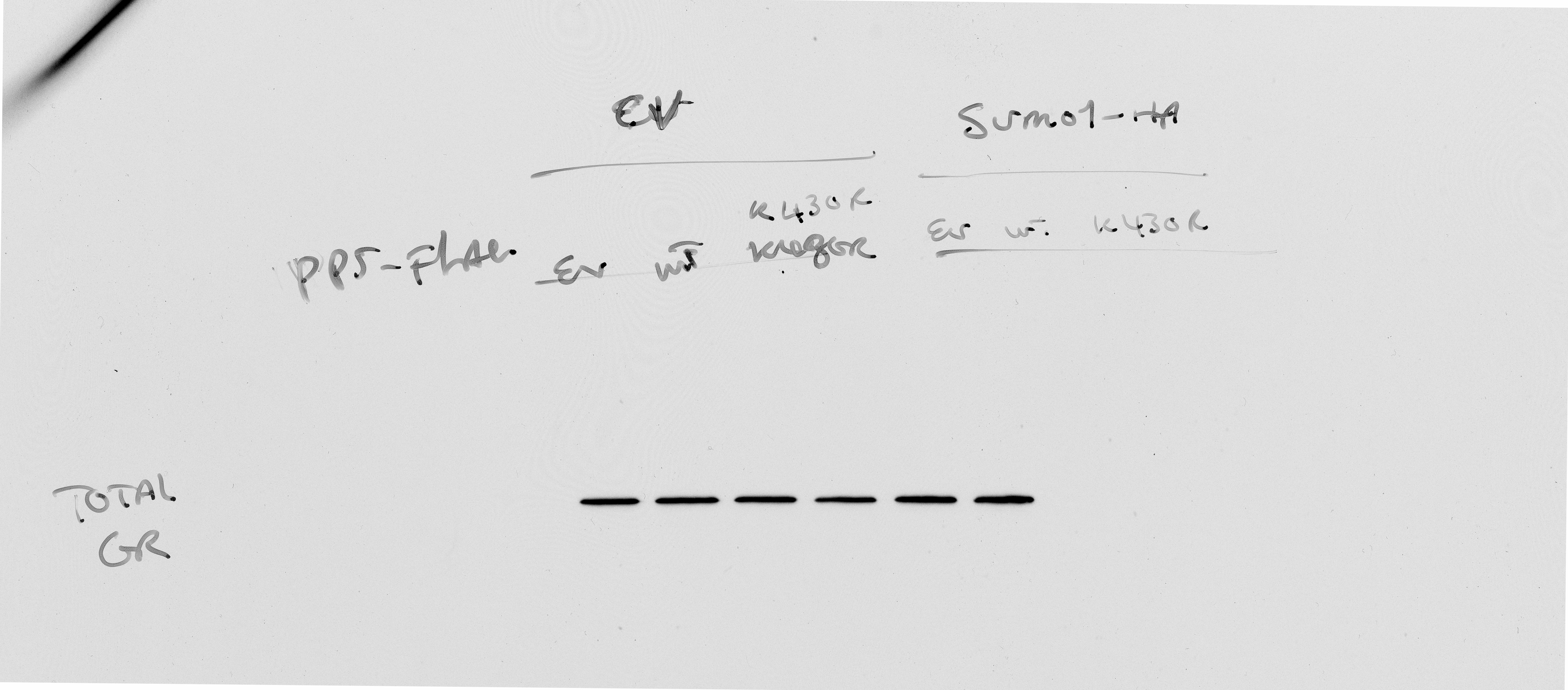

Supplement: Supplementary file 5 — Source data Fig. 2 [file 44319_2024_250_MOESM5_ESM.zip › EMBOR-2024-59387_SourceDataForFigure2/EMBOR-2024-59387_SourceDataForFigure2B/western GR.tif]

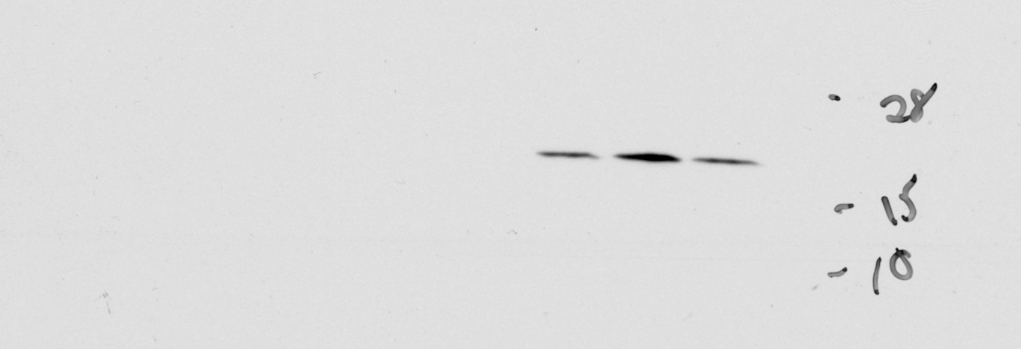

Supplement: Supplementary file 5 — Source data Fig. 2 [file 44319_2024_250_MOESM5_ESM.zip › EMBOR-2024-59387_SourceDataForFigure2/EMBOR-2024-59387_SourceDataForFigure2B/western HA-SUMO1.tif]

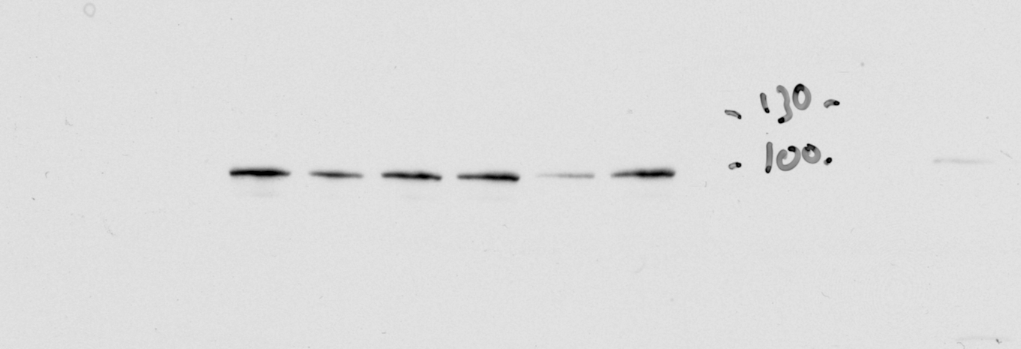

Supplement: Supplementary file 5 — Source data Fig. 2 [file 44319_2024_250_MOESM5_ESM.zip › EMBOR-2024-59387_SourceDataForFigure2/EMBOR-2024-59387_SourceDataForFigure2B/western phosGR-S211.tif]

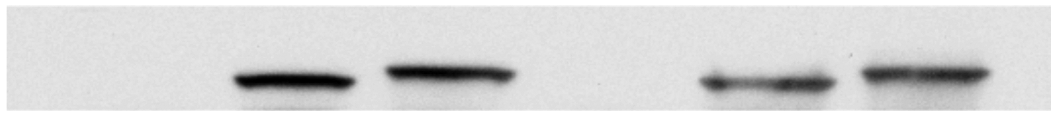

Supplement: Supplementary file 5 — Source data Fig. 2 [file 44319_2024_250_MOESM5_ESM.zip › EMBOR-2024-59387_SourceDataForFigure2/EMBOR-2024-59387_SourceDataForFigure2B/western PP5-FLAG.tif]

2B

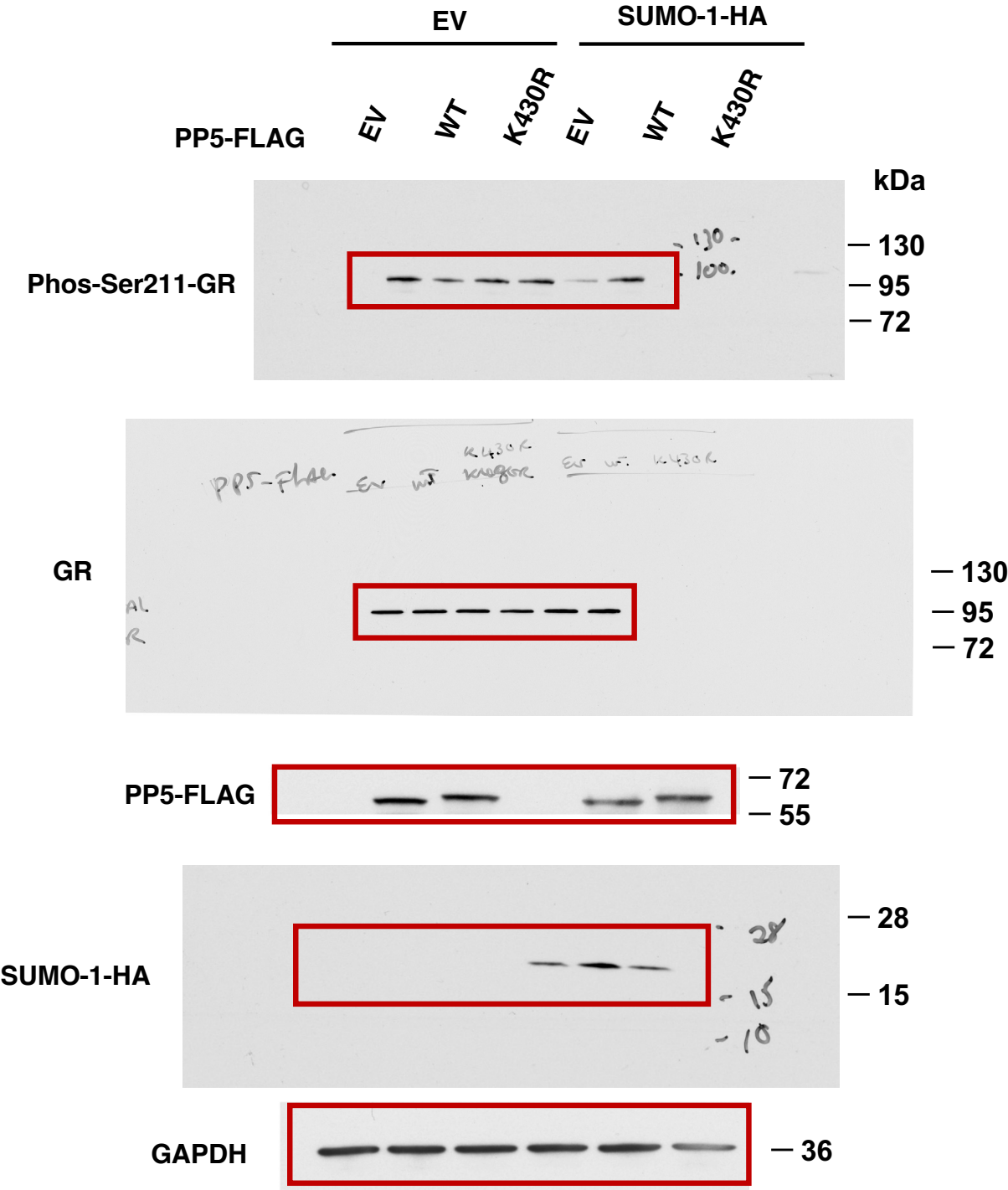

Supplement: Supplementary file 5 — Source data Fig. 2 [file 44319_2024_250_MOESM5_ESM.zip › EMBOR-2024-59387_SourceDataForFigure2/EMBOR-2024-59387_SourceDataForFigure2B/western uncropped annotated.pdf]

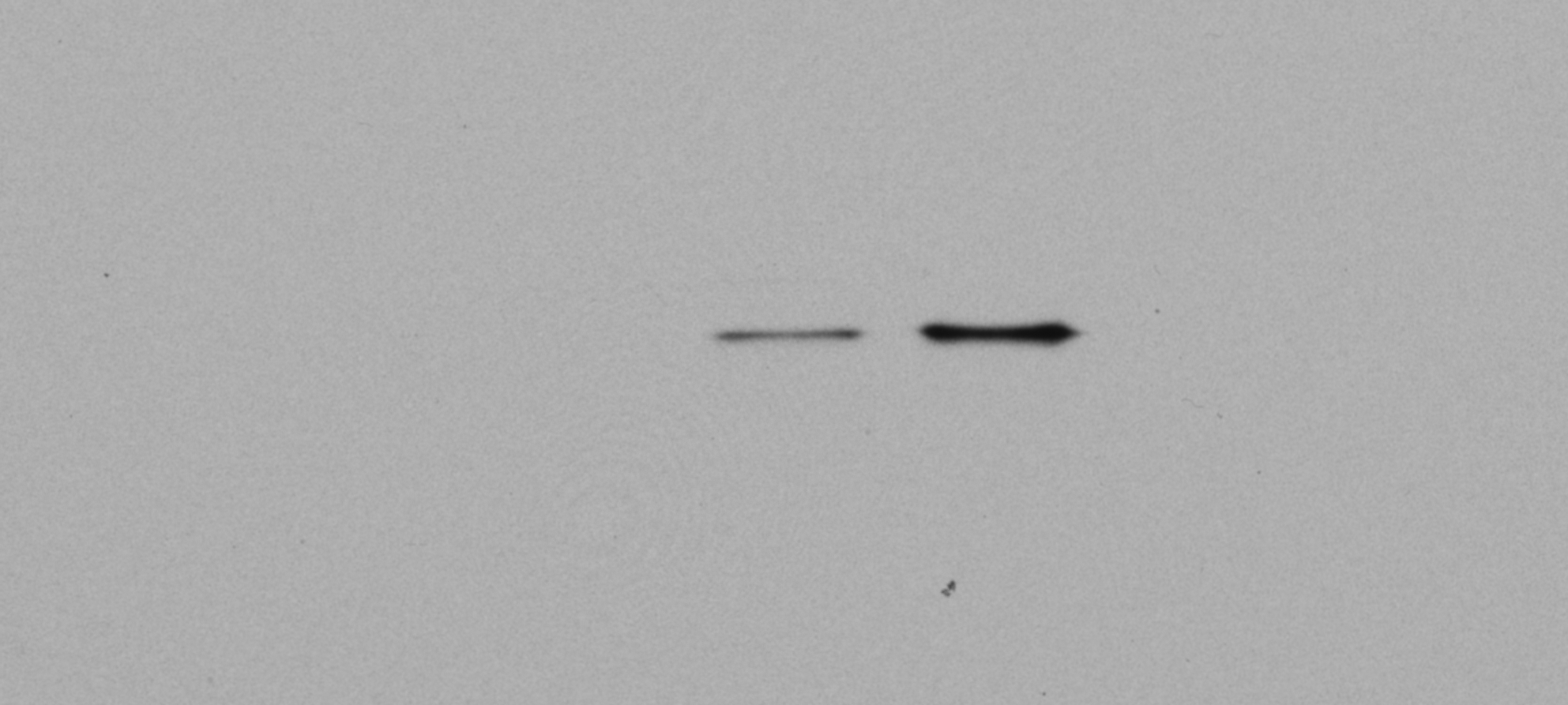

Supplement: Supplementary file 5 — Source data Fig. 2 [file 44319_2024_250_MOESM5_ESM.zip › EMBOR-2024-59387_SourceDataForFigure2/EMBOR-2024-59387_SourceDataForFigure2C/western Aha1 coIP.tif]

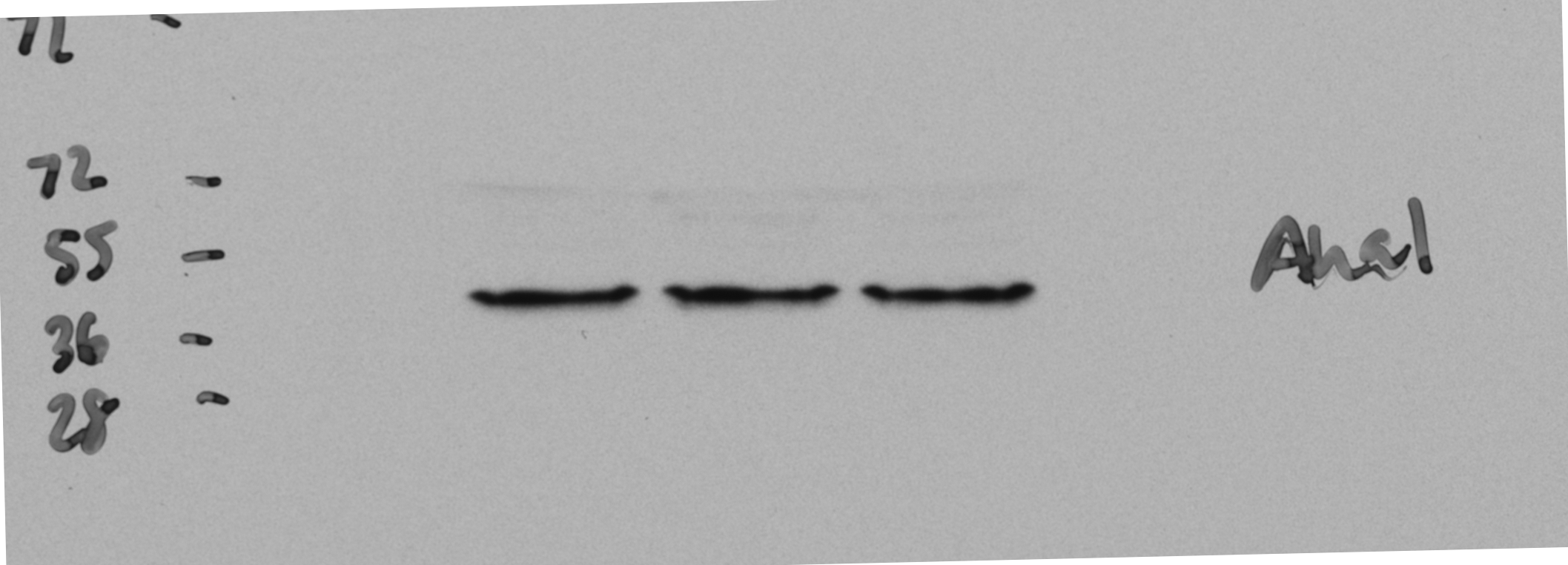

Supplement: Supplementary file 5 — Source data Fig. 2 [file 44319_2024_250_MOESM5_ESM.zip › EMBOR-2024-59387_SourceDataForFigure2/EMBOR-2024-59387_SourceDataForFigure2C/western Aha1.tif]

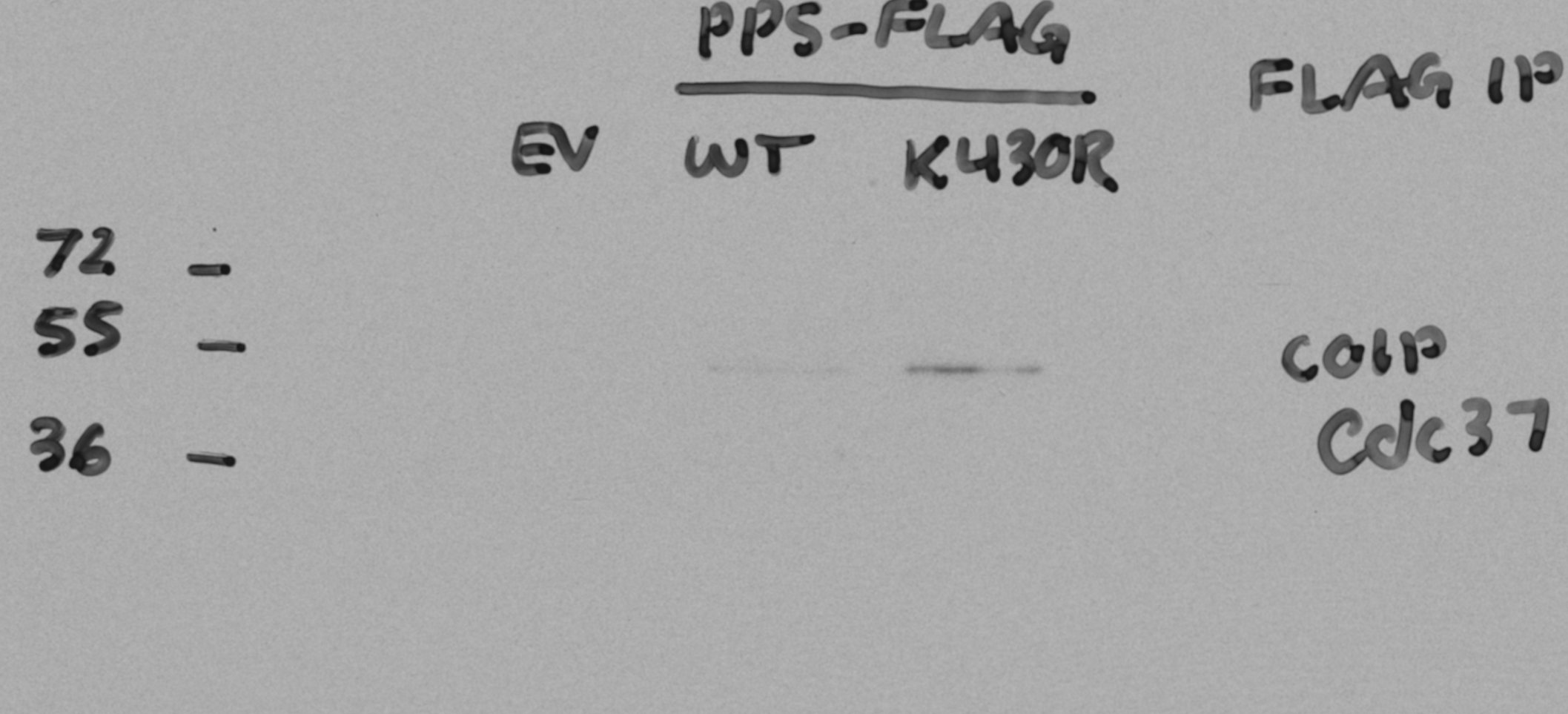

Supplement: Supplementary file 5 — Source data Fig. 2 [file 44319_2024_250_MOESM5_ESM.zip › EMBOR-2024-59387_SourceDataForFigure2/EMBOR-2024-59387_SourceDataForFigure2C/western Cdc37 coIP.tif]

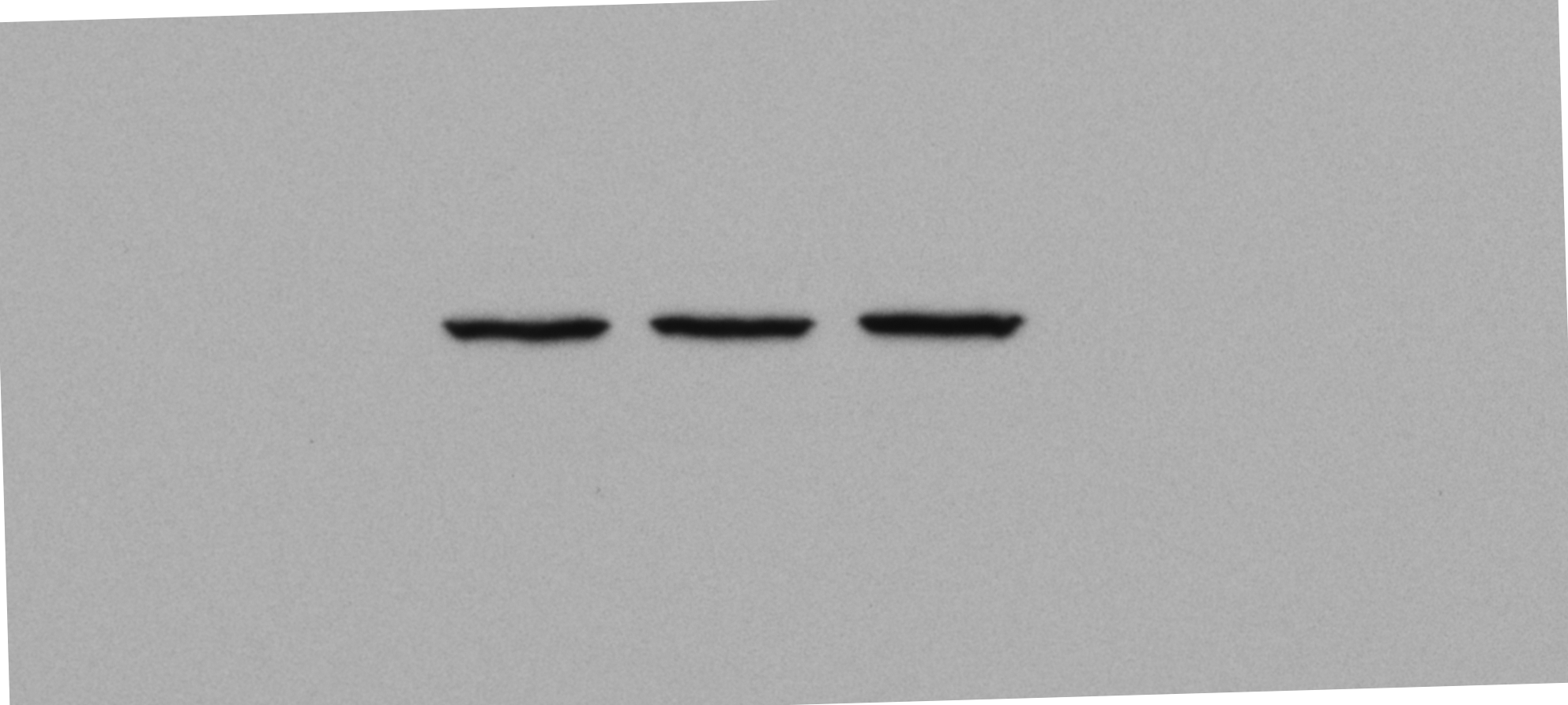

Supplement: Supplementary file 5 — Source data Fig. 2 [file 44319_2024_250_MOESM5_ESM.zip › EMBOR-2024-59387_SourceDataForFigure2/EMBOR-2024-59387_SourceDataForFigure2C/western Cdc37.tif]

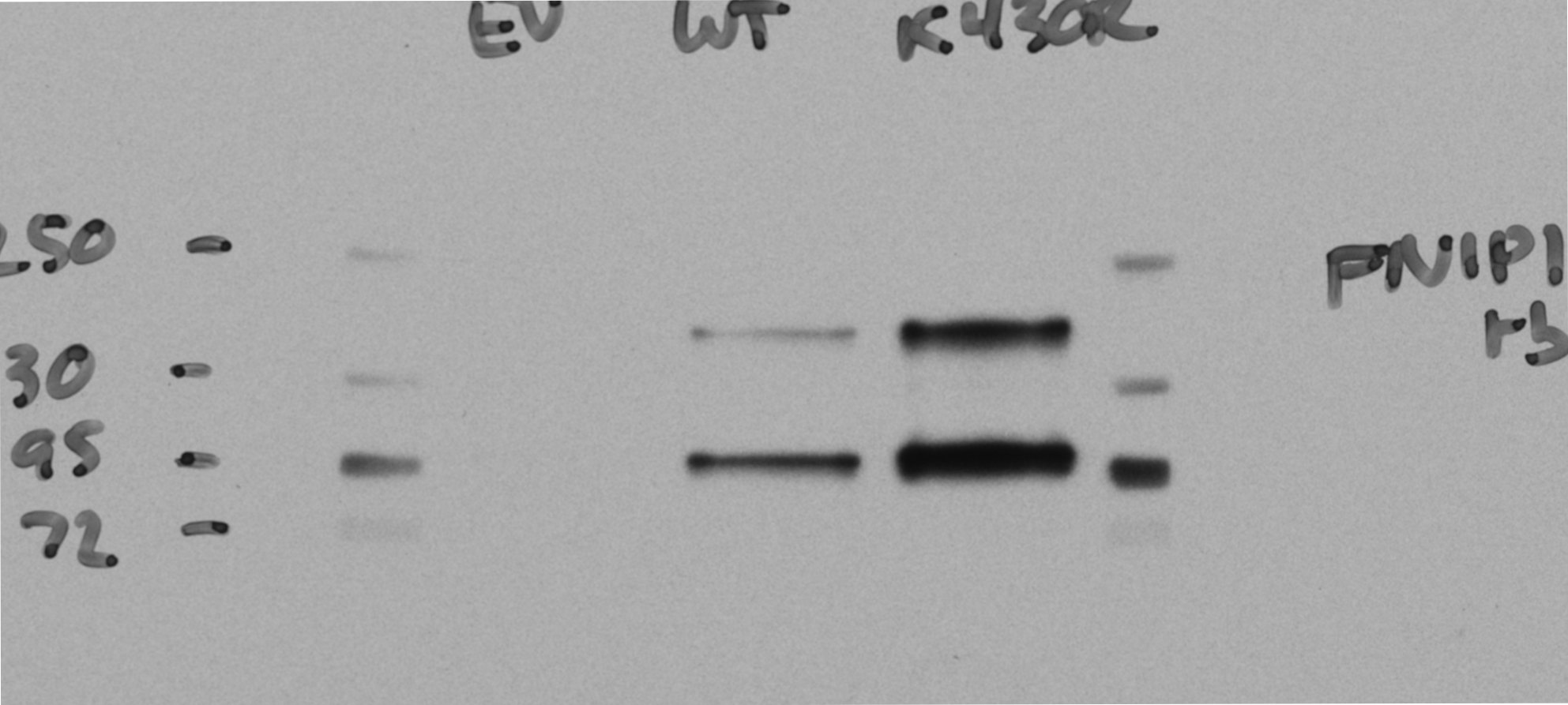

Supplement: Supplementary file 5 — Source data Fig. 2 [file 44319_2024_250_MOESM5_ESM.zip › EMBOR-2024-59387_SourceDataForFigure2/EMBOR-2024-59387_SourceDataForFigure2C/western FNIP1 coIP.tif]

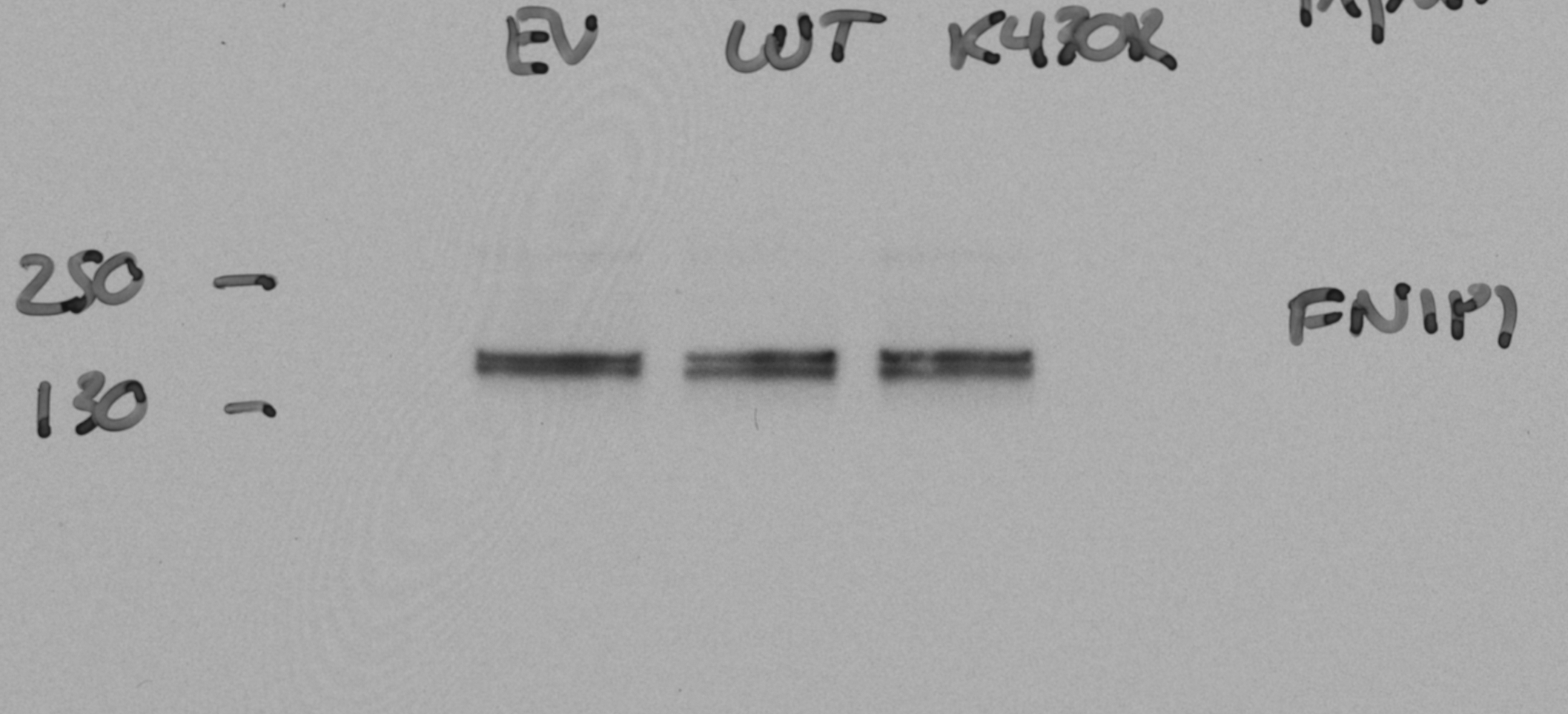

Supplement: Supplementary file 5 — Source data Fig. 2 [file 44319_2024_250_MOESM5_ESM.zip › EMBOR-2024-59387_SourceDataForFigure2/EMBOR-2024-59387_SourceDataForFigure2C/western FNIP1.tif]

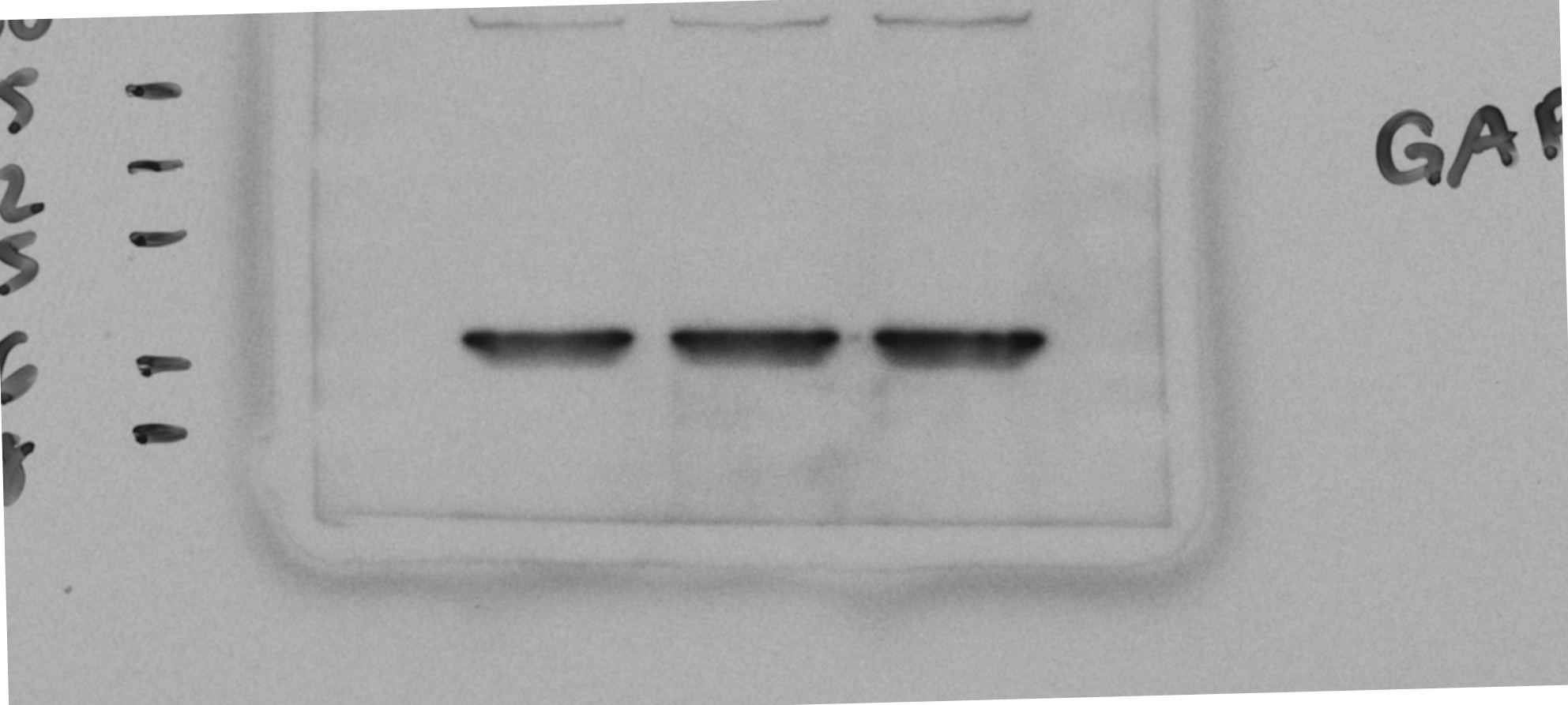

Supplement: Supplementary file 5 — Source data Fig. 2 [file 44319_2024_250_MOESM5_ESM.zip › EMBOR-2024-59387_SourceDataForFigure2/EMBOR-2024-59387_SourceDataForFigure2C/western GAPDH.tif]

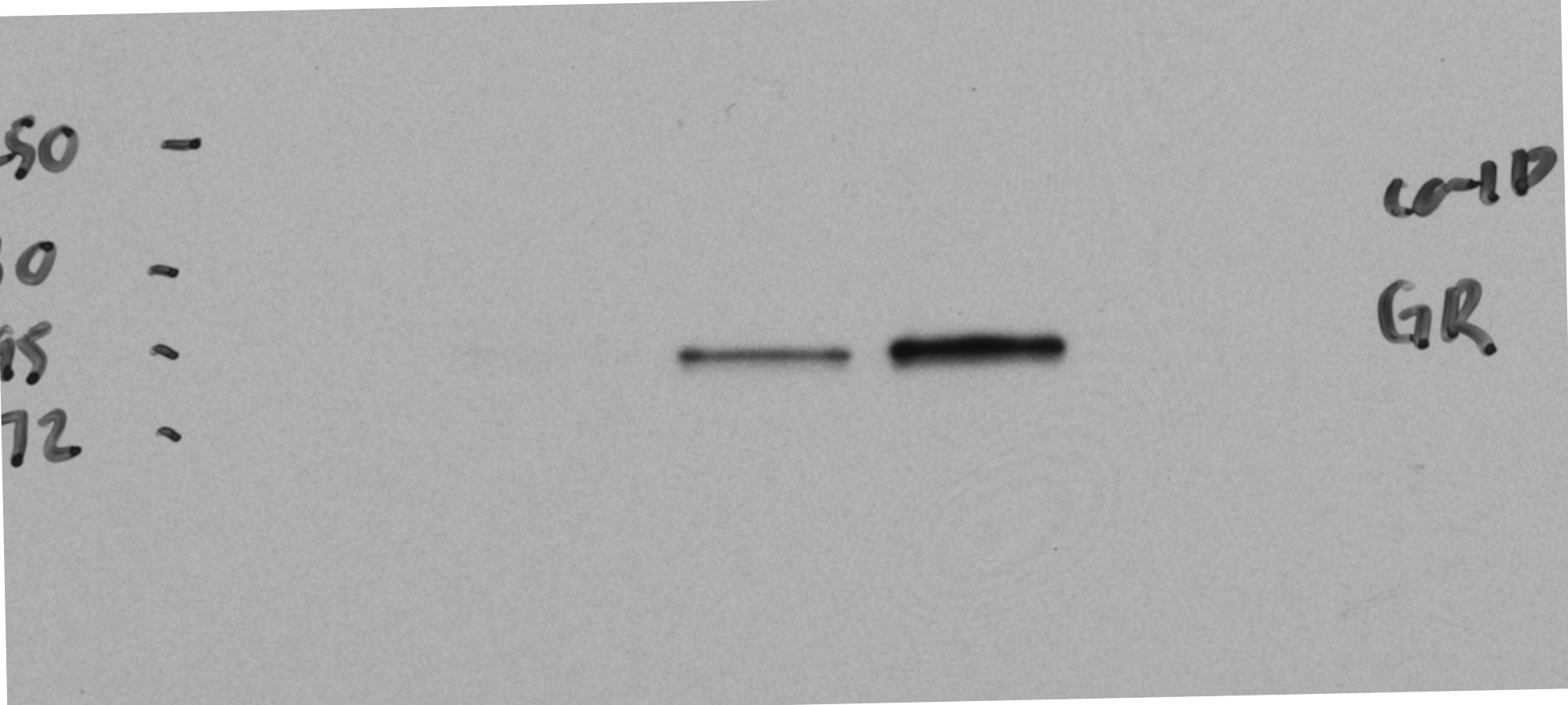

Supplement: Supplementary file 5 — Source data Fig. 2 [file 44319_2024_250_MOESM5_ESM.zip › EMBOR-2024-59387_SourceDataForFigure2/EMBOR-2024-59387_SourceDataForFigure2C/western GR coIP.tif]

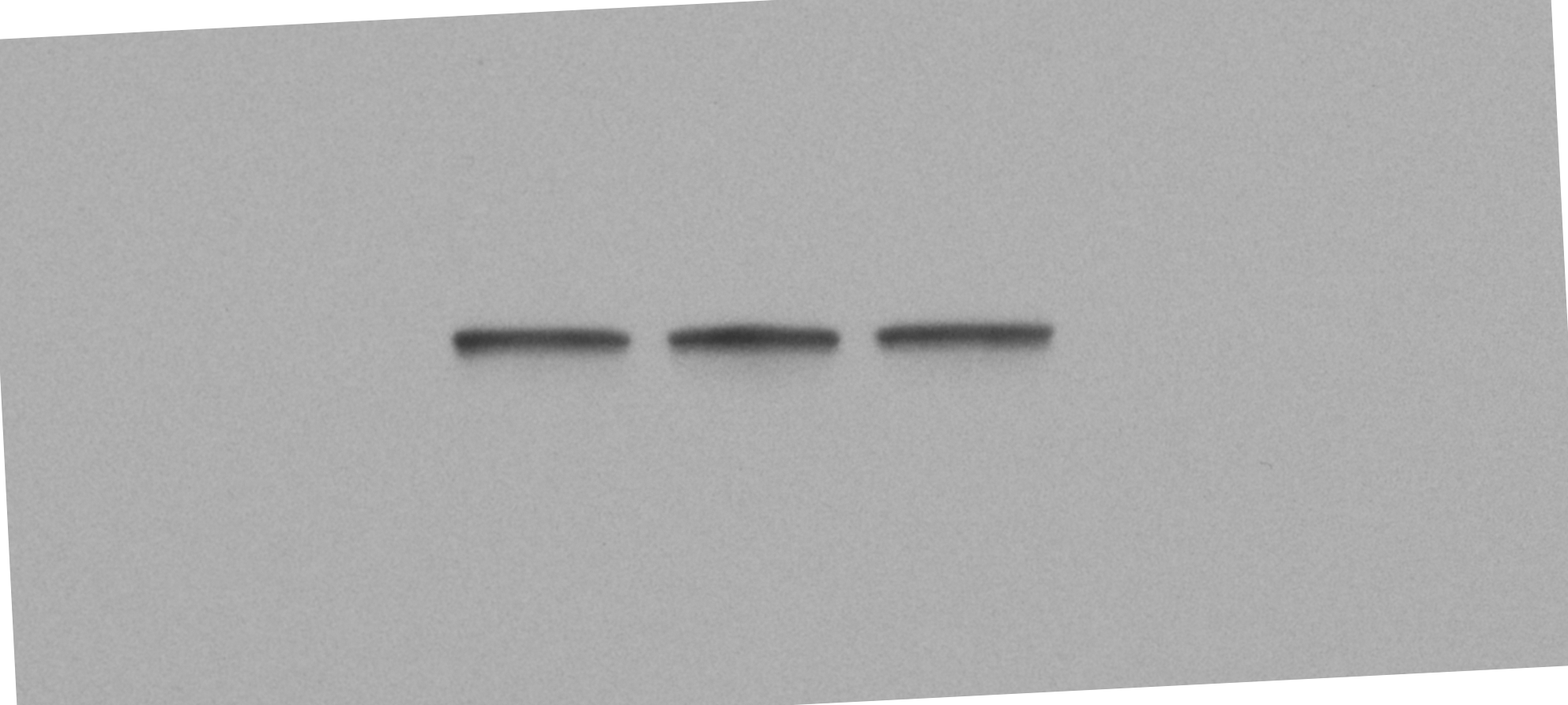

Supplement: Supplementary file 5 — Source data Fig. 2 [file 44319_2024_250_MOESM5_ESM.zip › EMBOR-2024-59387_SourceDataForFigure2/EMBOR-2024-59387_SourceDataForFigure2C/western GR.tif]

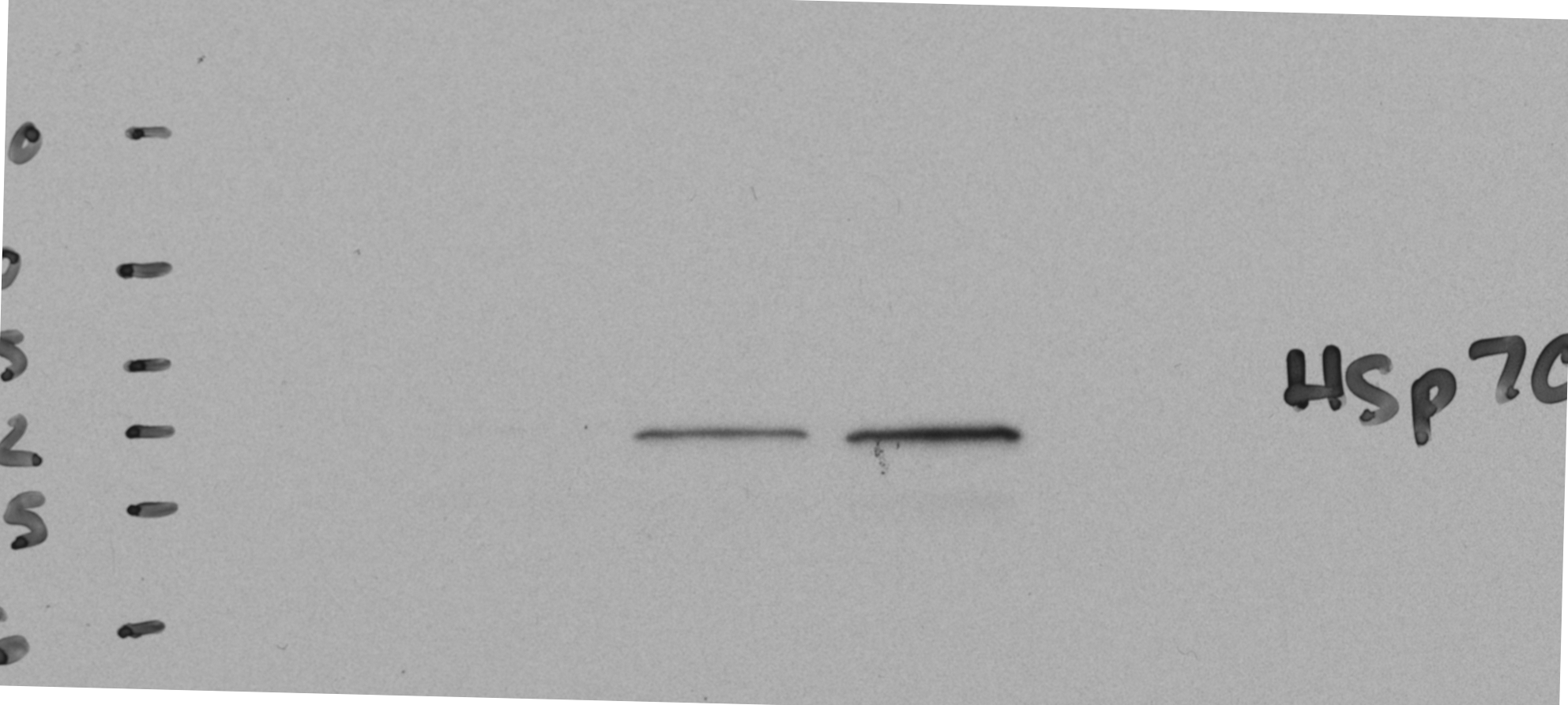

Supplement: Supplementary file 5 — Source data Fig. 2 [file 44319_2024_250_MOESM5_ESM.zip › EMBOR-2024-59387_SourceDataForFigure2/EMBOR-2024-59387_SourceDataForFigure2C/western Hsp70 coIP.tif]

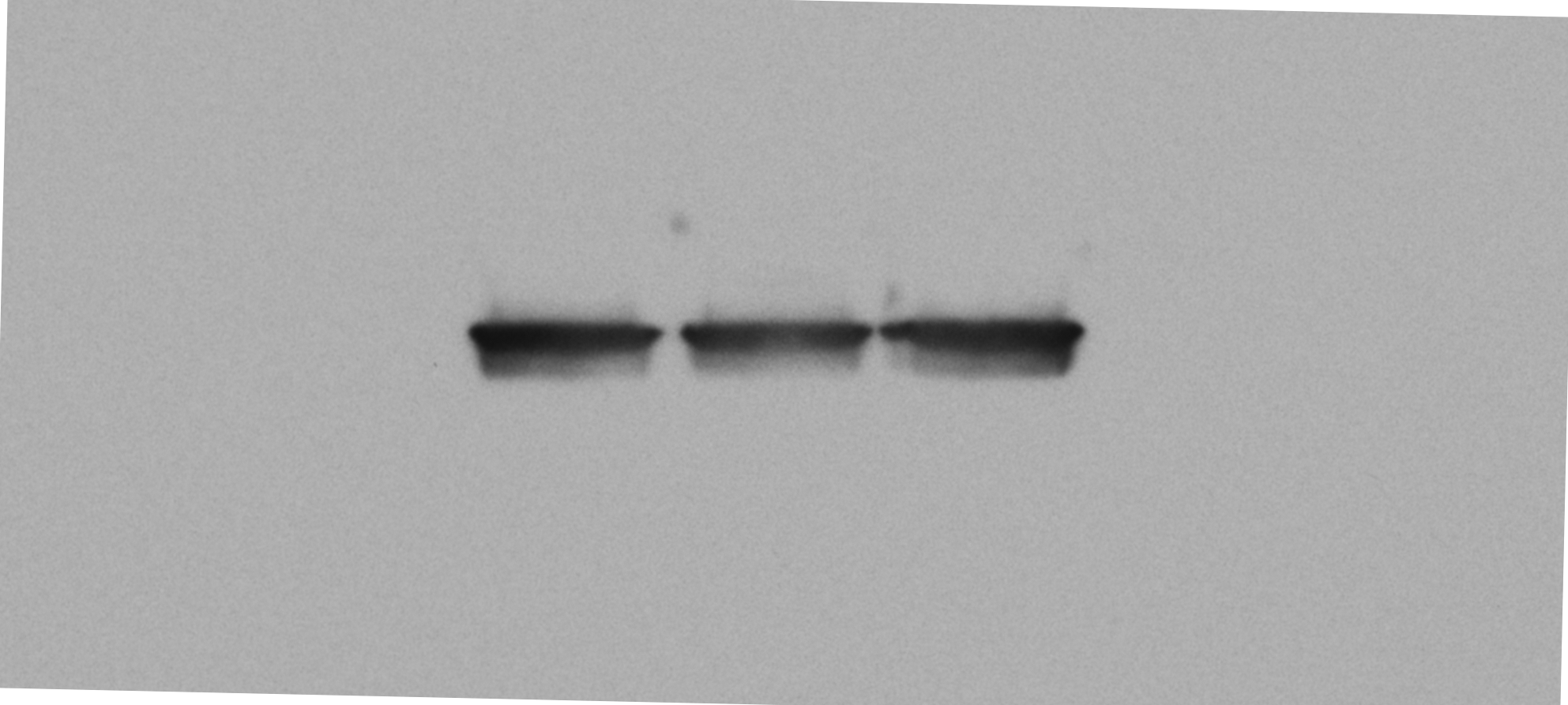

Supplement: Supplementary file 5 — Source data Fig. 2 [file 44319_2024_250_MOESM5_ESM.zip › EMBOR-2024-59387_SourceDataForFigure2/EMBOR-2024-59387_SourceDataForFigure2C/western Hsp70.tif]

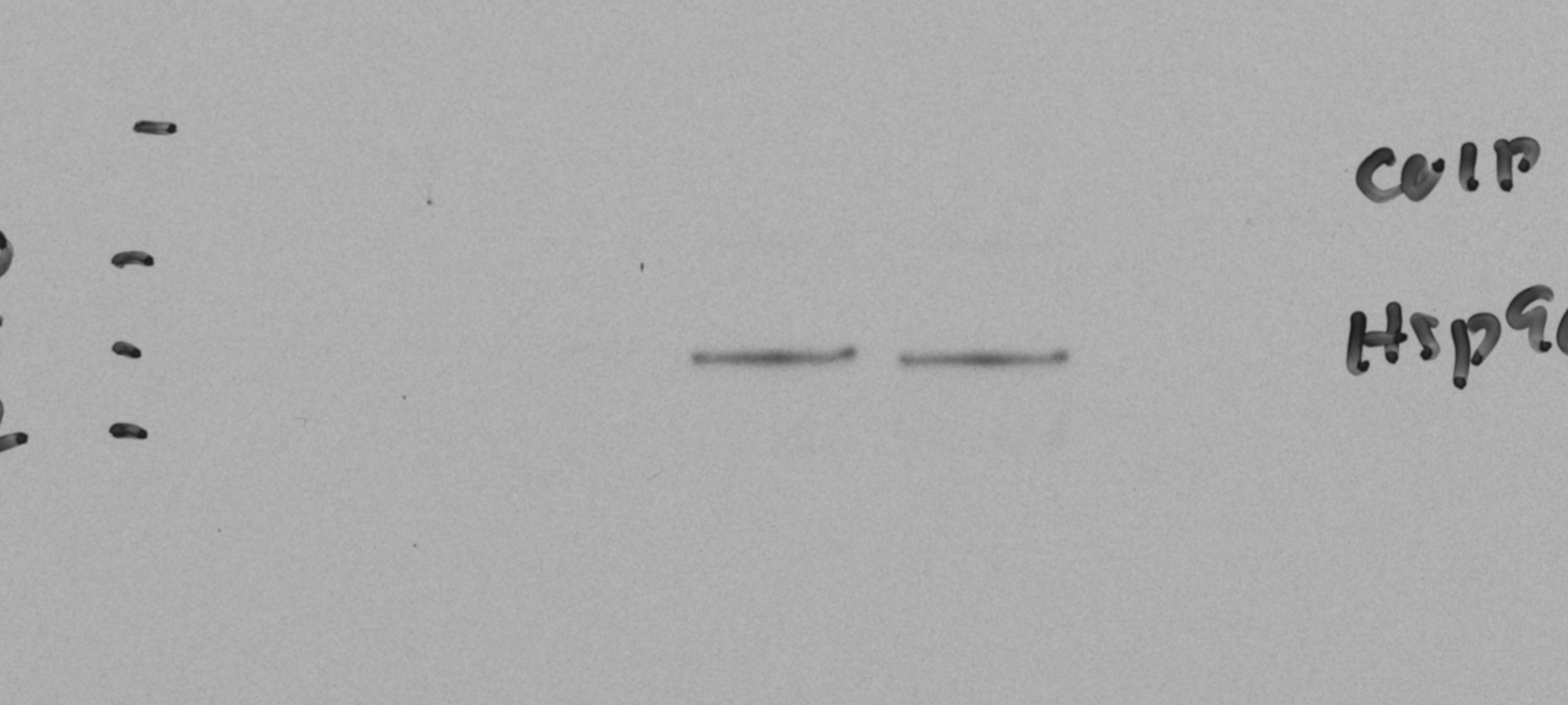

Supplement: Supplementary file 5 — Source data Fig. 2 [file 44319_2024_250_MOESM5_ESM.zip › EMBOR-2024-59387_SourceDataForFigure2/EMBOR-2024-59387_SourceDataForFigure2C/western Hsp90 coIP.tif]

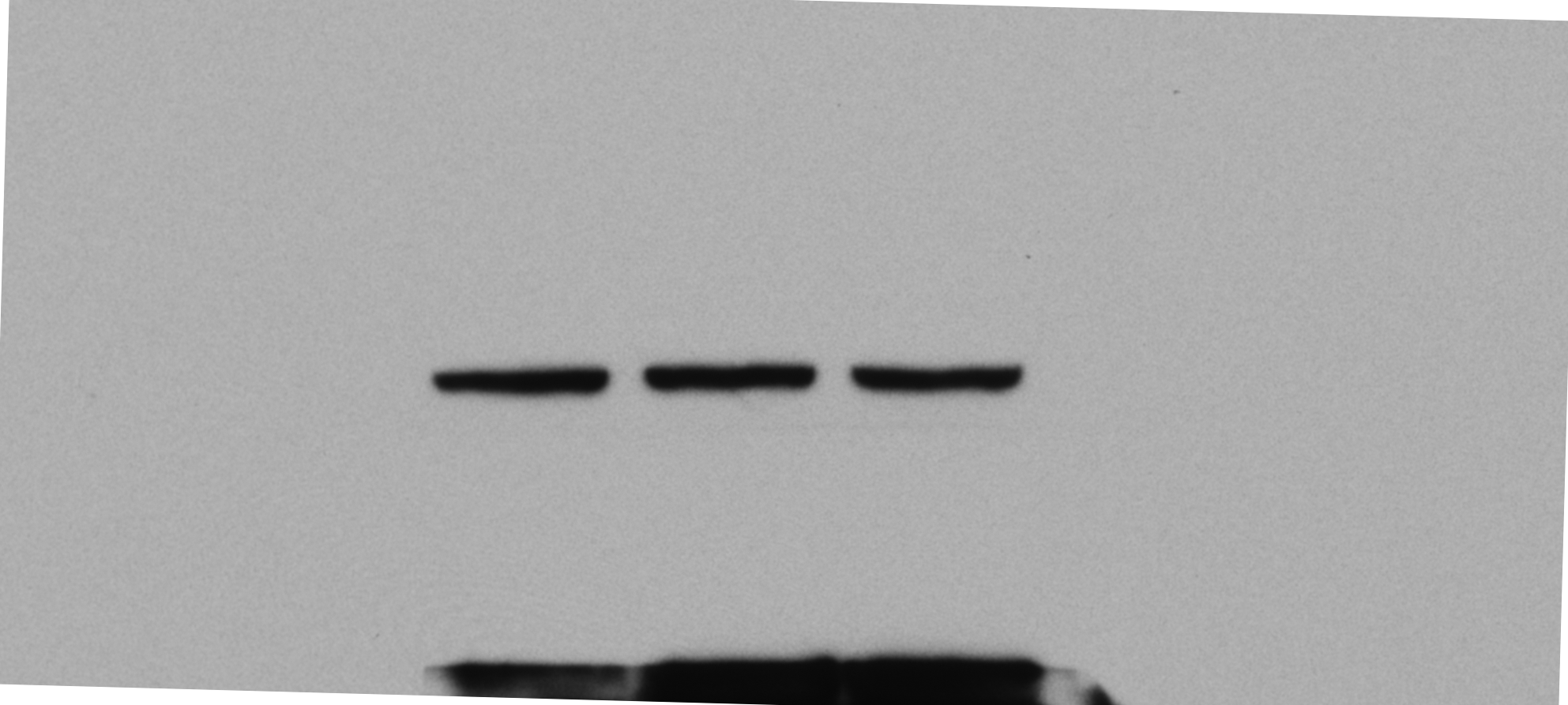

Supplement: Supplementary file 5 — Source data Fig. 2 [file 44319_2024_250_MOESM5_ESM.zip › EMBOR-2024-59387_SourceDataForFigure2/EMBOR-2024-59387_SourceDataForFigure2C/western Hsp90.tif]

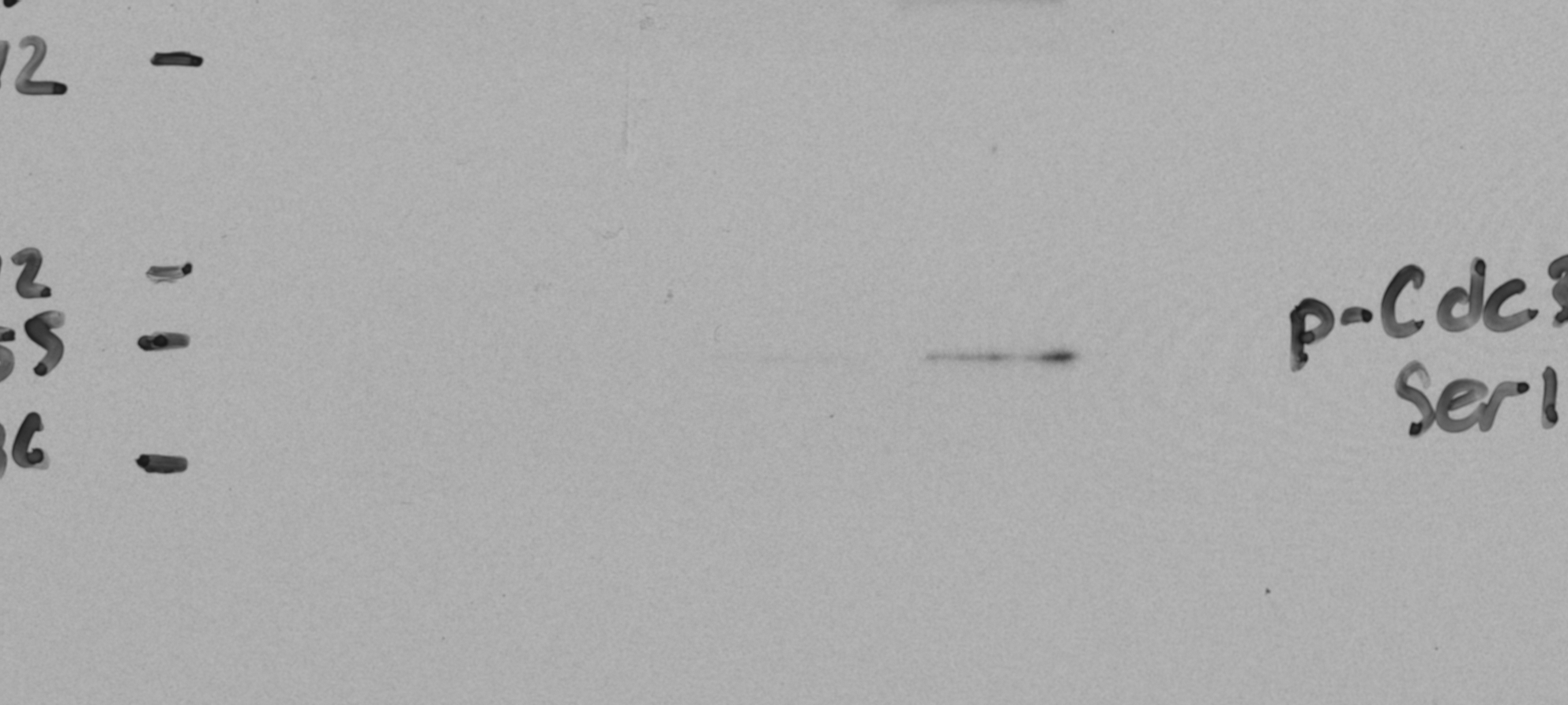

Supplement: Supplementary file 5 — Source data Fig. 2 [file 44319_2024_250_MOESM5_ESM.zip › EMBOR-2024-59387_SourceDataForFigure2/EMBOR-2024-59387_SourceDataForFigure2C/western phos-Ser13-Cdc37 coIP.tif]

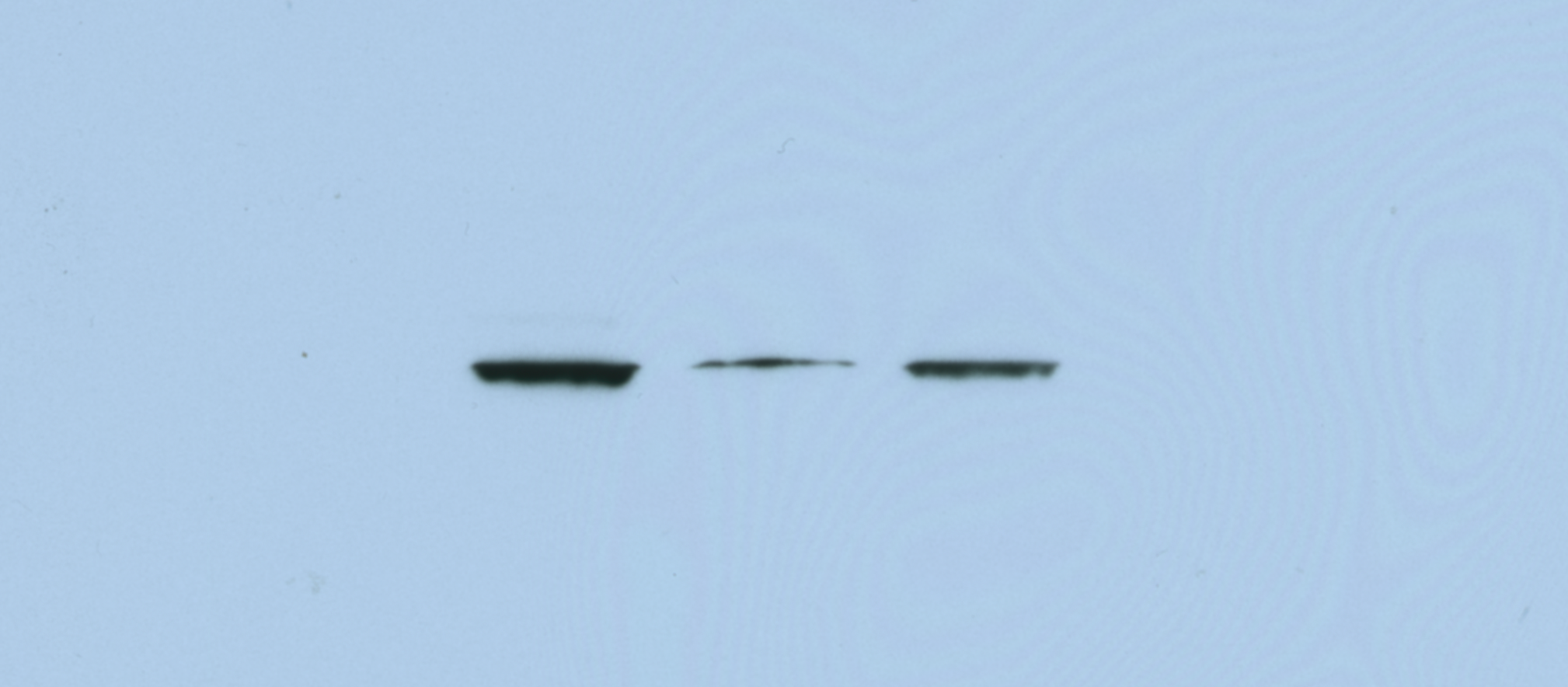

Supplement: Supplementary file 5 — Source data Fig. 2 [file 44319_2024_250_MOESM5_ESM.zip › EMBOR-2024-59387_SourceDataForFigure2/EMBOR-2024-59387_SourceDataForFigure2C/western phos-Ser13-Cdc37.tif]

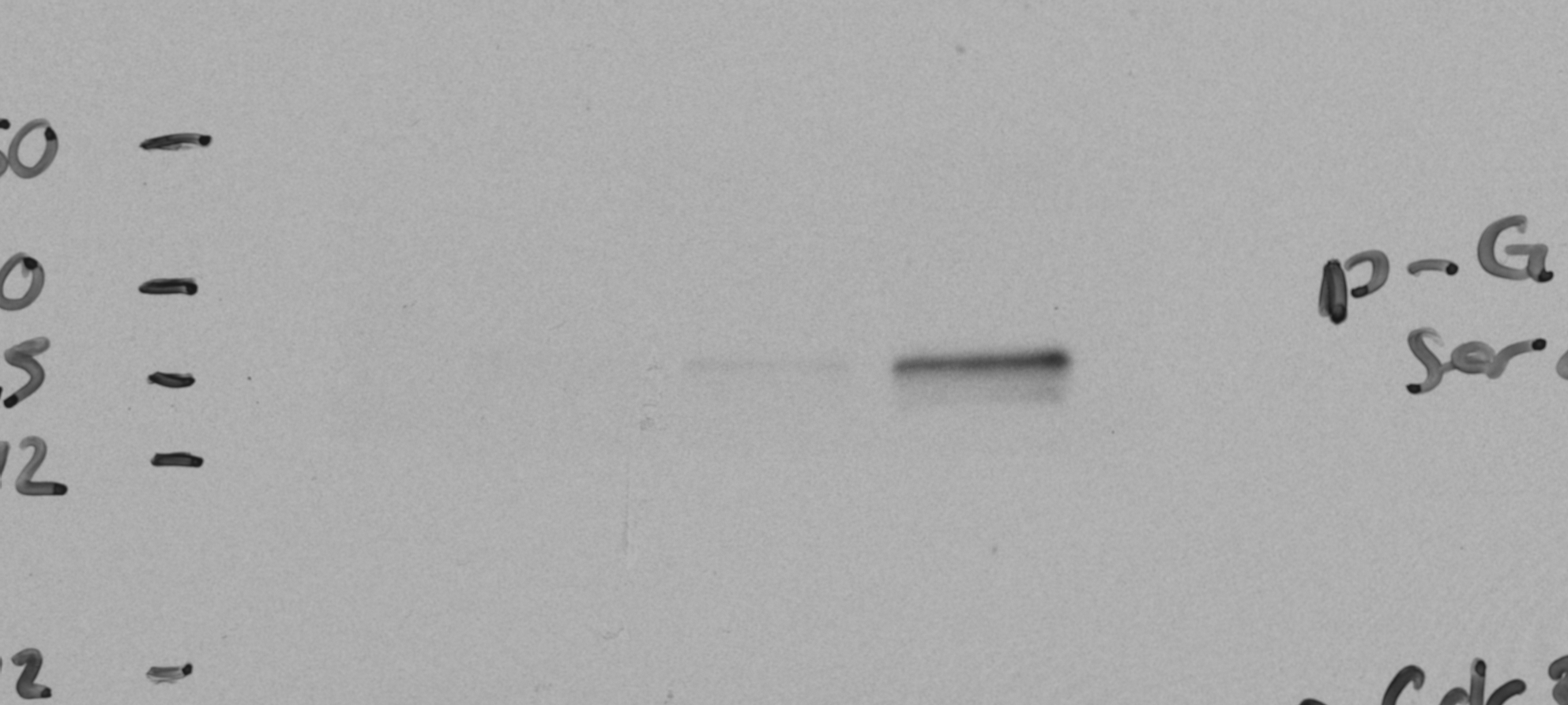

Supplement: Supplementary file 5 — Source data Fig. 2 [file 44319_2024_250_MOESM5_ESM.zip › EMBOR-2024-59387_SourceDataForFigure2/EMBOR-2024-59387_SourceDataForFigure2C/western phos-Ser211-GR coIP.tif]

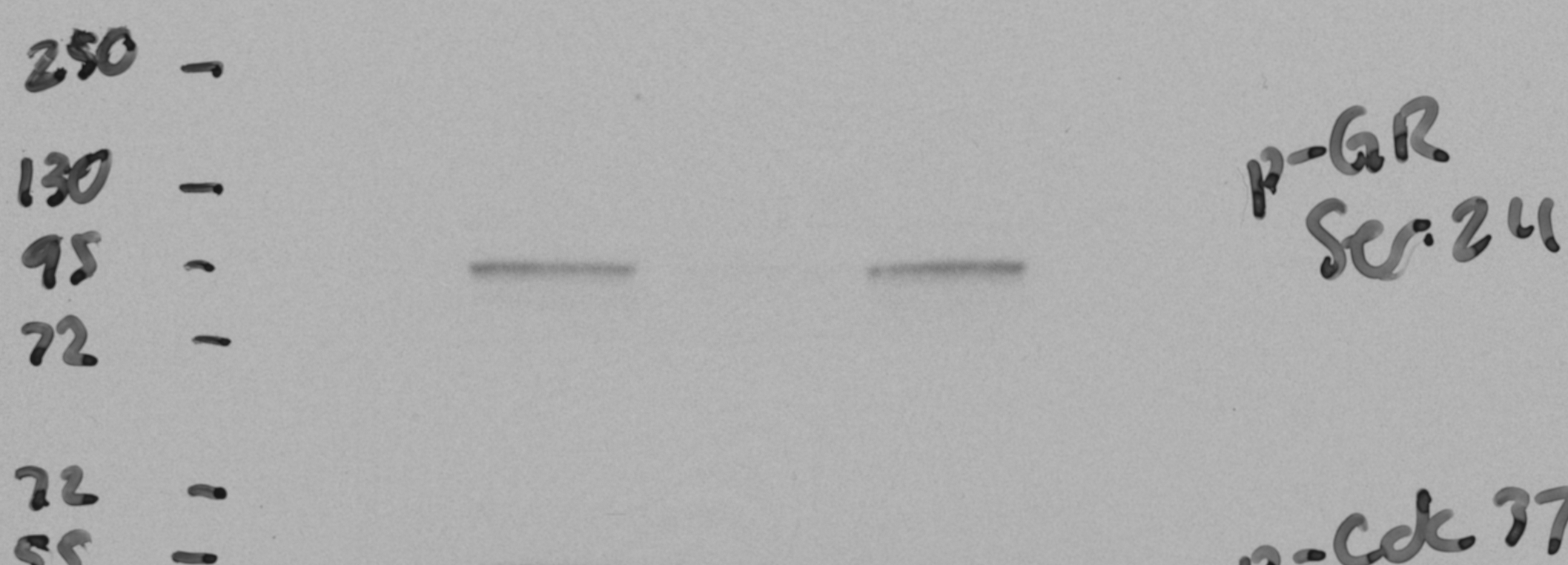

Supplement: Supplementary file 5 — Source data Fig. 2 [file 44319_2024_250_MOESM5_ESM.zip › EMBOR-2024-59387_SourceDataForFigure2/EMBOR-2024-59387_SourceDataForFigure2C/western phos-Ser211-GR.tif]

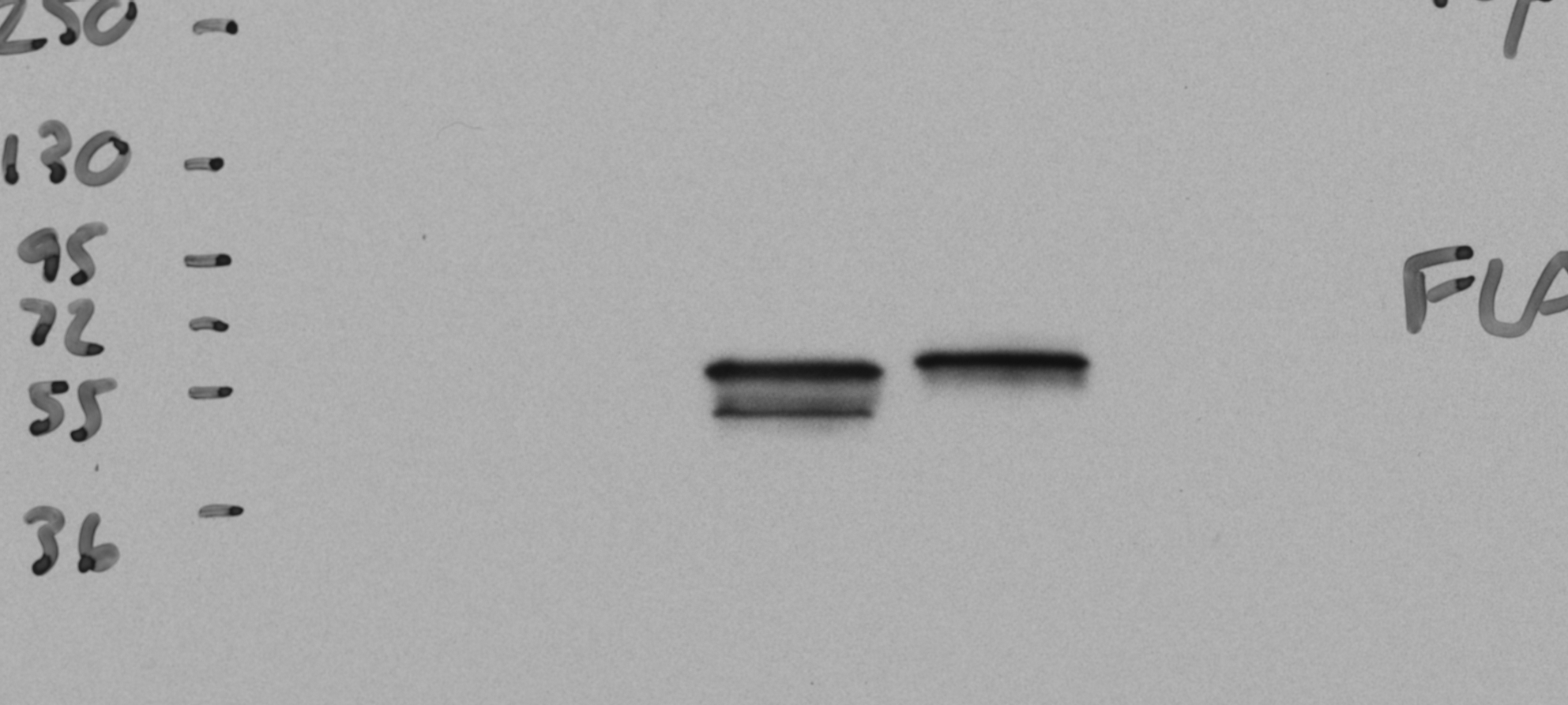

Supplement: Supplementary file 5 — Source data Fig. 2 [file 44319_2024_250_MOESM5_ESM.zip › EMBOR-2024-59387_SourceDataForFigure2/EMBOR-2024-59387_SourceDataForFigure2C/western PP5-FLAG IP.tif]

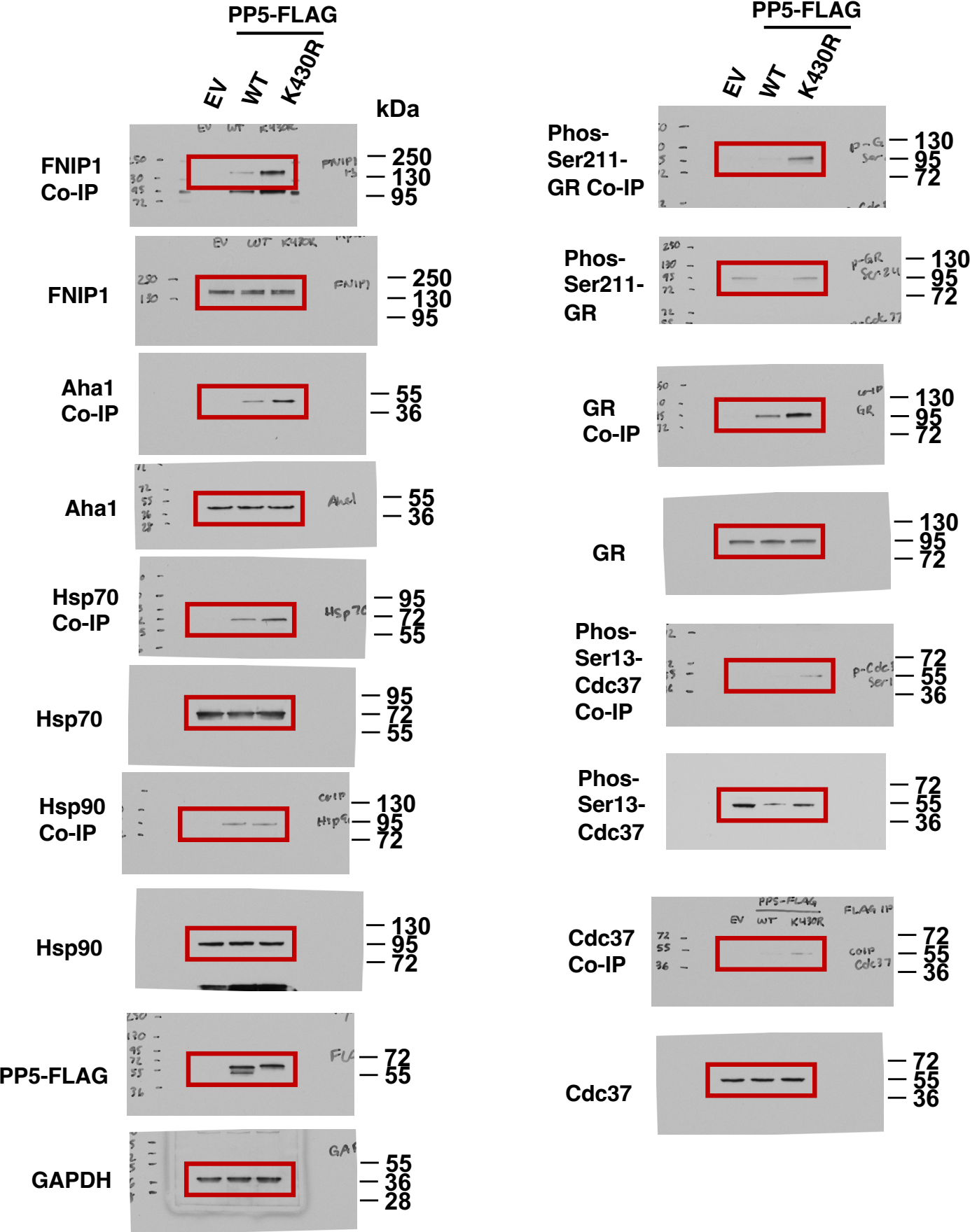

Supplement: Supplementary file 5 — Source data Fig. 2 [file 44319_2024_250_MOESM5_ESM.zip › EMBOR-2024-59387_SourceDataForFigure2/EMBOR-2024-59387_SourceDataForFigure2C/western uncropped annotated.pdf]

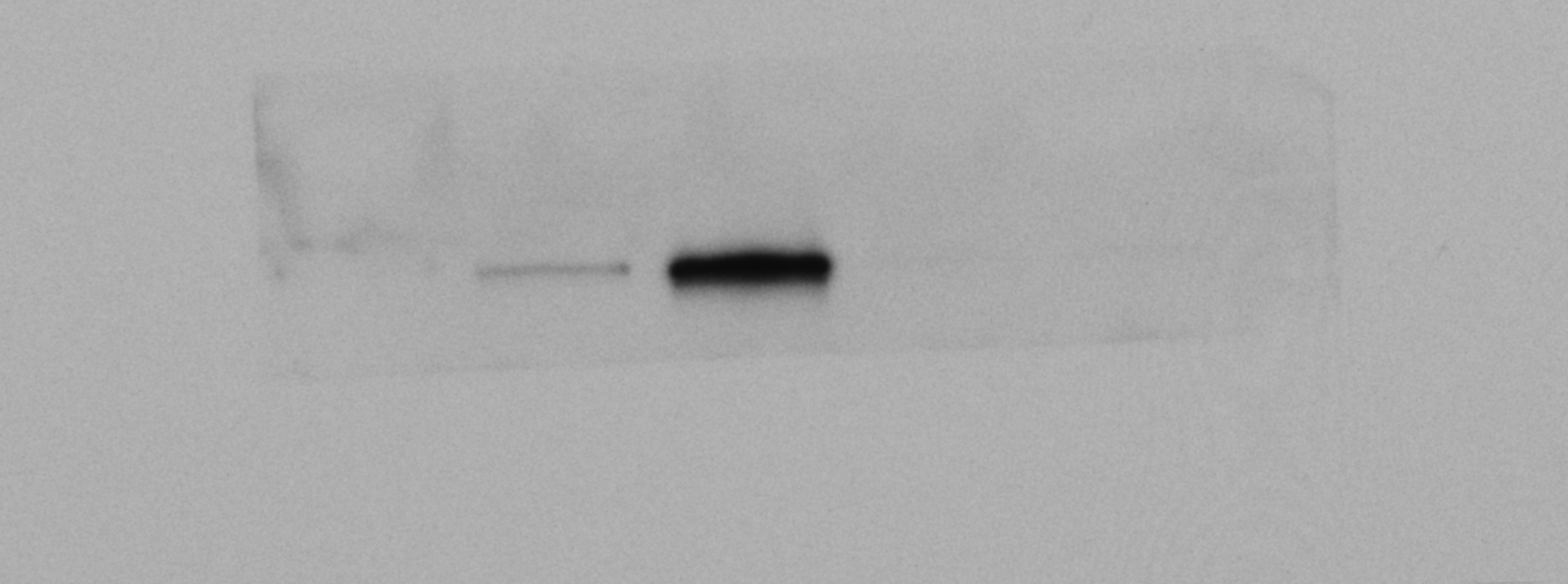

Supplement: Supplementary file 5 — Source data Fig. 2 [file 44319_2024_250_MOESM5_ESM.zip › EMBOR-2024-59387_SourceDataForFigure2/EMBOR-2024-59387_SourceDataForFigure2D/western GR coIP.tif]

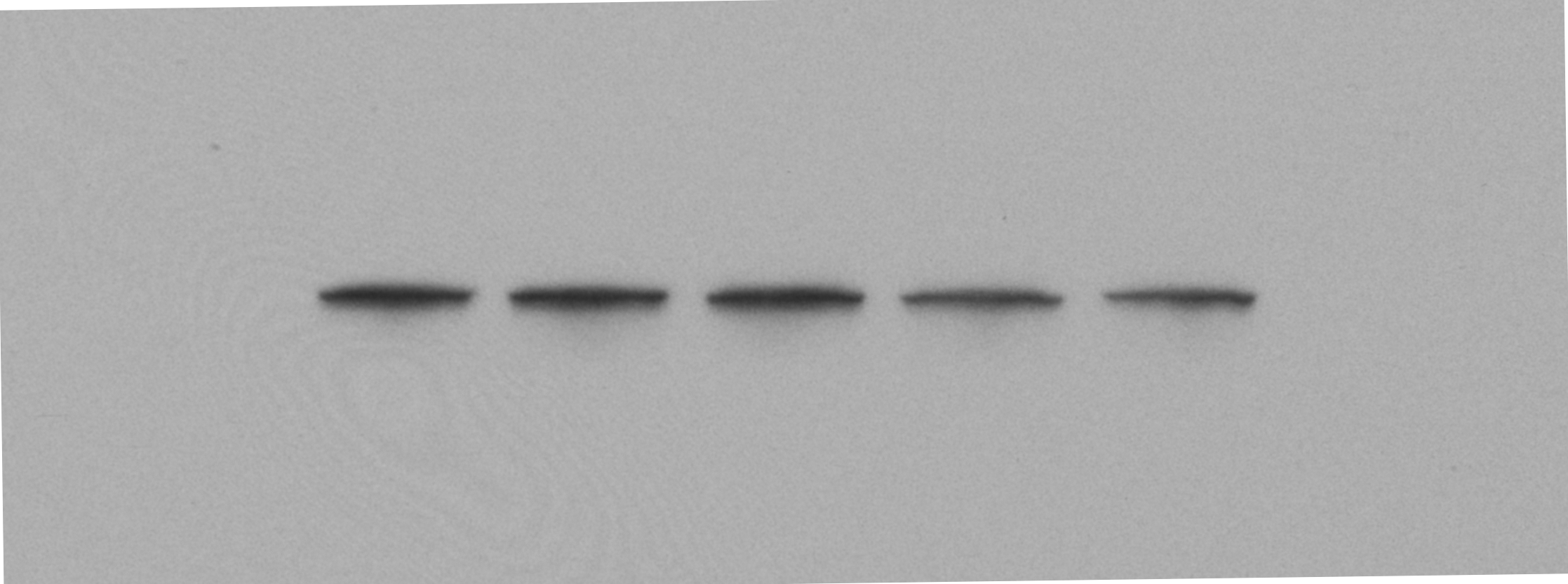

Supplement: Supplementary file 5 — Source data Fig. 2 [file 44319_2024_250_MOESM5_ESM.zip › EMBOR-2024-59387_SourceDataForFigure2/EMBOR-2024-59387_SourceDataForFigure2D/western GR.tif]

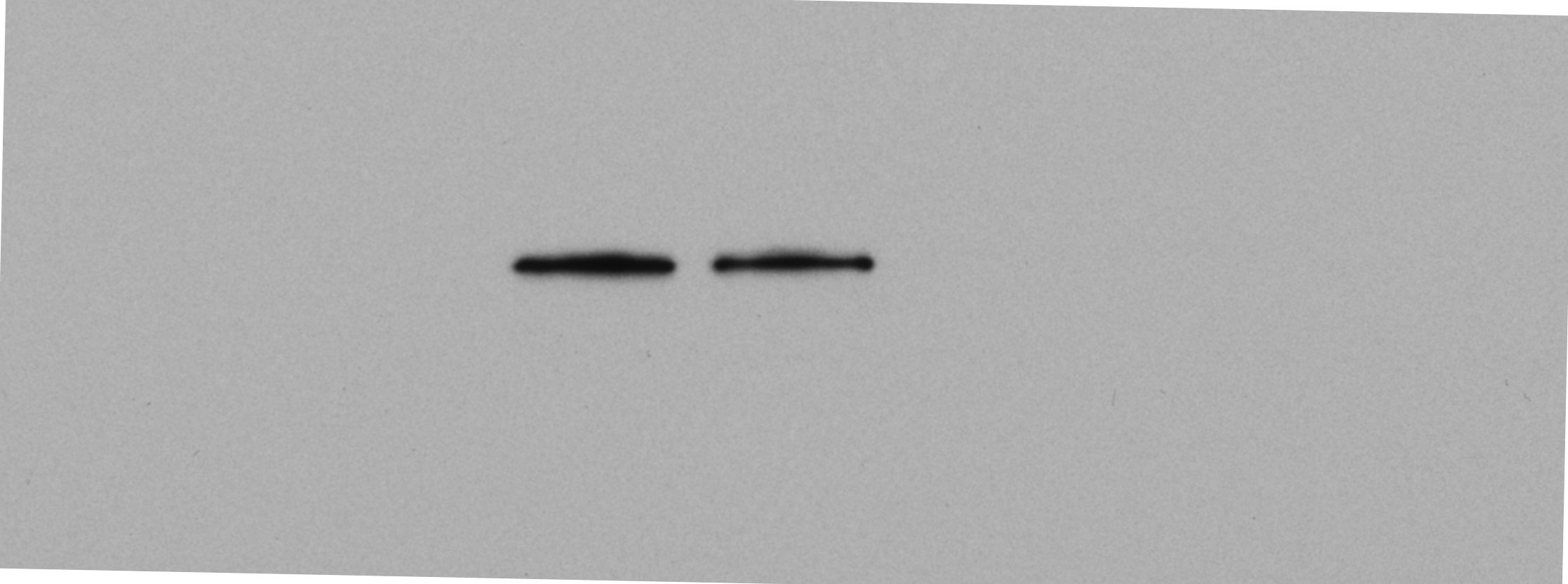

Supplement: Supplementary file 5 — Source data Fig. 2 [file 44319_2024_250_MOESM5_ESM.zip › EMBOR-2024-59387_SourceDataForFigure2/EMBOR-2024-59387_SourceDataForFigure2D/western Hsp90 coIP.tif]

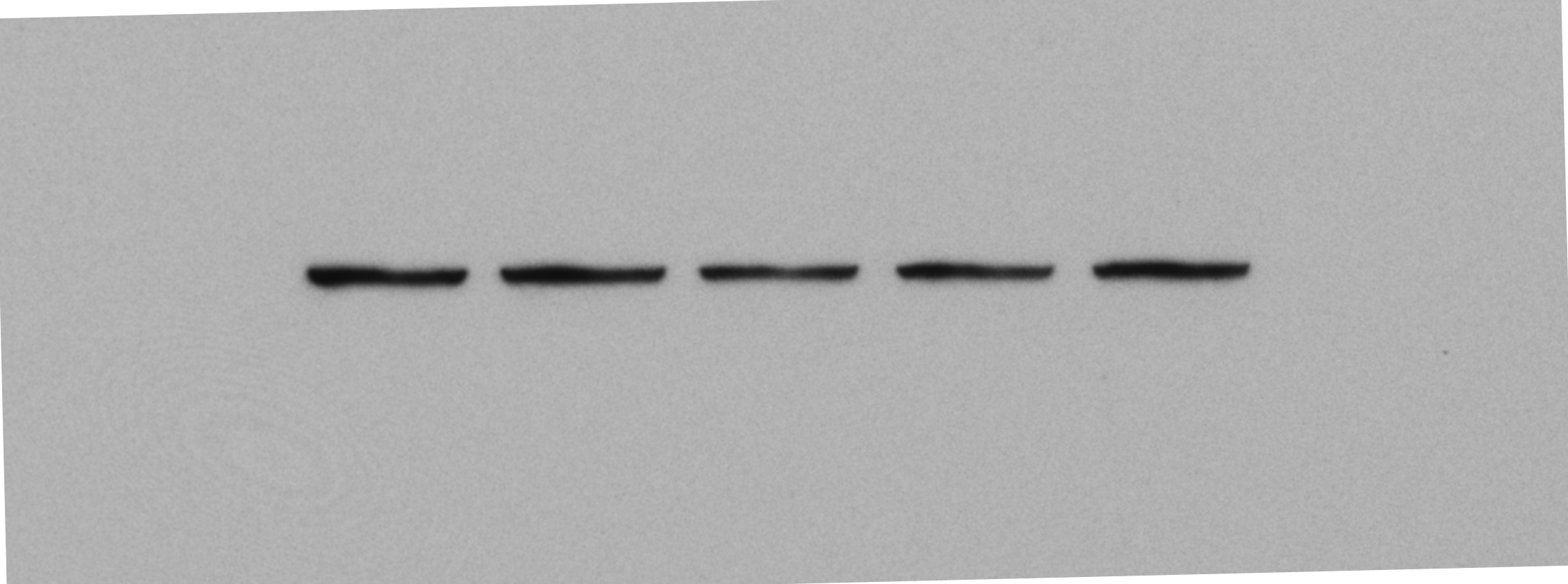

Supplement: Supplementary file 5 — Source data Fig. 2 [file 44319_2024_250_MOESM5_ESM.zip › EMBOR-2024-59387_SourceDataForFigure2/EMBOR-2024-59387_SourceDataForFigure2D/western Hsp90.tif]

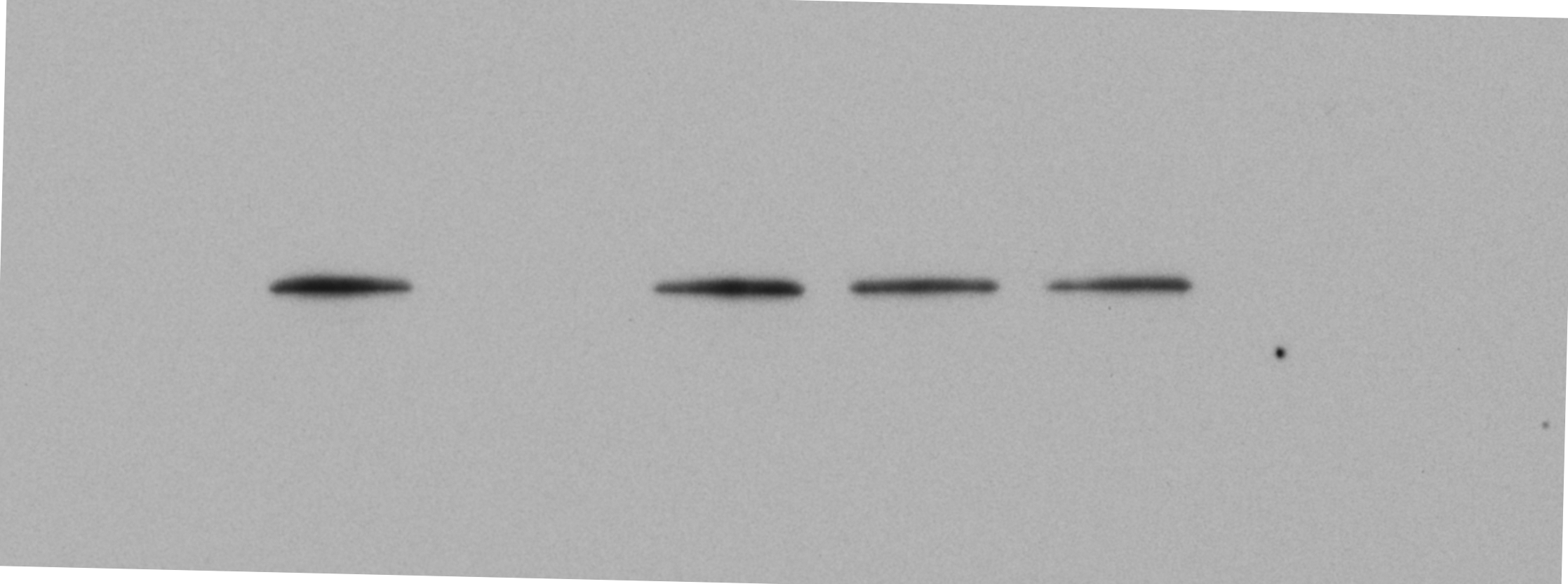

Supplement: Supplementary file 5 — Source data Fig. 2 [file 44319_2024_250_MOESM5_ESM.zip › EMBOR-2024-59387_SourceDataForFigure2/EMBOR-2024-59387_SourceDataForFigure2D/western phos-GR Ser211.tif]

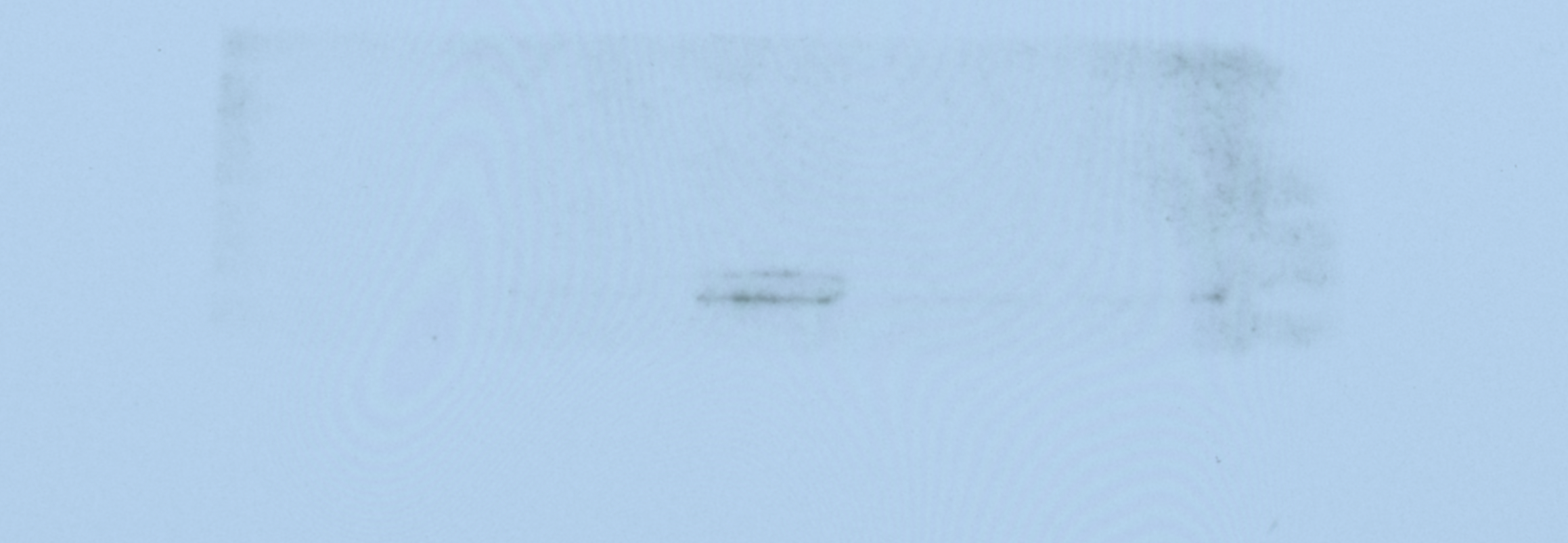

Supplement: Supplementary file 5 — Source data Fig. 2 [file 44319_2024_250_MOESM5_ESM.zip › EMBOR-2024-59387_SourceDataForFigure2/EMBOR-2024-59387_SourceDataForFigure2D/western phosGR S211 coIP.tif]

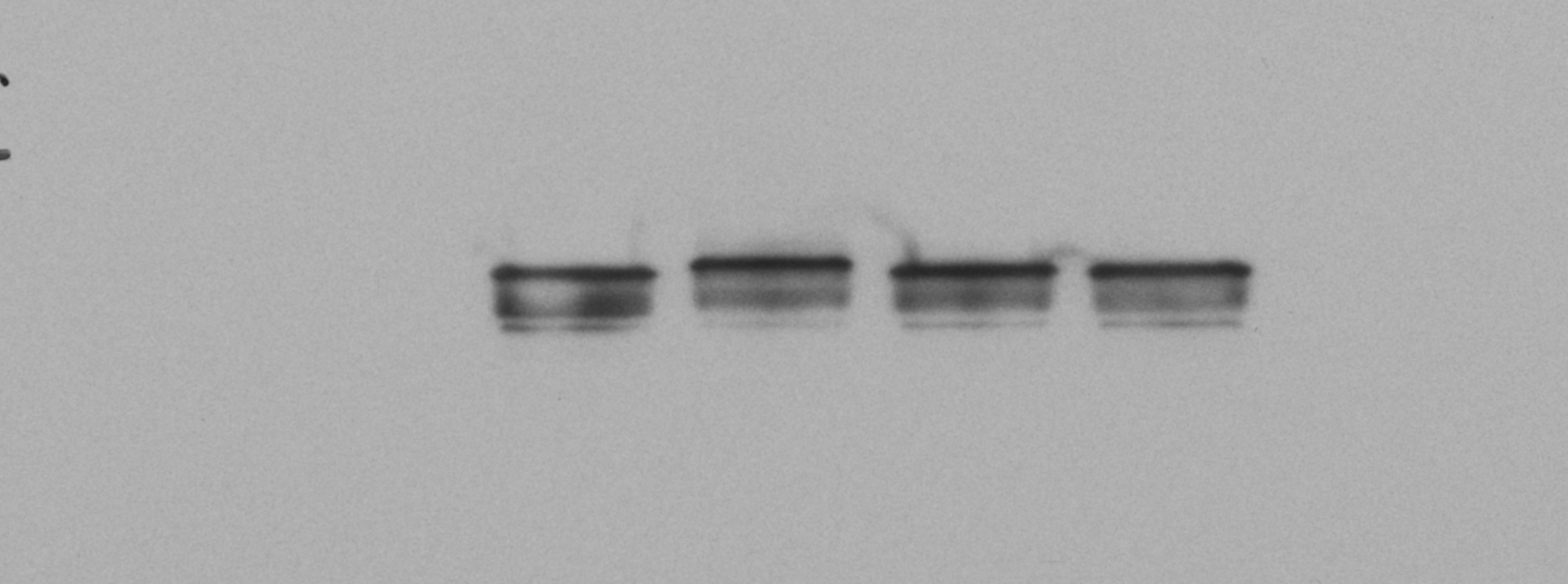

Supplement: Supplementary file 5 — Source data Fig. 2 [file 44319_2024_250_MOESM5_ESM.zip › EMBOR-2024-59387_SourceDataForFigure2/EMBOR-2024-59387_SourceDataForFigure2D/western PP5-FLAG IP.tif]

2D

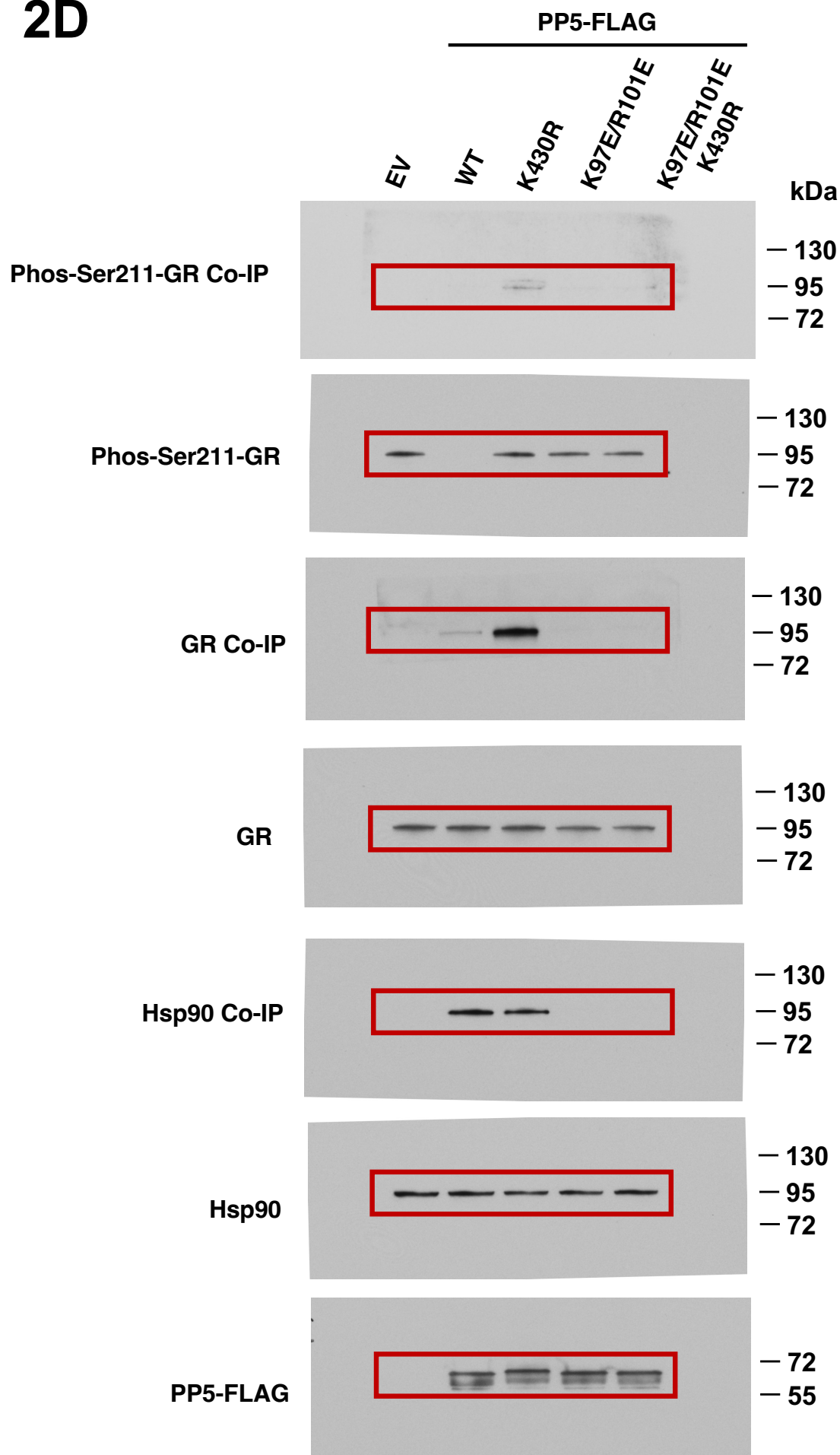

Supplement: Supplementary file 5 — Source data Fig. 2 [file 44319_2024_250_MOESM5_ESM.zip › EMBOR-2024-59387_SourceDataForFigure2/EMBOR-2024-59387_SourceDataForFigure2D/western uncropped annotated.pdf]

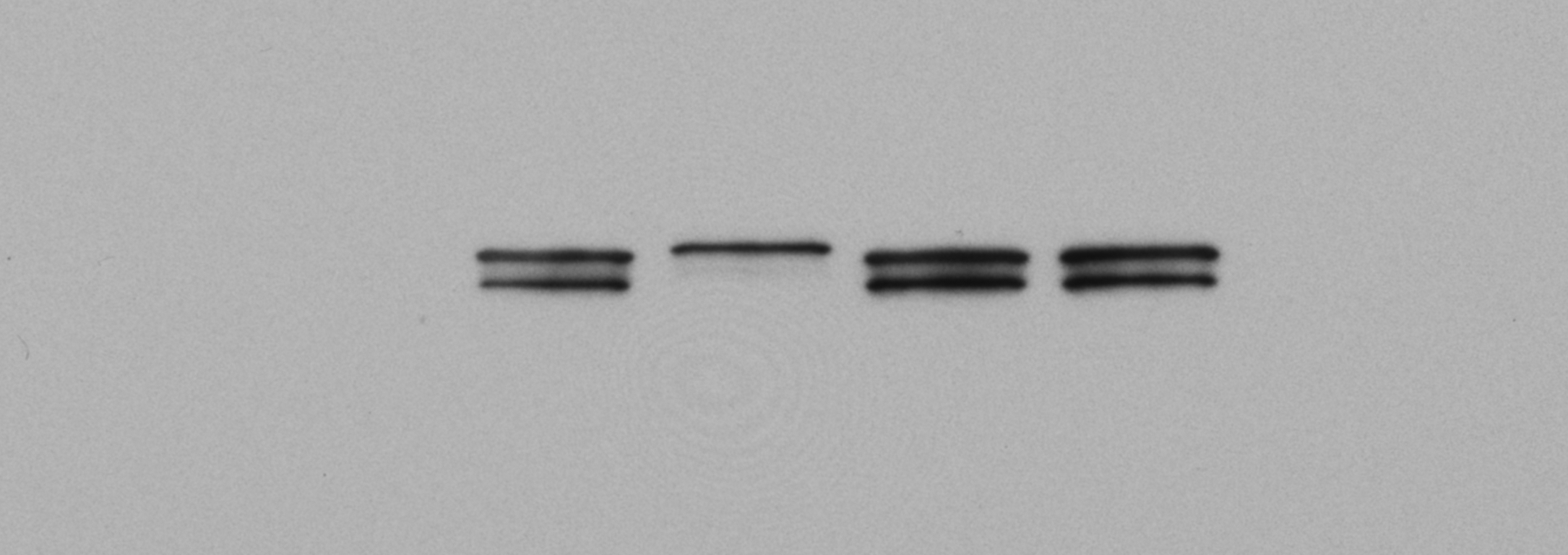

Supplement: Supplementary file 5 — Source data Fig. 2 [file 44319_2024_250_MOESM5_ESM.zip › EMBOR-2024-59387_SourceDataForFigure2/EMBOR-2024-59387_SourceDataForFigure2E/western FLAG IP.tif]

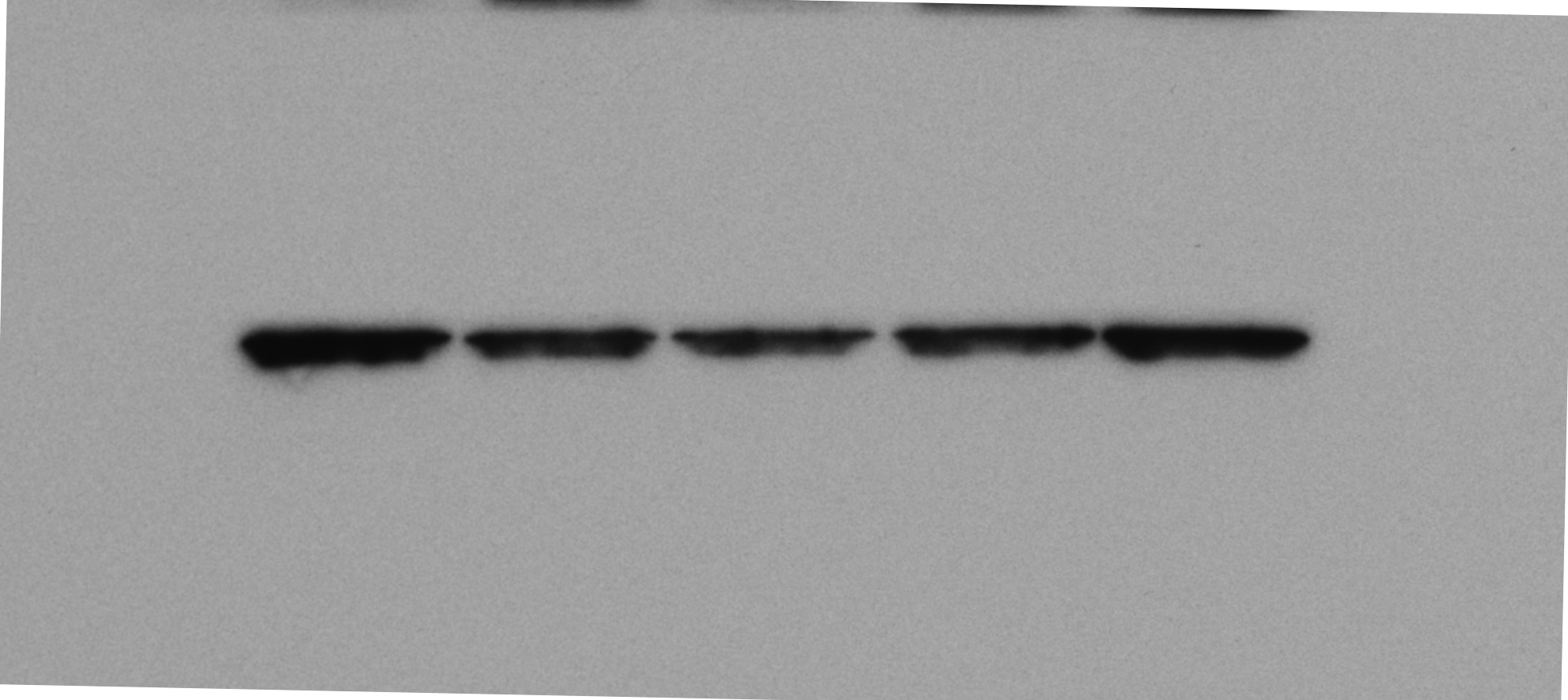

Supplement: Supplementary file 5 — Source data Fig. 2 [file 44319_2024_250_MOESM5_ESM.zip › EMBOR-2024-59387_SourceDataForFigure2/EMBOR-2024-59387_SourceDataForFigure2E/western GAPDH.tif]

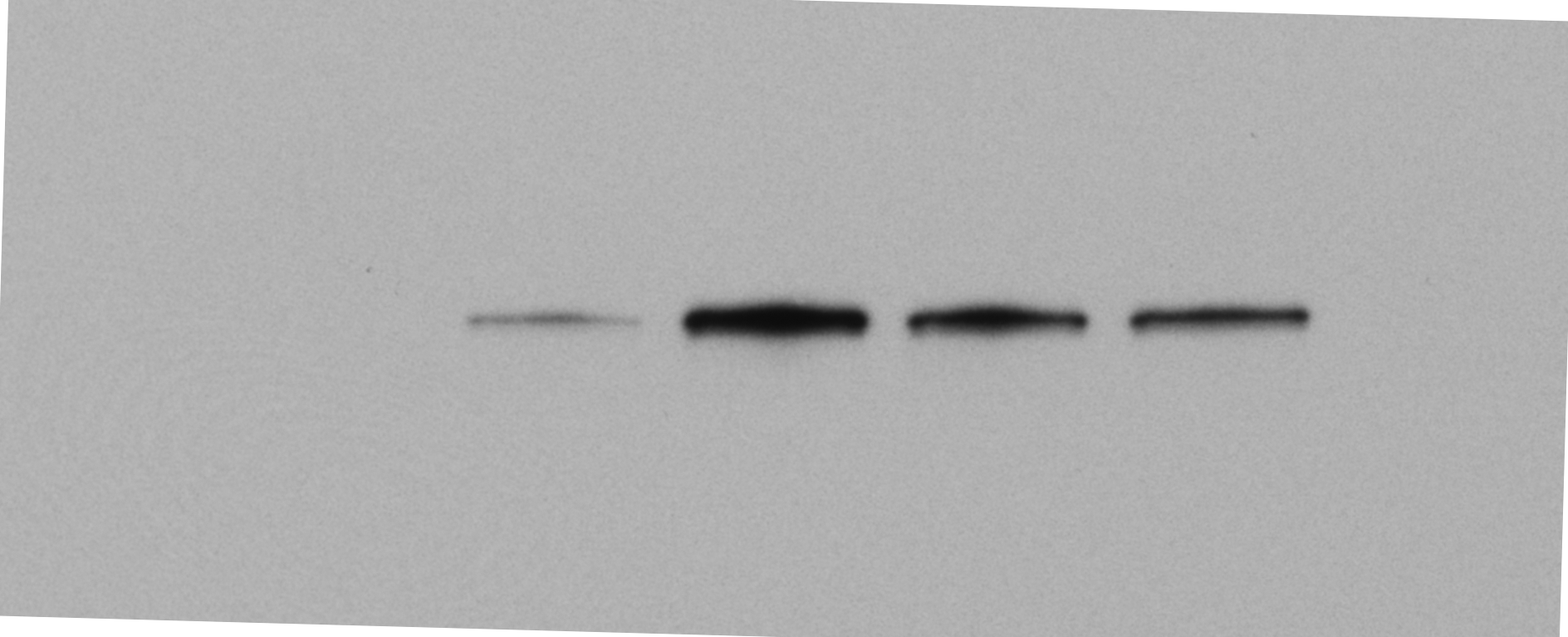

Supplement: Supplementary file 5 — Source data Fig. 2 [file 44319_2024_250_MOESM5_ESM.zip › EMBOR-2024-59387_SourceDataForFigure2/EMBOR-2024-59387_SourceDataForFigure2E/western GR coIP.tif]

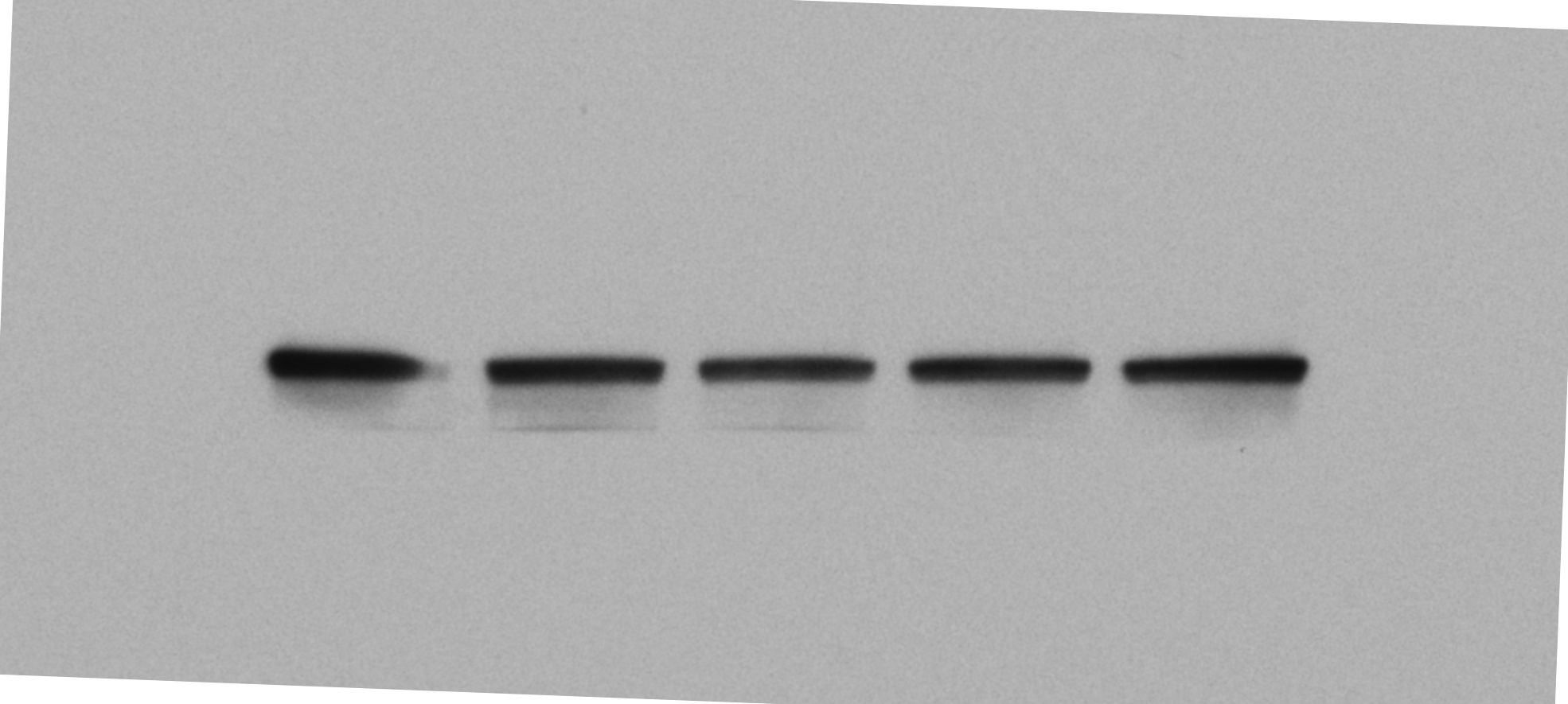

Supplement: Supplementary file 5 — Source data Fig. 2 [file 44319_2024_250_MOESM5_ESM.zip › EMBOR-2024-59387_SourceDataForFigure2/EMBOR-2024-59387_SourceDataForFigure2E/western GR.tif]

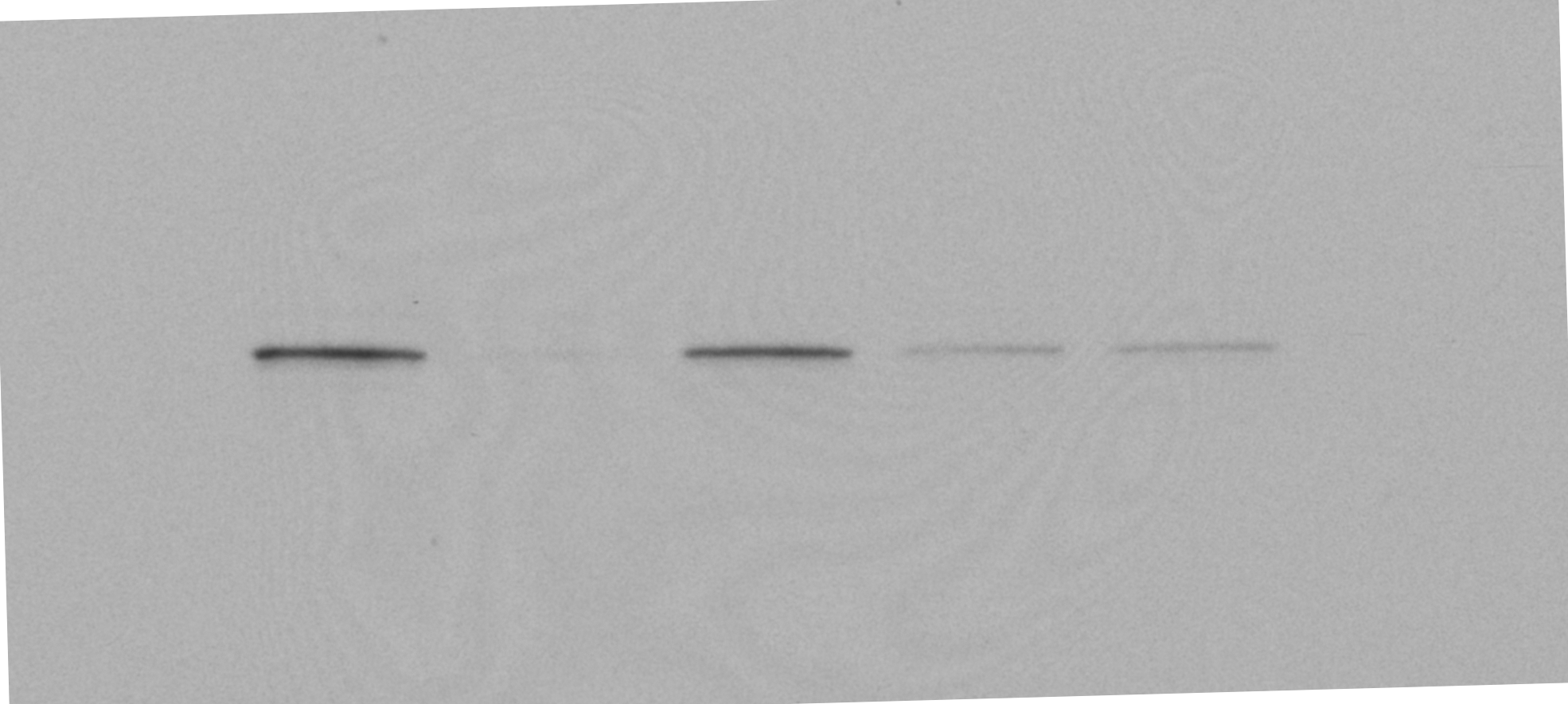

Supplement: Supplementary file 5 — Source data Fig. 2 [file 44319_2024_250_MOESM5_ESM.zip › EMBOR-2024-59387_SourceDataForFigure2/EMBOR-2024-59387_SourceDataForFigure2E/western phos-GR Ser 211.tif]

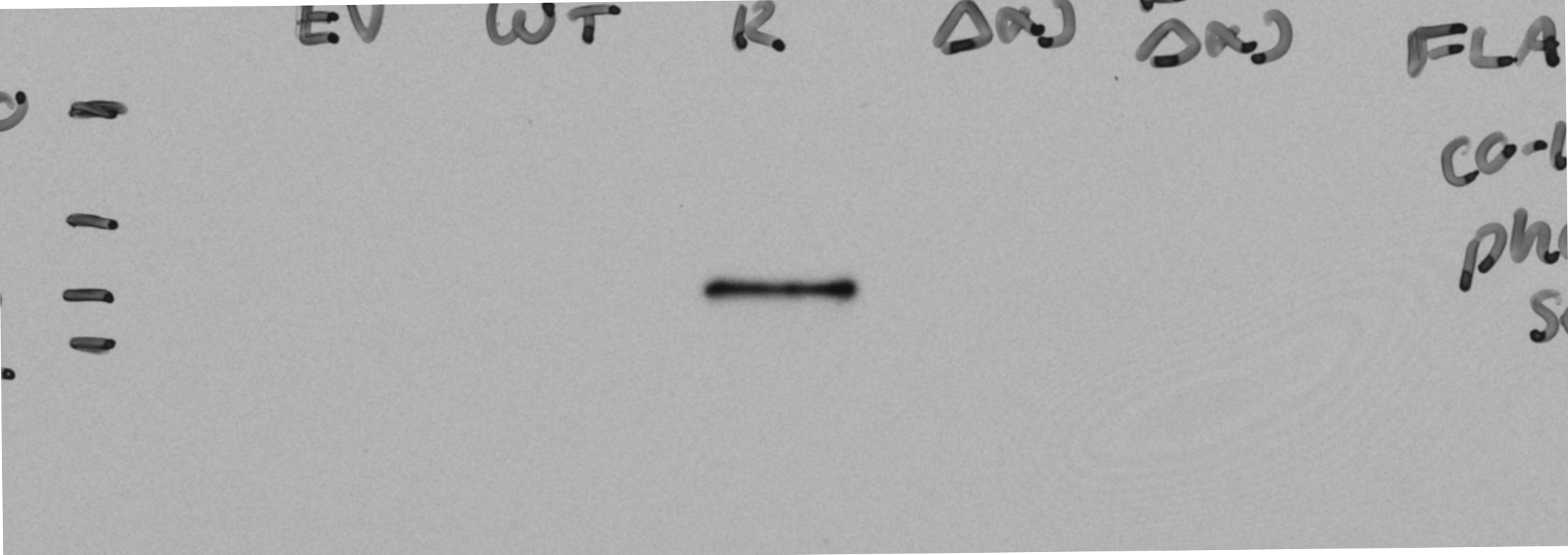

Supplement: Supplementary file 5 — Source data Fig. 2 [file 44319_2024_250_MOESM5_ESM.zip › EMBOR-2024-59387_SourceDataForFigure2/EMBOR-2024-59387_SourceDataForFigure2E/western phos-GR Ser211 coIP.tif]

2E

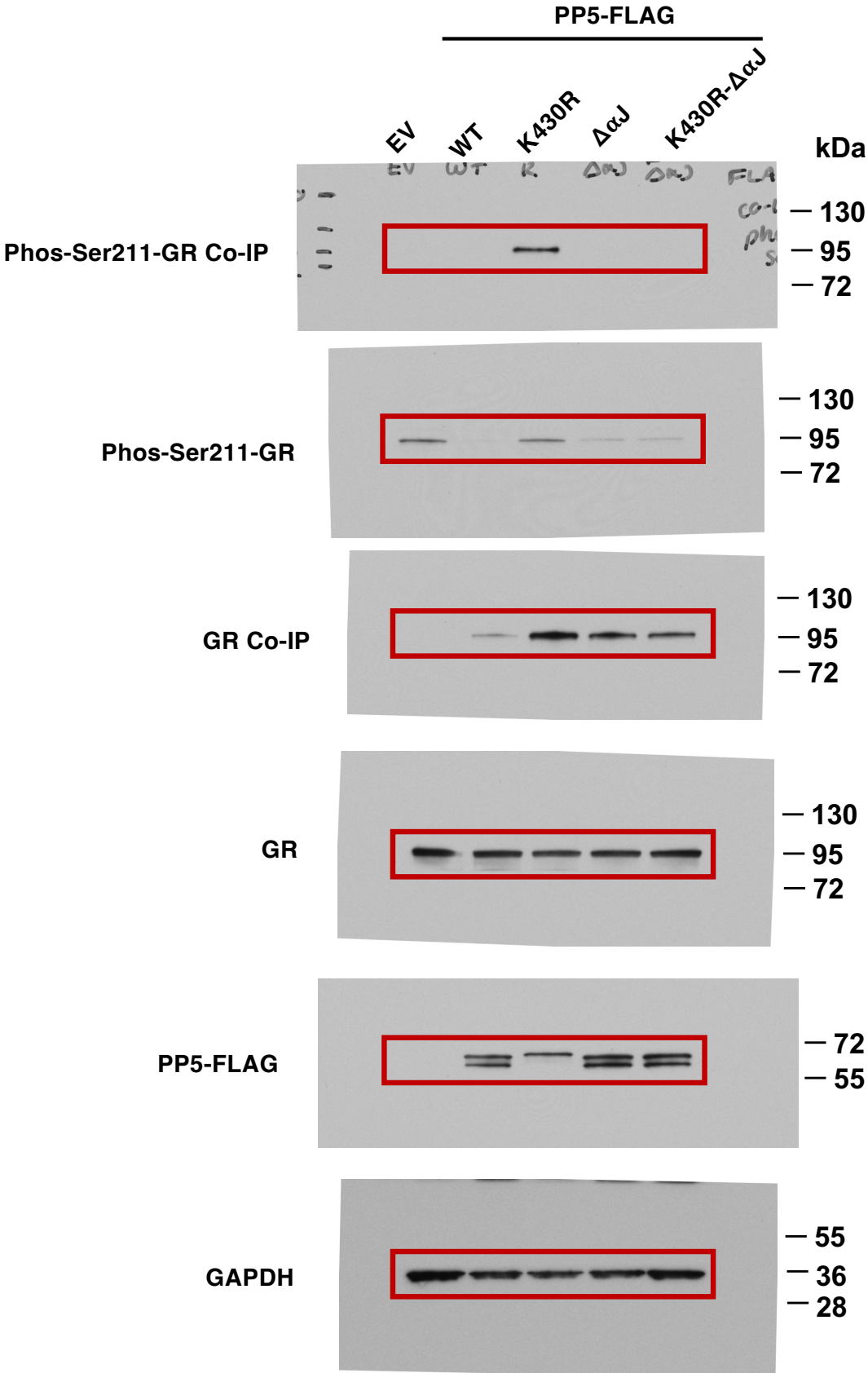

Supplement: Supplementary file 5 — Source data Fig. 2 [file 44319_2024_250_MOESM5_ESM.zip › EMBOR-2024-59387_SourceDataForFigure2/EMBOR-2024-59387_SourceDataForFigure2E/western uncropped annotated.pdf]

2F

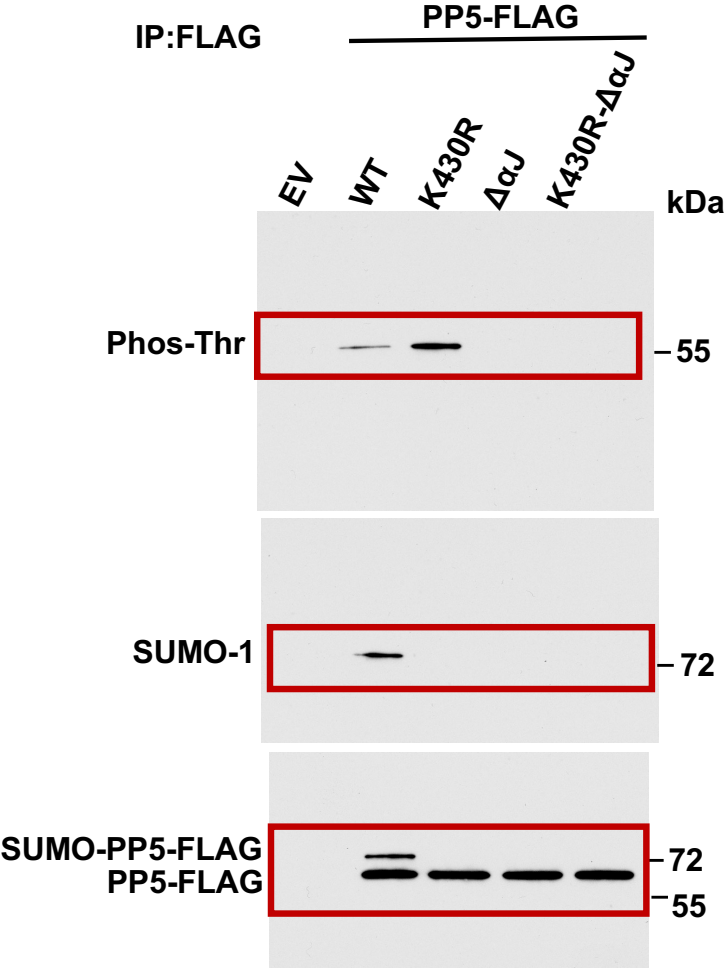

Supplement: Supplementary file 5 — Source data Fig. 2 [file 44319_2024_250_MOESM5_ESM.zip › EMBOR-2024-59387_SourceDataForFigure2/EMBOR-2024-59387_SourceDataForFigure2F/western uncropped annotated.pdf]

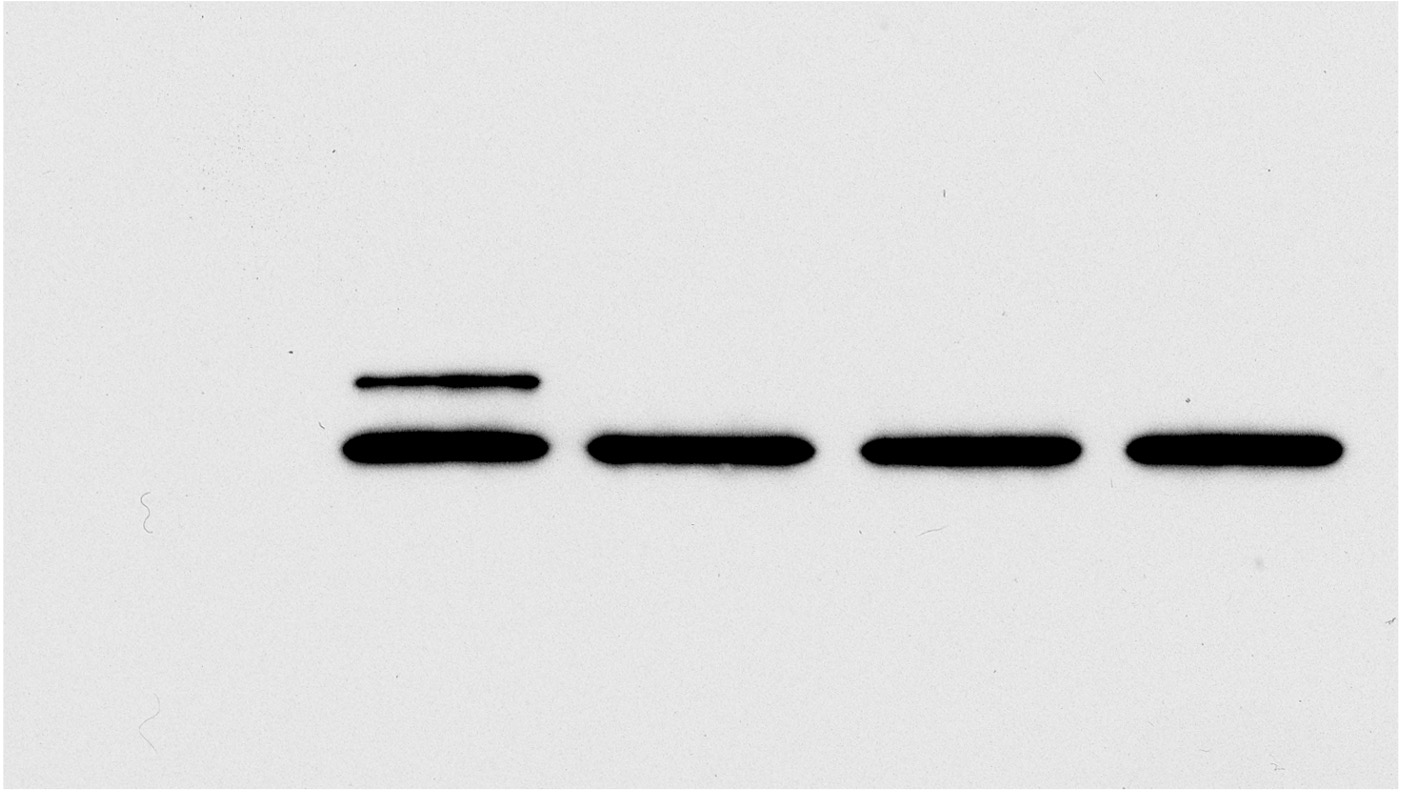

Supplement: Supplementary file 5 — Source data Fig. 2 [file 44319_2024_250_MOESM5_ESM.zip › EMBOR-2024-59387_SourceDataForFigure2/EMBOR-2024-59387_SourceDataForFigure2F/Western-PP5-FLAG-IP.jpg]

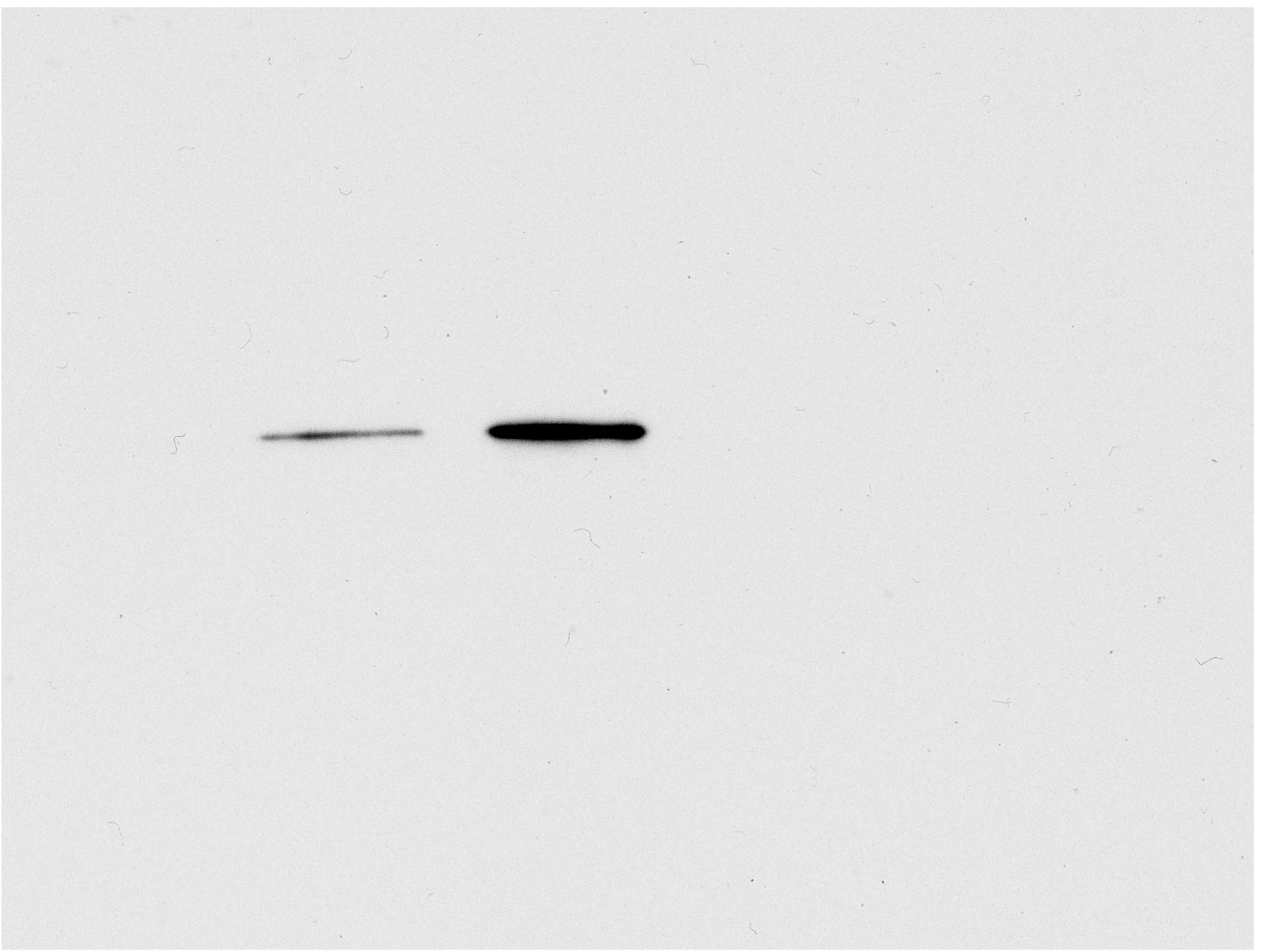

Supplement: Supplementary file 5 — Source data Fig. 2 [file 44319_2024_250_MOESM5_ESM.zip › EMBOR-2024-59387_SourceDataForFigure2/EMBOR-2024-59387_SourceDataForFigure2F/Western-PP5-Phos-Thr.jpg]

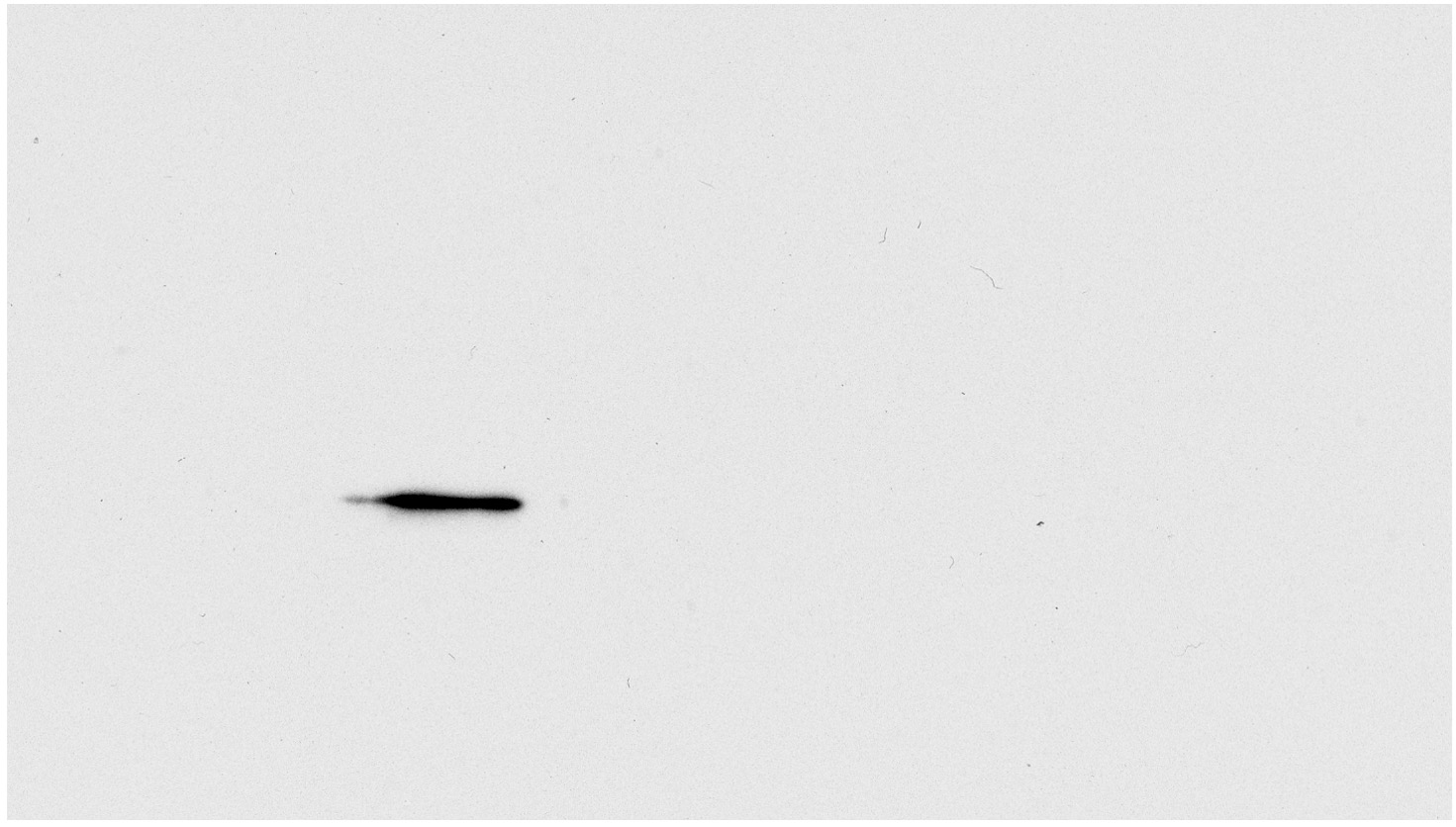

Supplement: Supplementary file 5 — Source data Fig. 2 [file 44319_2024_250_MOESM5_ESM.zip › EMBOR-2024-59387_SourceDataForFigure2/EMBOR-2024-59387_SourceDataForFigure2F/Western-PP5-SUMO1.jpg]

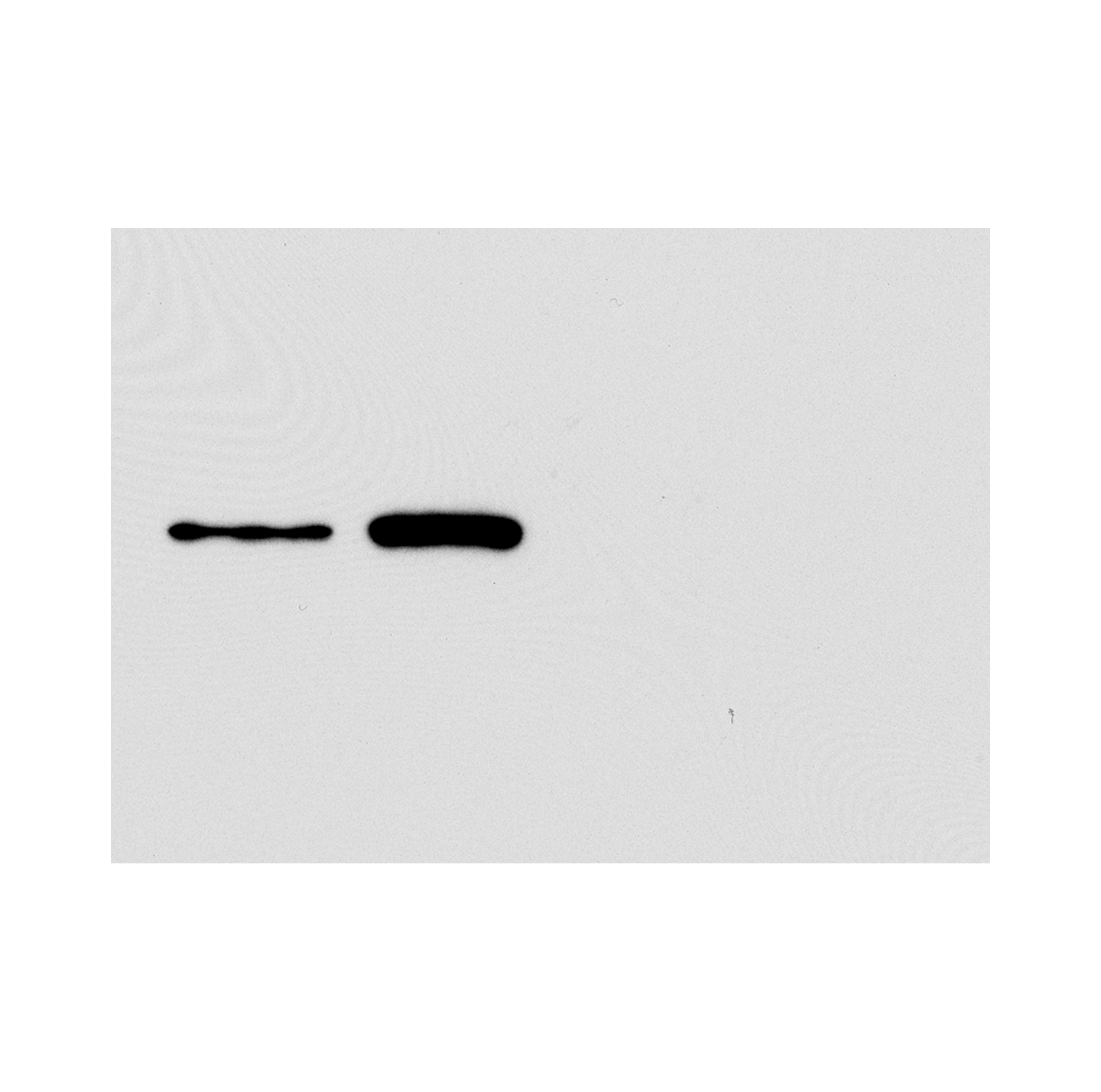

Supplement: Supplementary file 6 — Source data Fig. 3 [file 44319_2024_250_MOESM6_ESM.zip › EMBOR-2024-59387_SourceDataForFigure3/EMBOR-2024-59387_SourceDataForFigure3B/Western PP5-PhosThr-IP.tif]

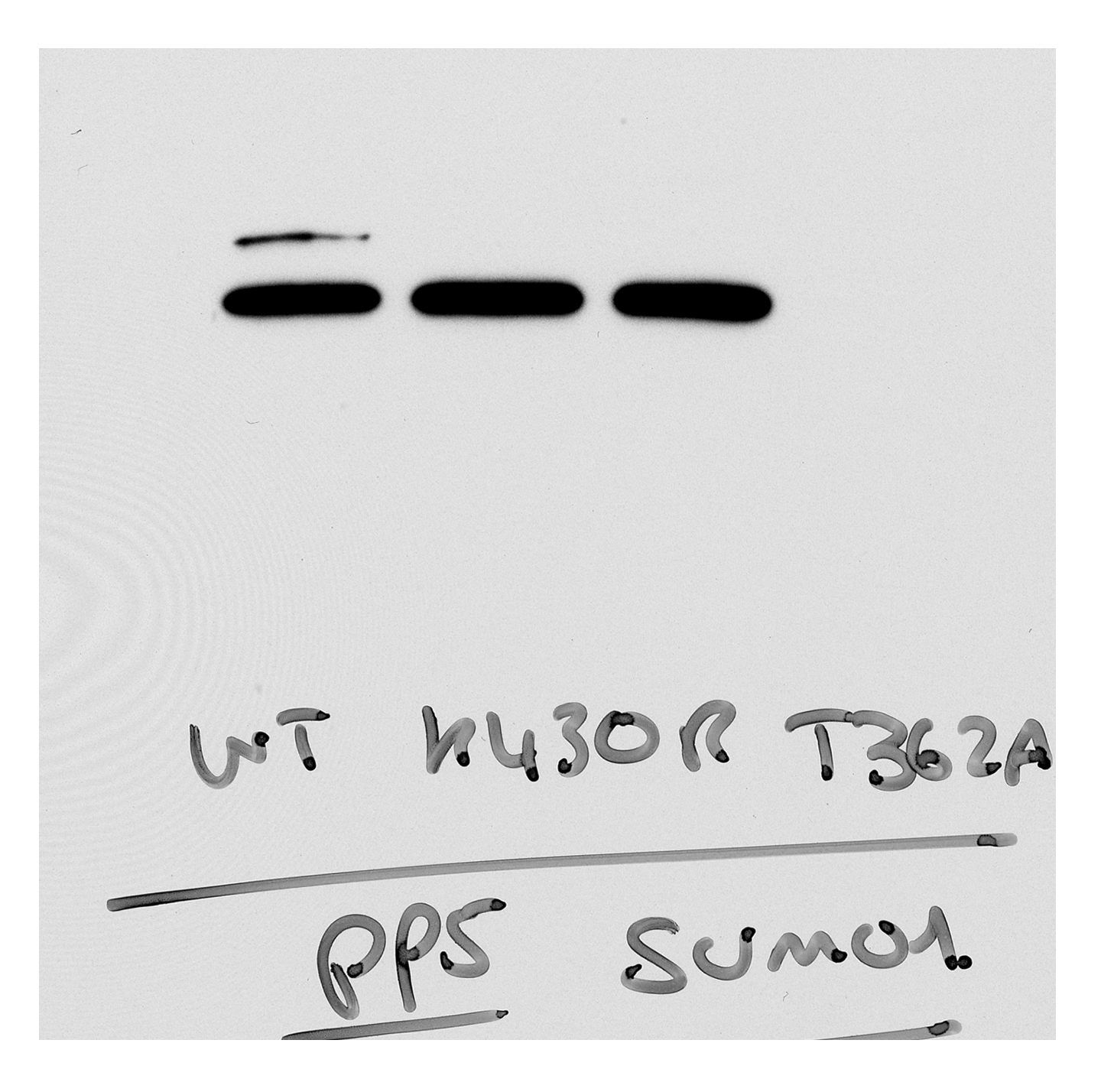

Supplement: Supplementary file 6 — Source data Fig. 3 [file 44319_2024_250_MOESM6_ESM.zip › EMBOR-2024-59387_SourceDataForFigure3/EMBOR-2024-59387_SourceDataForFigure3B/Western PP5FLAG-IP.tif]

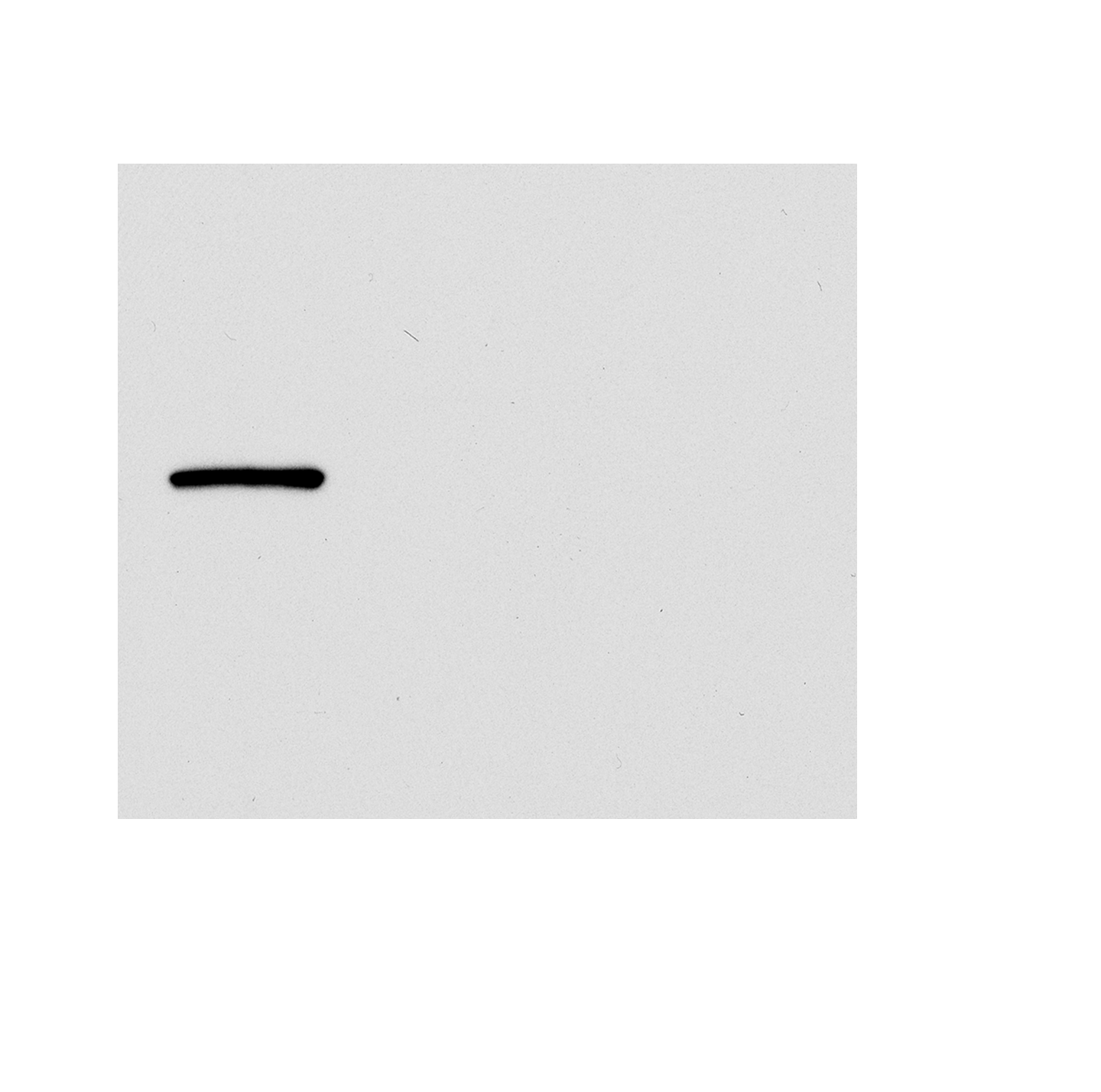

Supplement: Supplementary file 6 — Source data Fig. 3 [file 44319_2024_250_MOESM6_ESM.zip › EMBOR-2024-59387_SourceDataForFigure3/EMBOR-2024-59387_SourceDataForFigure3B/Western PP5SUMO1-IP.tif]

3B

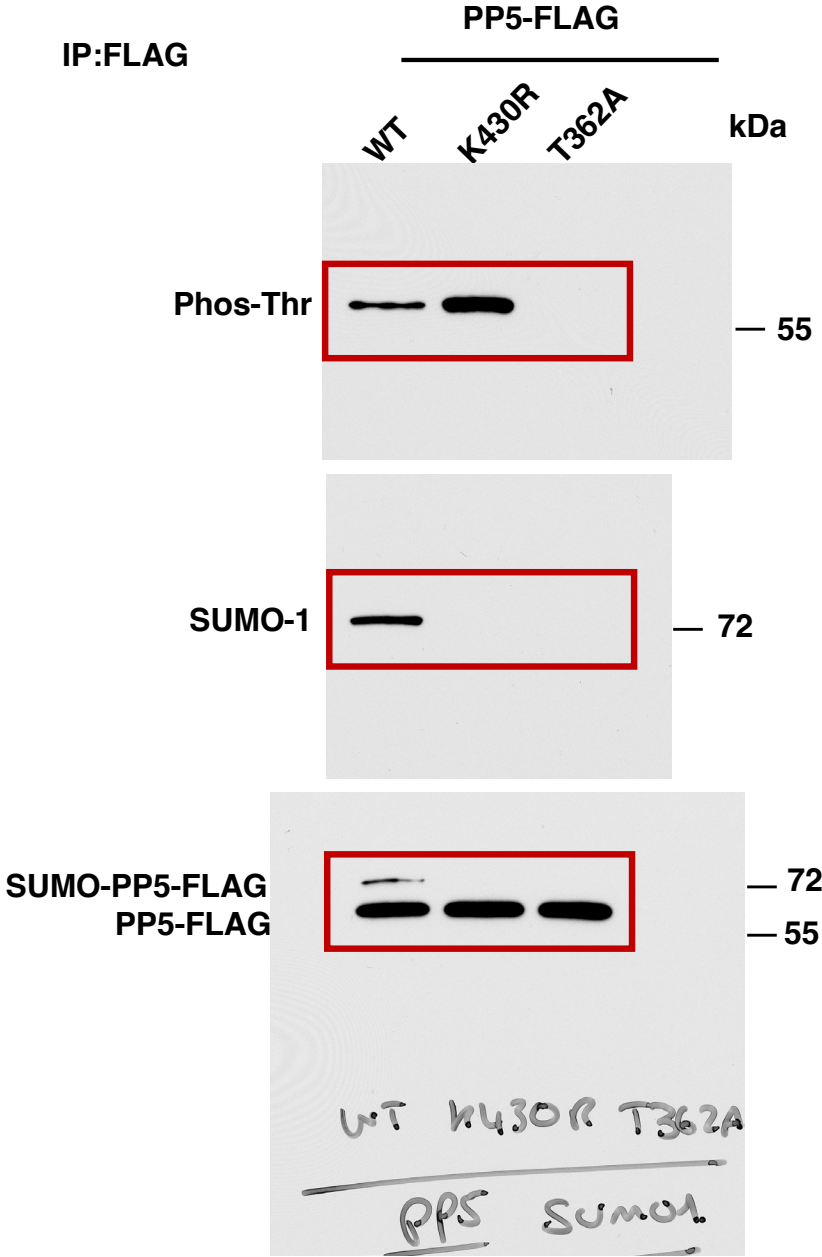

Supplement: Supplementary file 6 — Source data Fig. 3 [file 44319_2024_250_MOESM6_ESM.zip › EMBOR-2024-59387_SourceDataForFigure3/EMBOR-2024-59387_SourceDataForFigure3B/western uncropped annotated.pdf]

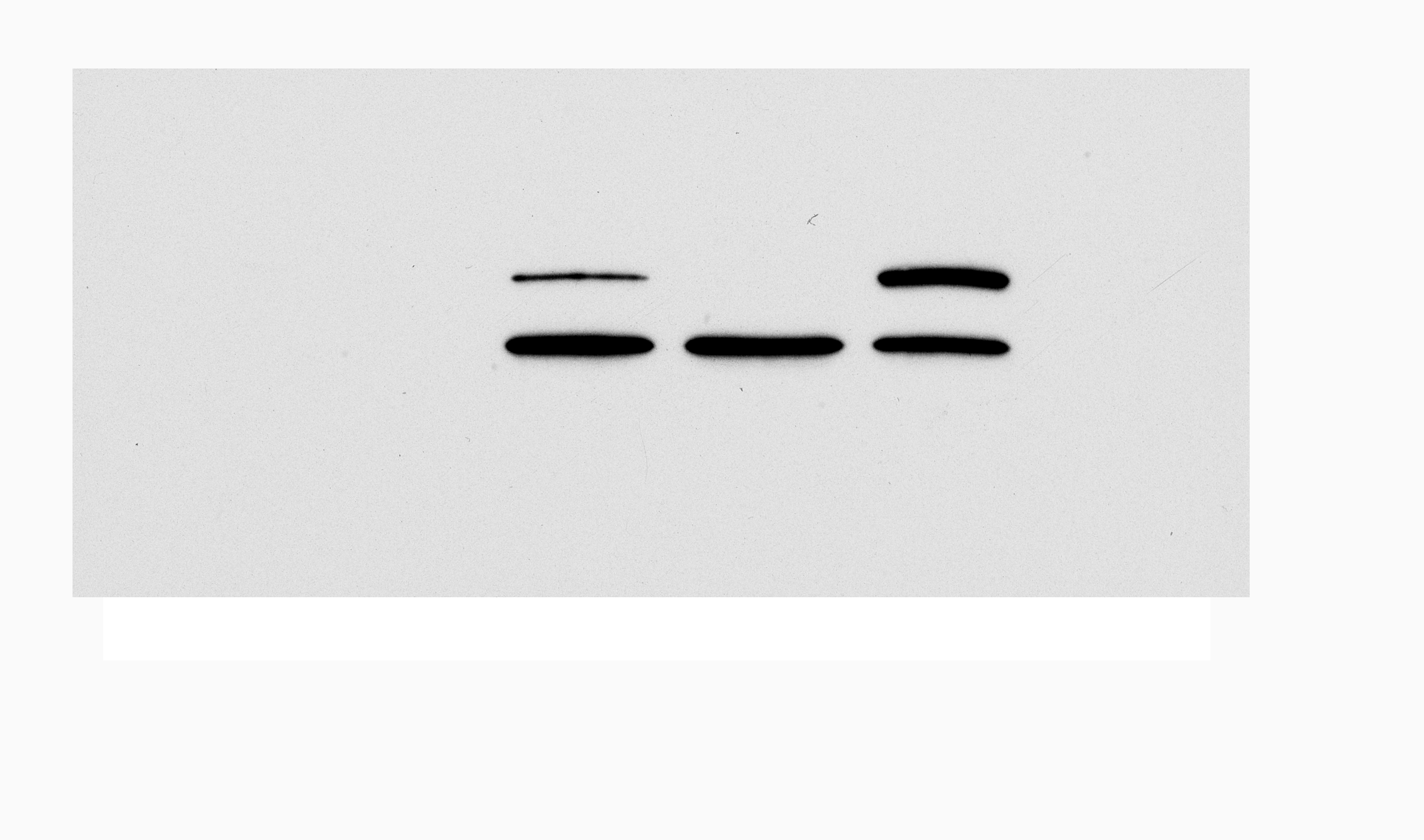

Supplement: Supplementary file 6 — Source data Fig. 3 [file 44319_2024_250_MOESM6_ESM.zip › EMBOR-2024-59387_SourceDataForFigure3/EMBOR-2024-59387_SourceDataForFigure3C/Western PP5-FLAG-IP.tif]

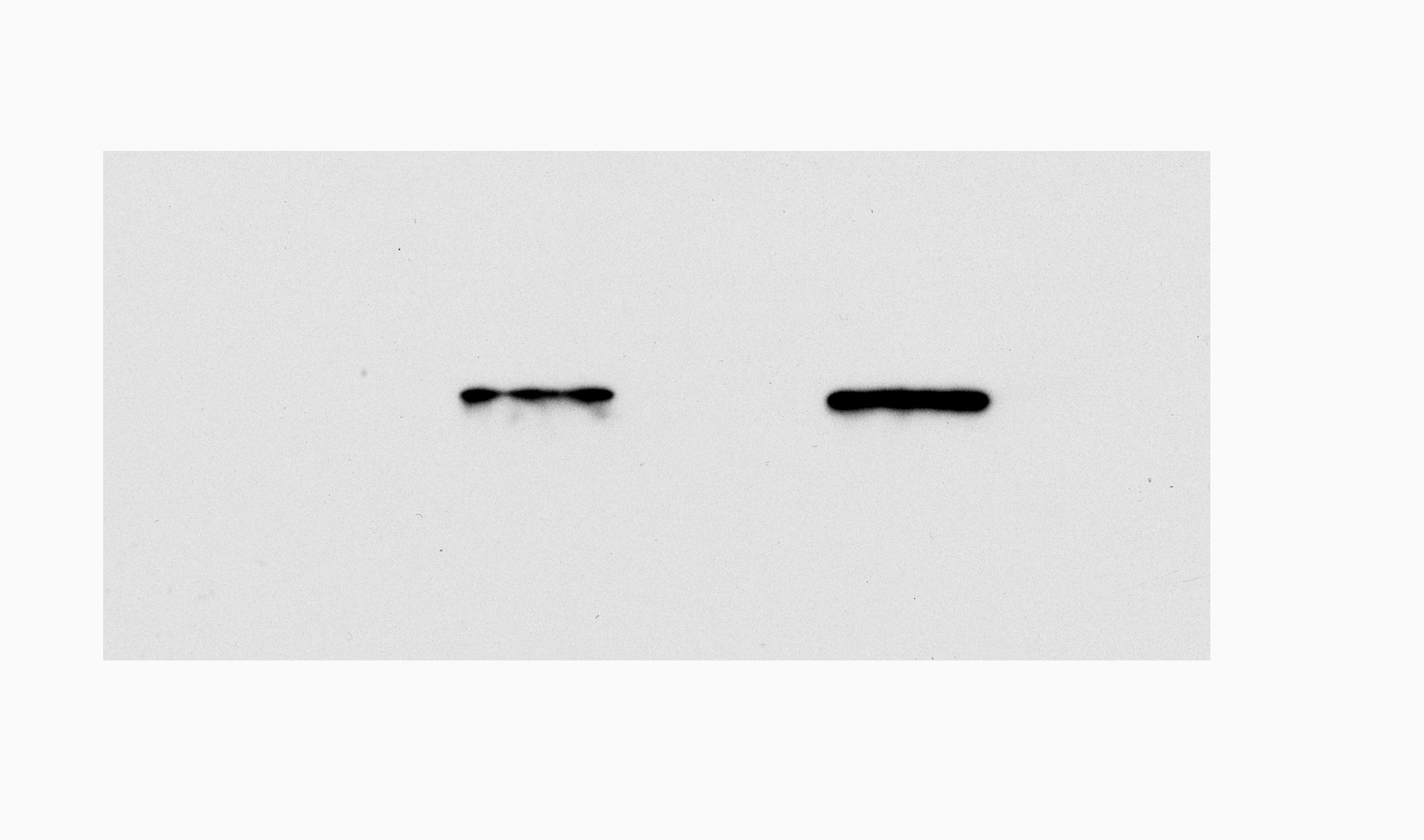

Supplement: Supplementary file 6 — Source data Fig. 3 [file 44319_2024_250_MOESM6_ESM.zip › EMBOR-2024-59387_SourceDataForFigure3/EMBOR-2024-59387_SourceDataForFigure3C/Western PP5-SUMO1.tif]

3C

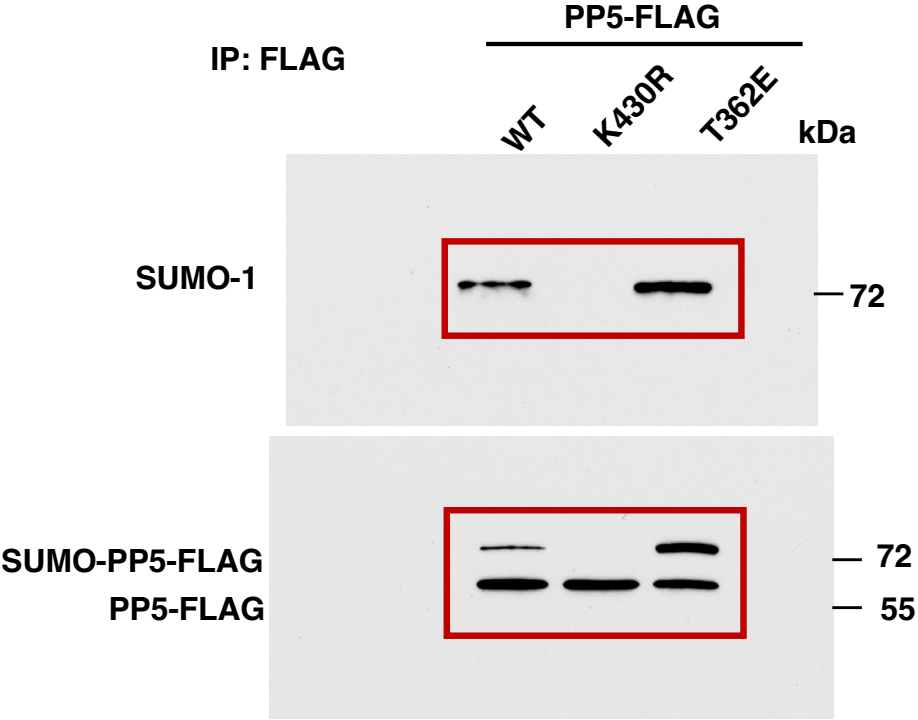

Supplement: Supplementary file 6 — Source data Fig. 3 [file 44319_2024_250_MOESM6_ESM.zip › EMBOR-2024-59387_SourceDataForFigure3/EMBOR-2024-59387_SourceDataForFigure3C/western uncropped annotated.pdf]

IP: FLAG

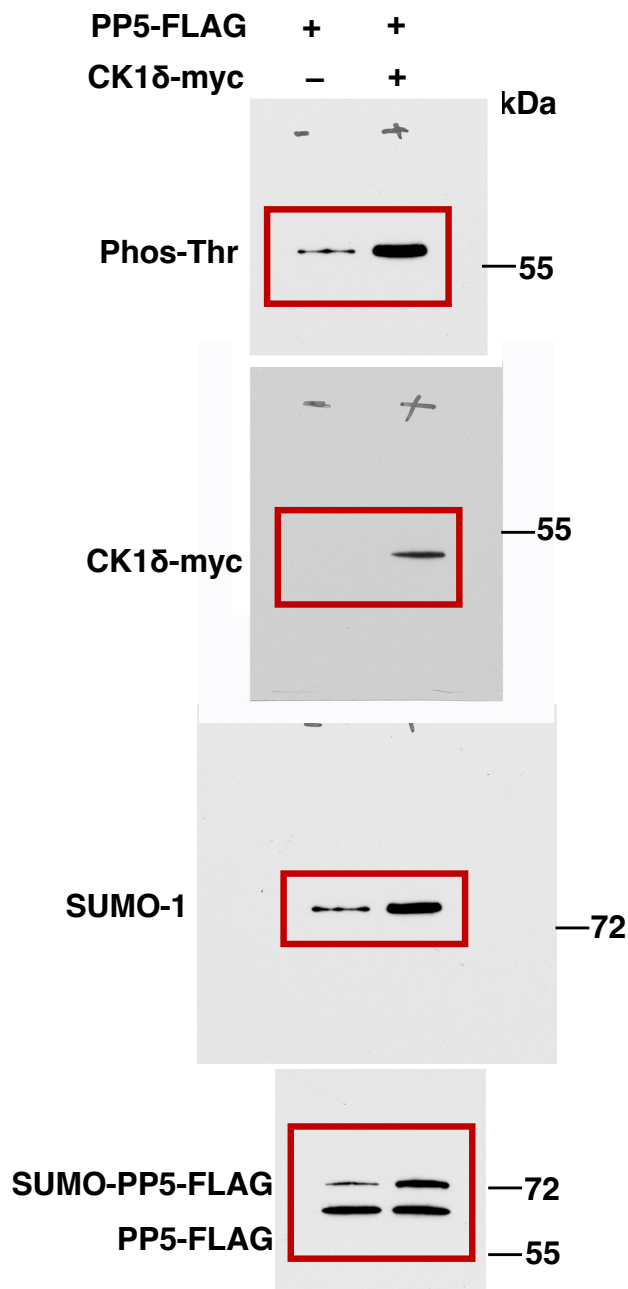

Supplement: Supplementary file 6 — Source data Fig. 3 [file 44319_2024_250_MOESM6_ESM.zip › EMBOR-2024-59387_SourceDataForFigure3/EMBOR-2024-59387_SourceDataForFigure3D/western uncropped annotated.pdf]

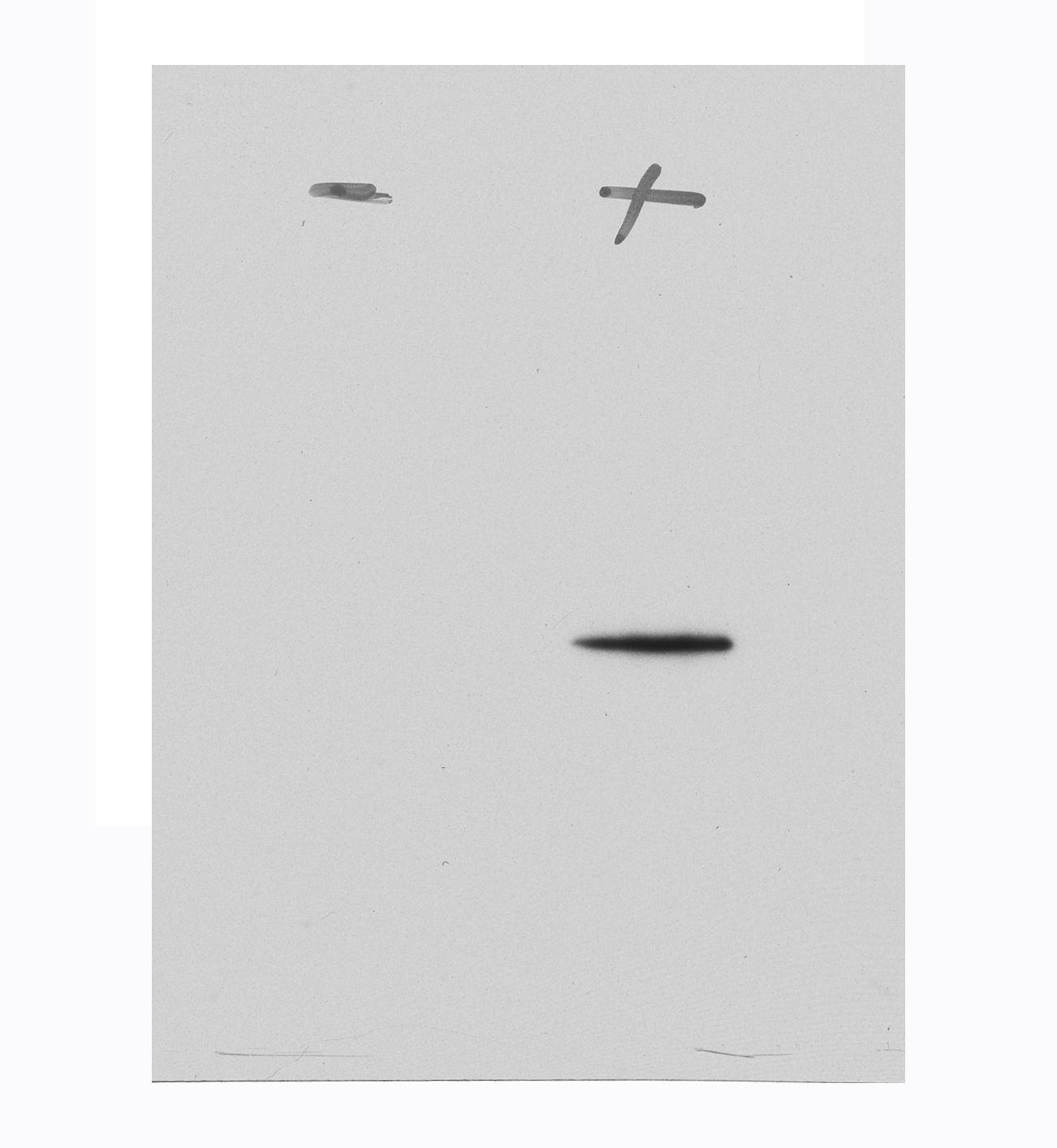

Supplement: Supplementary file 6 — Source data Fig. 3 [file 44319_2024_250_MOESM6_ESM.zip › EMBOR-2024-59387_SourceDataForFigure3/EMBOR-2024-59387_SourceDataForFigure3D/Western-CK1d-cmyc.tif]

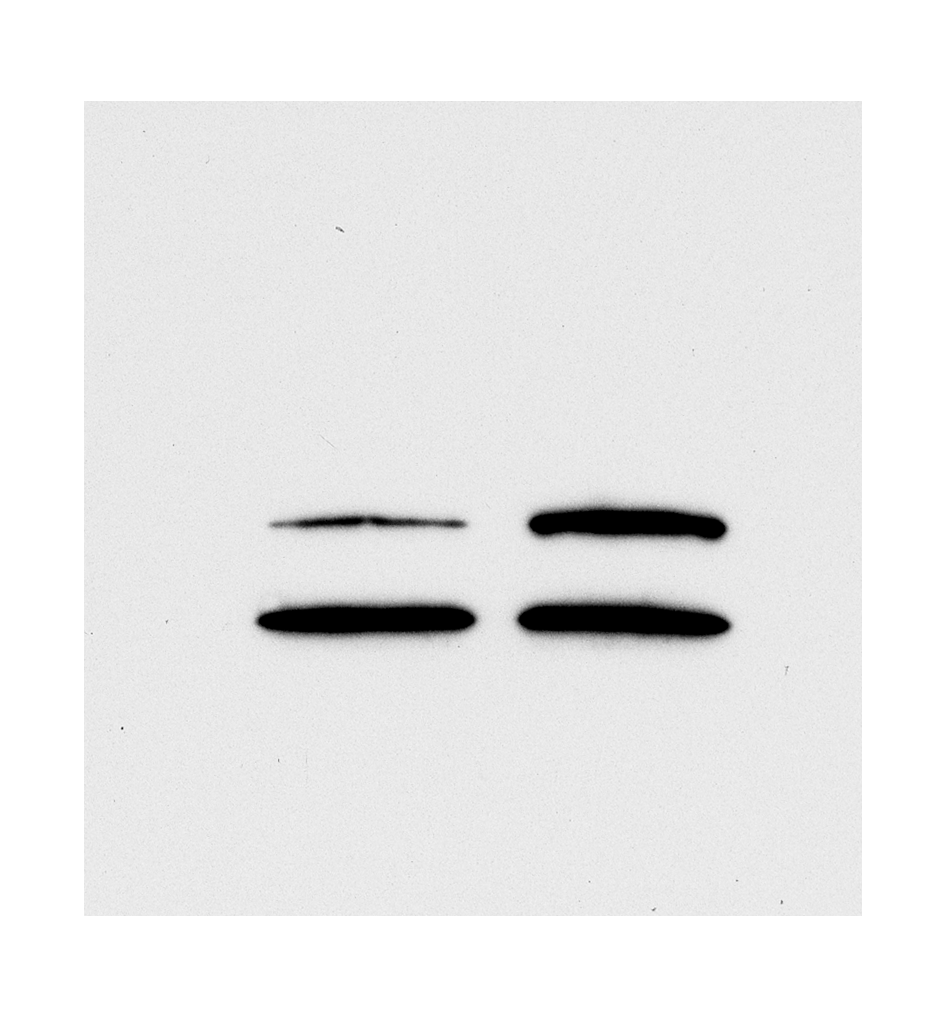

Supplement: Supplementary file 6 — Source data Fig. 3 [file 44319_2024_250_MOESM6_ESM.zip › EMBOR-2024-59387_SourceDataForFigure3/EMBOR-2024-59387_SourceDataForFigure3D/Western-PP5-FLAG-IP.tif]

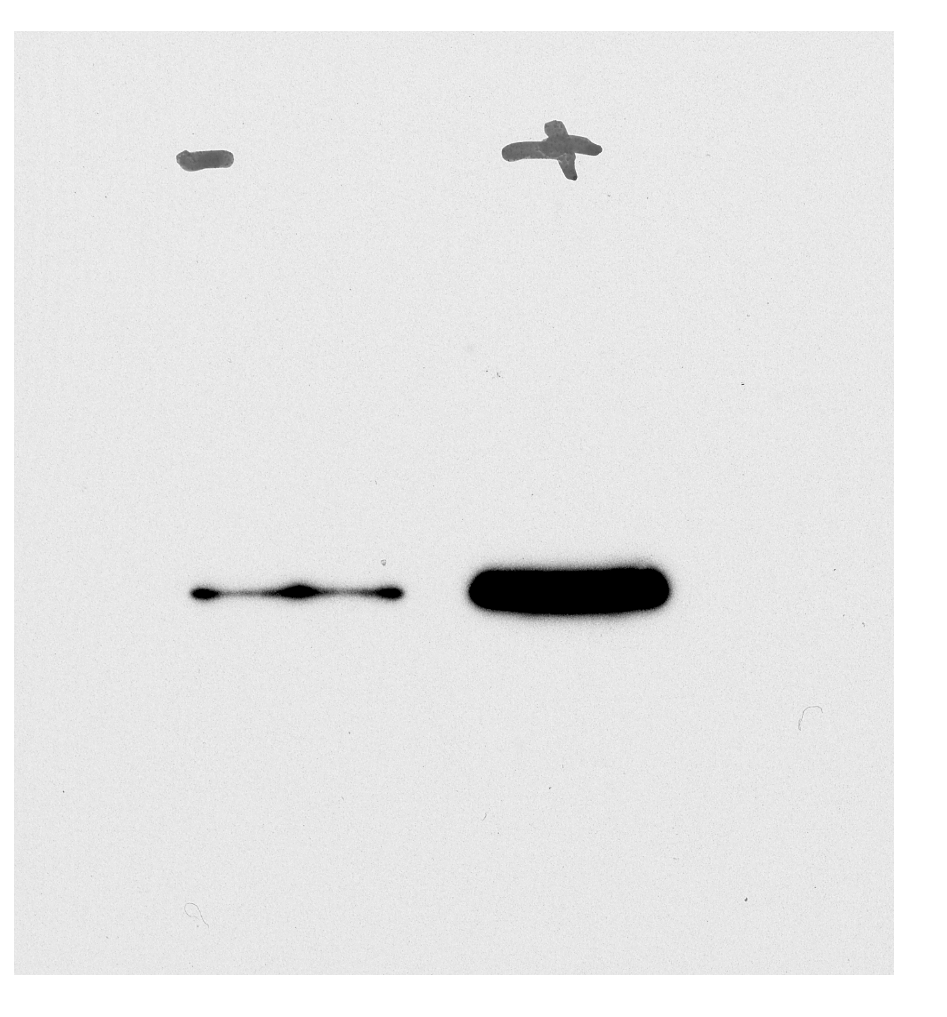

Supplement: Supplementary file 6 — Source data Fig. 3 [file 44319_2024_250_MOESM6_ESM.zip › EMBOR-2024-59387_SourceDataForFigure3/EMBOR-2024-59387_SourceDataForFigure3D/Western-PP5-Phos-Thr.tif]

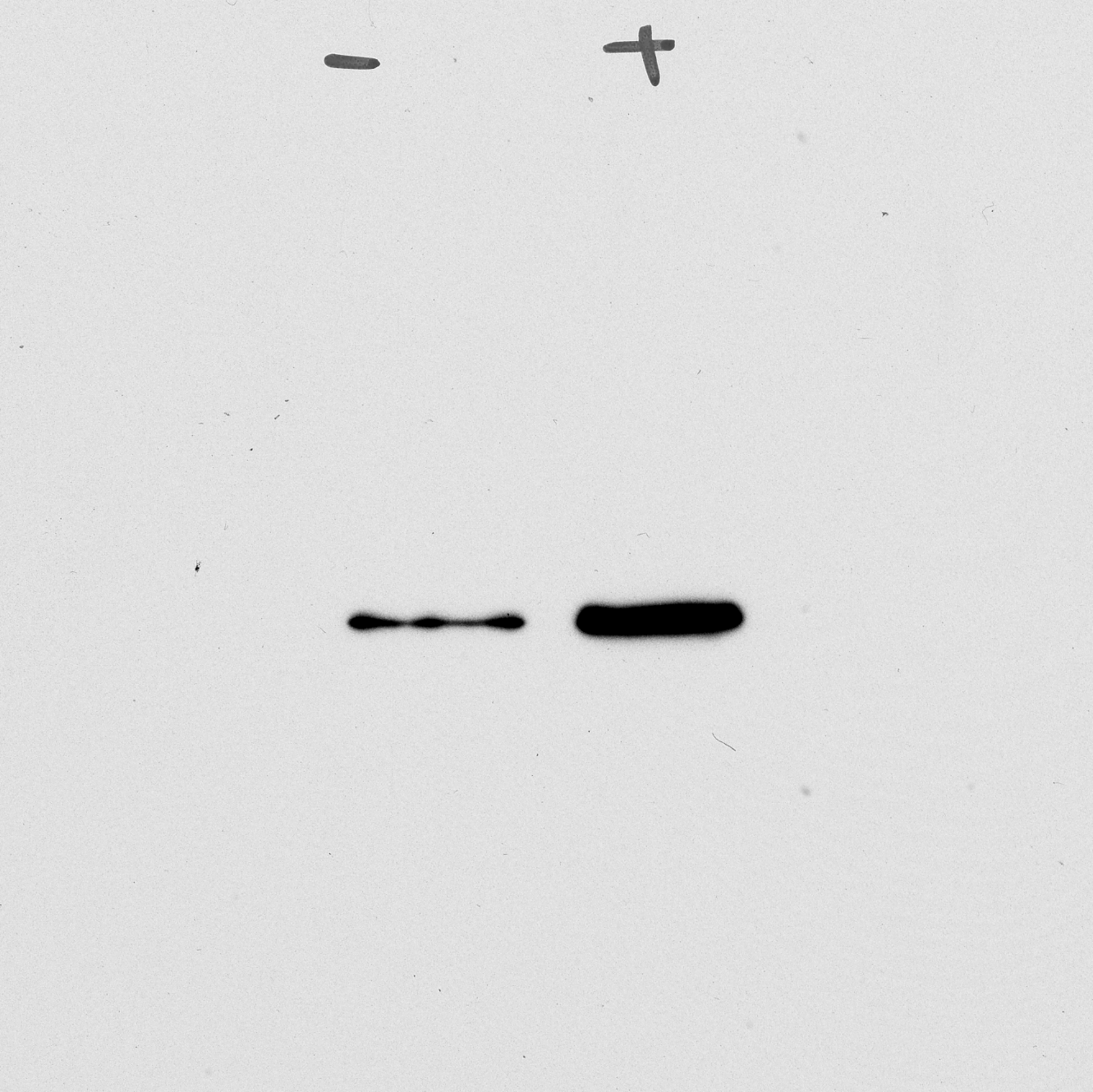

Supplement: Supplementary file 6 — Source data Fig. 3 [file 44319_2024_250_MOESM6_ESM.zip › EMBOR-2024-59387_SourceDataForFigure3/EMBOR-2024-59387_SourceDataForFigure3D/Western-PP5-SUMO1.tif]

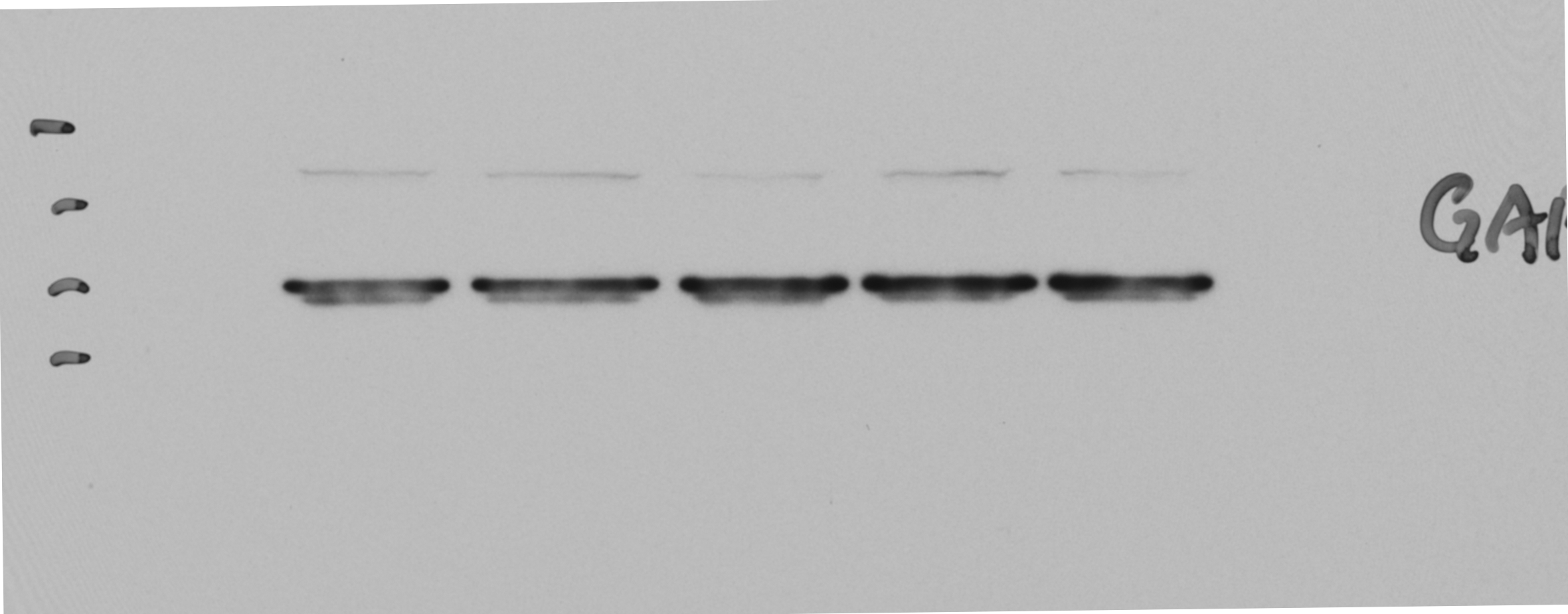

Supplement: Supplementary file 6 — Source data Fig. 3 [file 44319_2024_250_MOESM6_ESM.zip › EMBOR-2024-59387_SourceDataForFigure3/EMBOR-2024-59387_SourceDataForFigure3E/western GAPDH.tif]

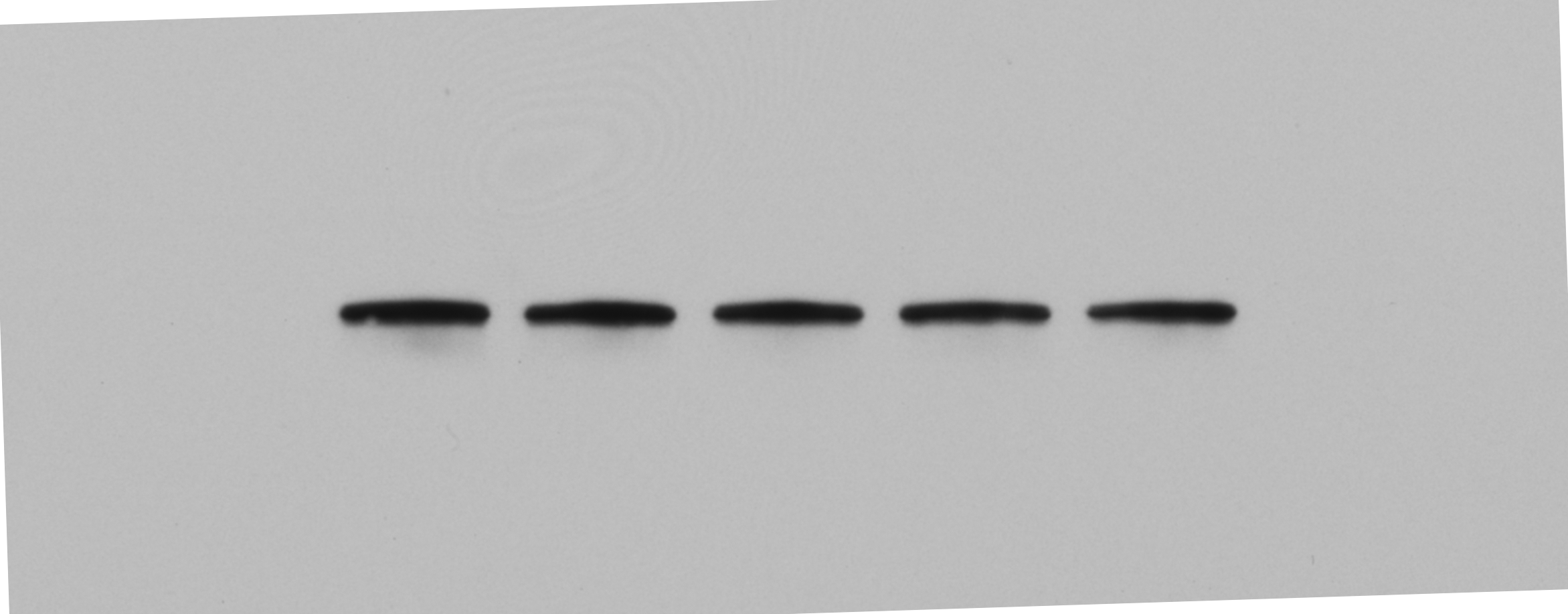

Supplement: Supplementary file 6 — Source data Fig. 3 [file 44319_2024_250_MOESM6_ESM.zip › EMBOR-2024-59387_SourceDataForFigure3/EMBOR-2024-59387_SourceDataForFigure3E/western GR.tif]

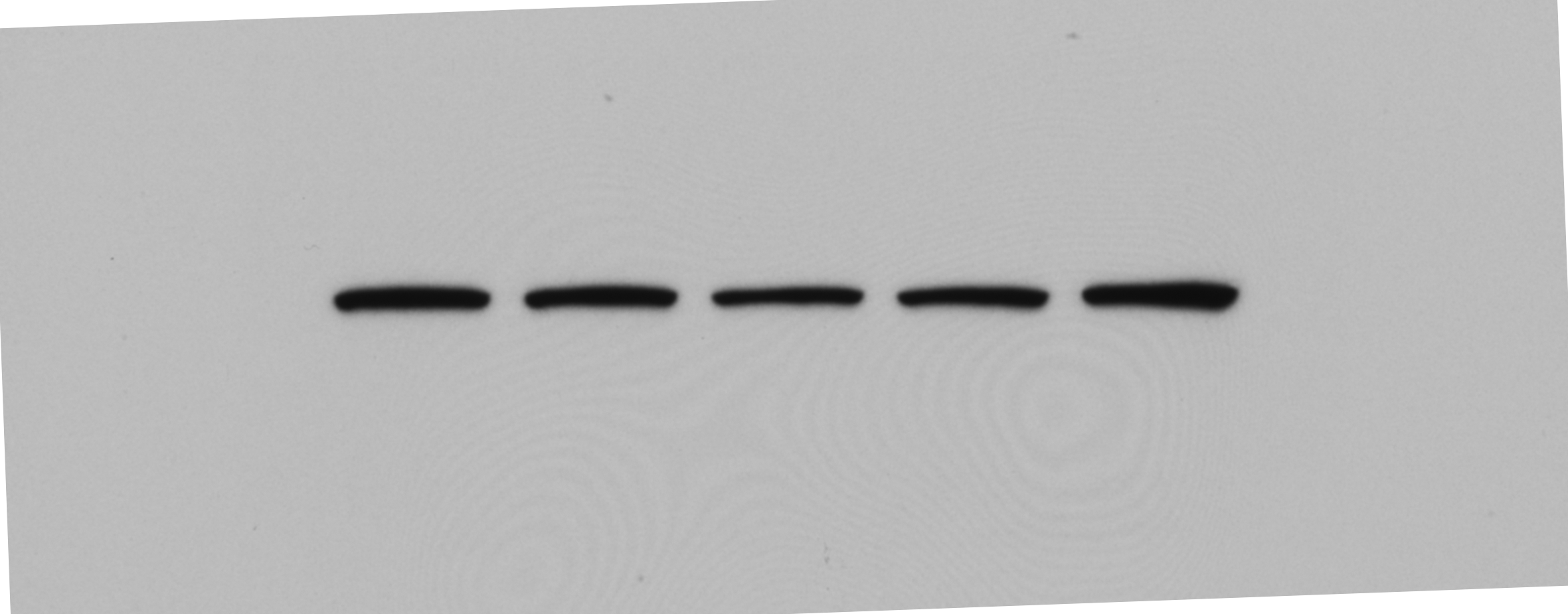

Supplement: Supplementary file 6 — Source data Fig. 3 [file 44319_2024_250_MOESM6_ESM.zip › EMBOR-2024-59387_SourceDataForFigure3/EMBOR-2024-59387_SourceDataForFigure3E/western Hsp90.tif]

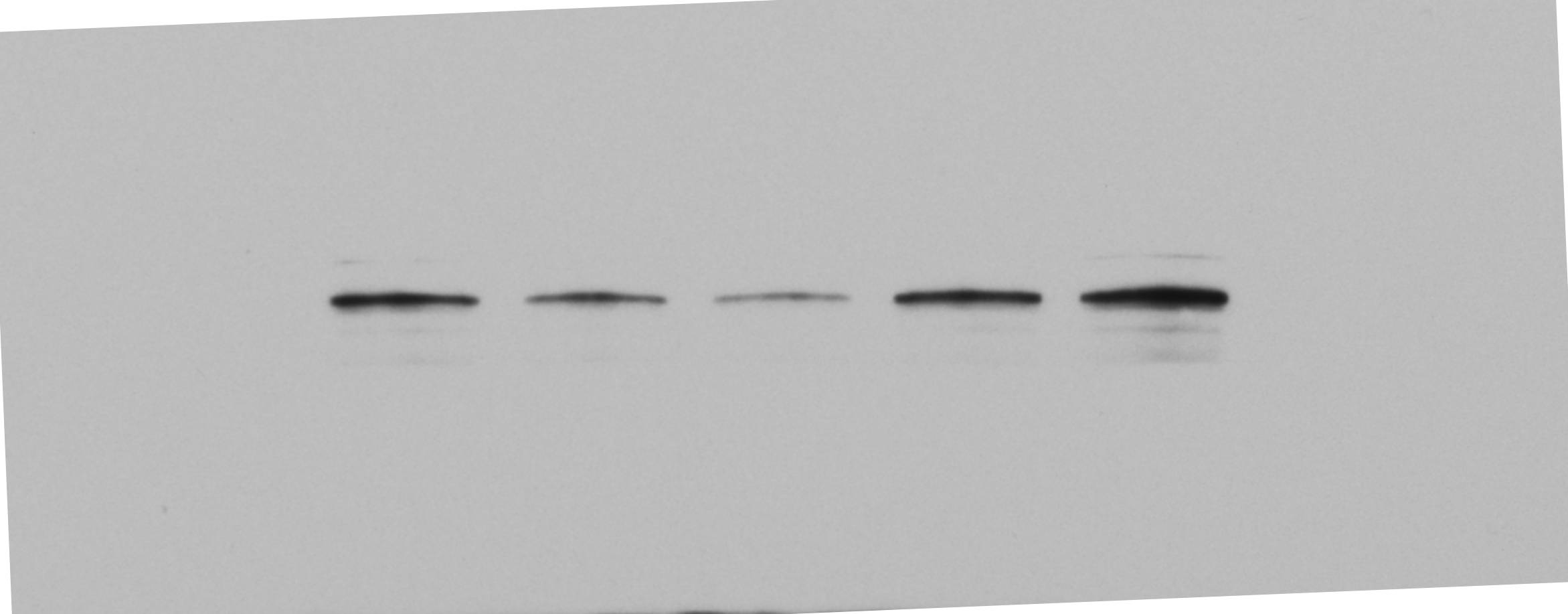

Supplement: Supplementary file 6 — Source data Fig. 3 [file 44319_2024_250_MOESM6_ESM.zip › EMBOR-2024-59387_SourceDataForFigure3/EMBOR-2024-59387_SourceDataForFigure3E/western phosGR S211.tif]

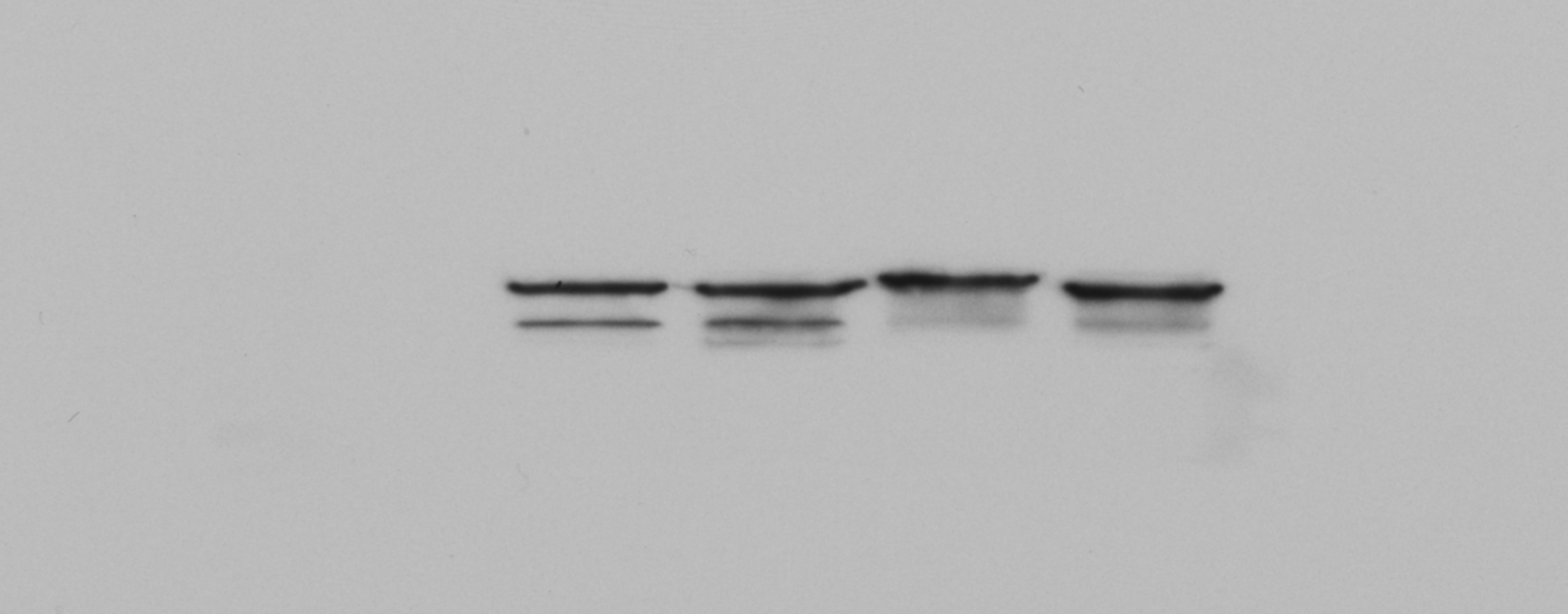

Supplement: Supplementary file 6 — Source data Fig. 3 [file 44319_2024_250_MOESM6_ESM.zip › EMBOR-2024-59387_SourceDataForFigure3/EMBOR-2024-59387_SourceDataForFigure3E/western PP5-FLAG.tif]

3E

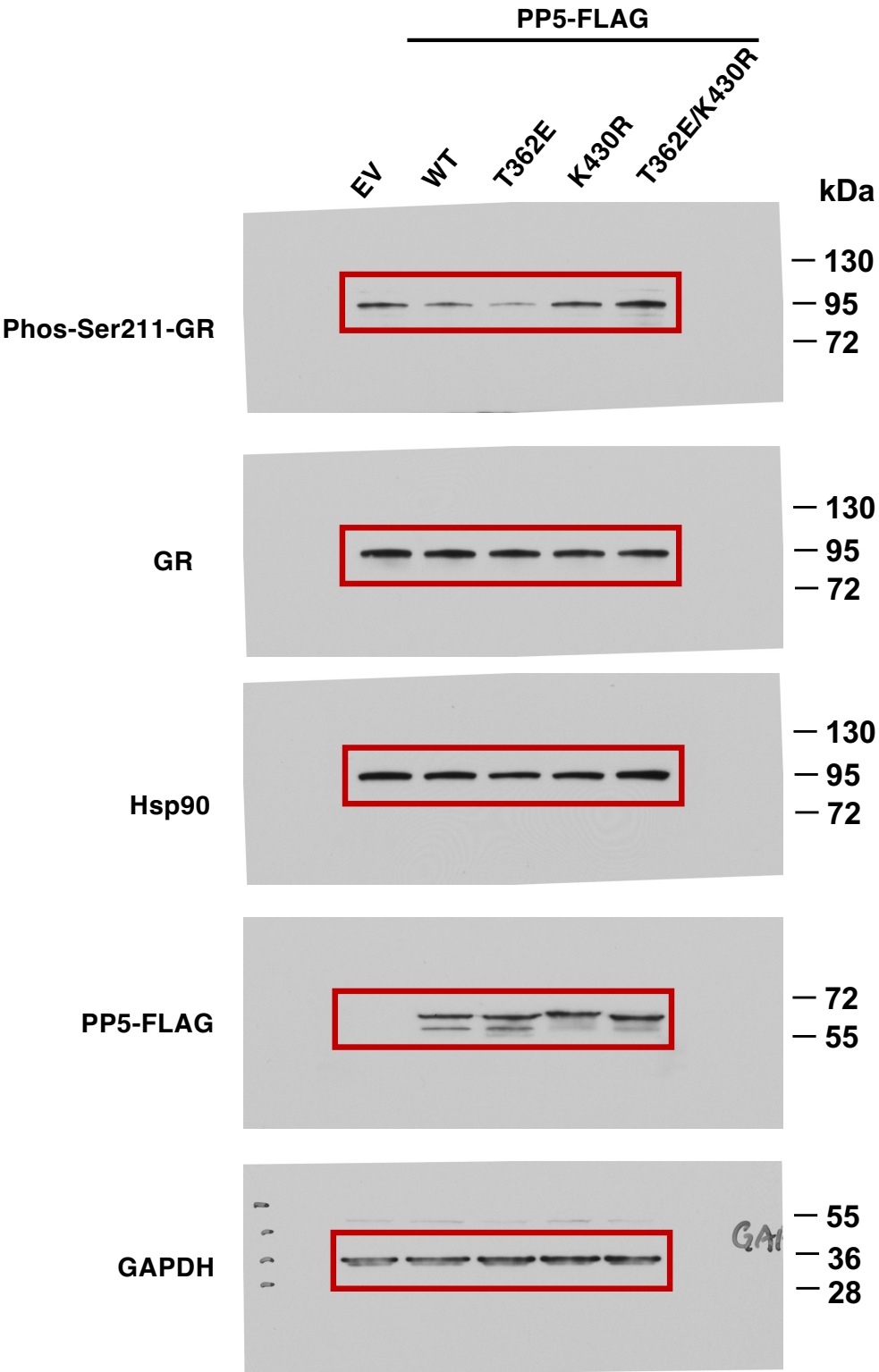

Supplement: Supplementary file 6 — Source data Fig. 3 [file 44319_2024_250_MOESM6_ESM.zip › EMBOR-2024-59387_SourceDataForFigure3/EMBOR-2024-59387_SourceDataForFigure3E/western uncropped annotated.pdf]

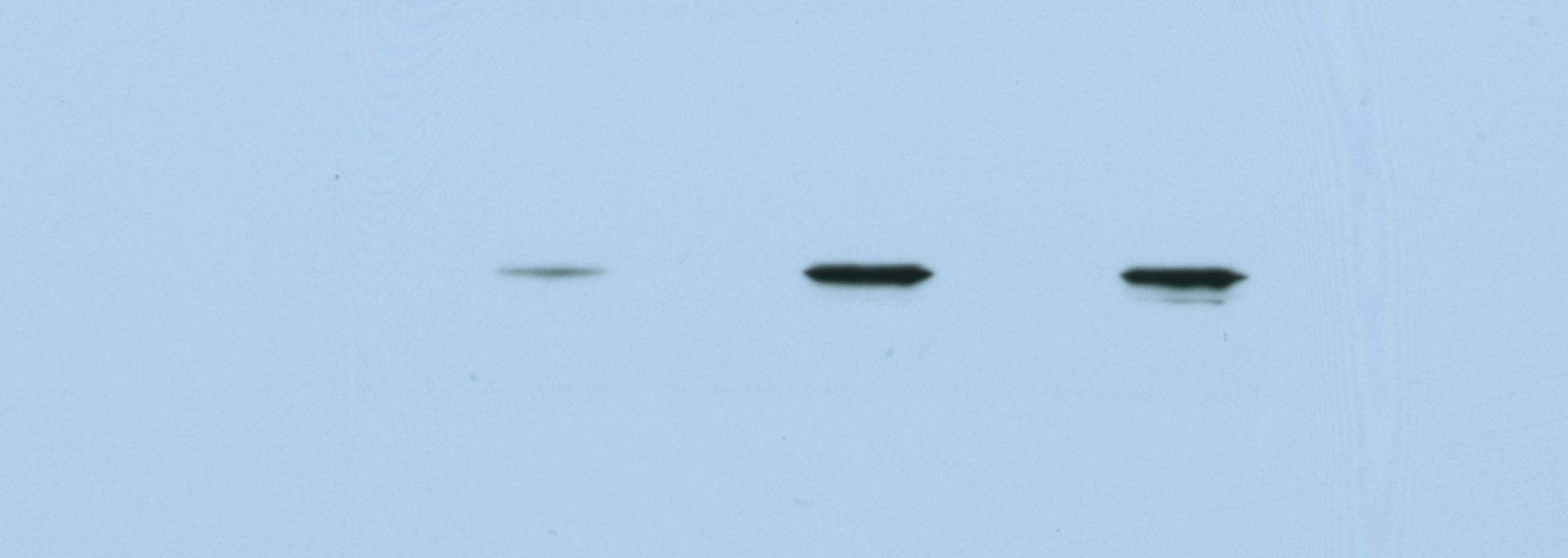

Supplement: Supplementary file 6 — Source data Fig. 3 [file 44319_2024_250_MOESM6_ESM.zip › EMBOR-2024-59387_SourceDataForFigure3/EMBOR-2024-59387_SourceDataForFigure3F/3F Replicate 1/western CK1-myc.tif]

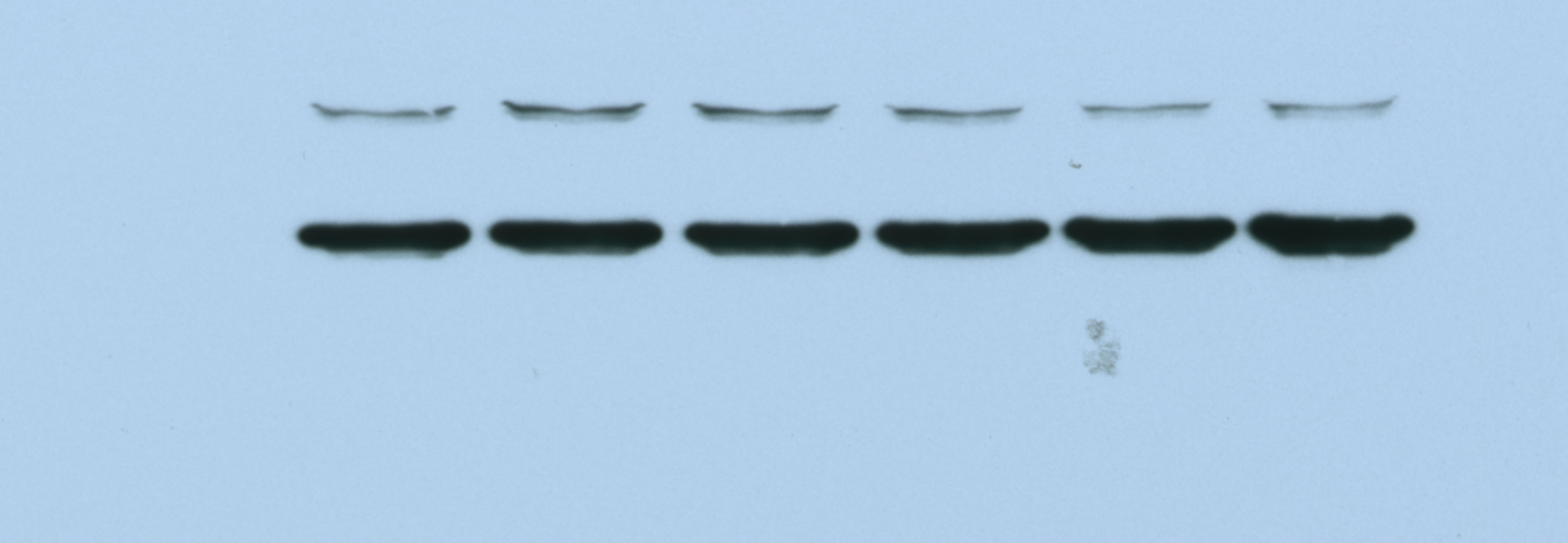

Supplement: Supplementary file 6 — Source data Fig. 3 [file 44319_2024_250_MOESM6_ESM.zip › EMBOR-2024-59387_SourceDataForFigure3/EMBOR-2024-59387_SourceDataForFigure3F/3F Replicate 1/western GAPDH.tif]

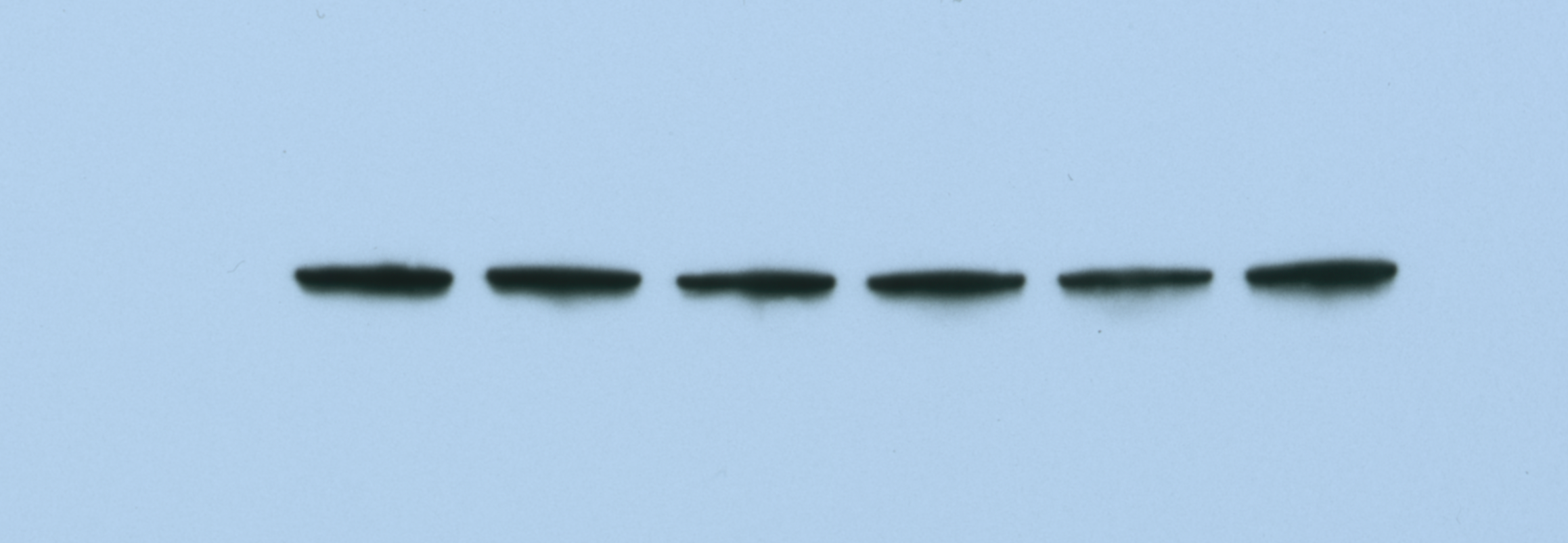

Supplement: Supplementary file 6 — Source data Fig. 3 [file 44319_2024_250_MOESM6_ESM.zip › EMBOR-2024-59387_SourceDataForFigure3/EMBOR-2024-59387_SourceDataForFigure3F/3F Replicate 1/western GR.tif]

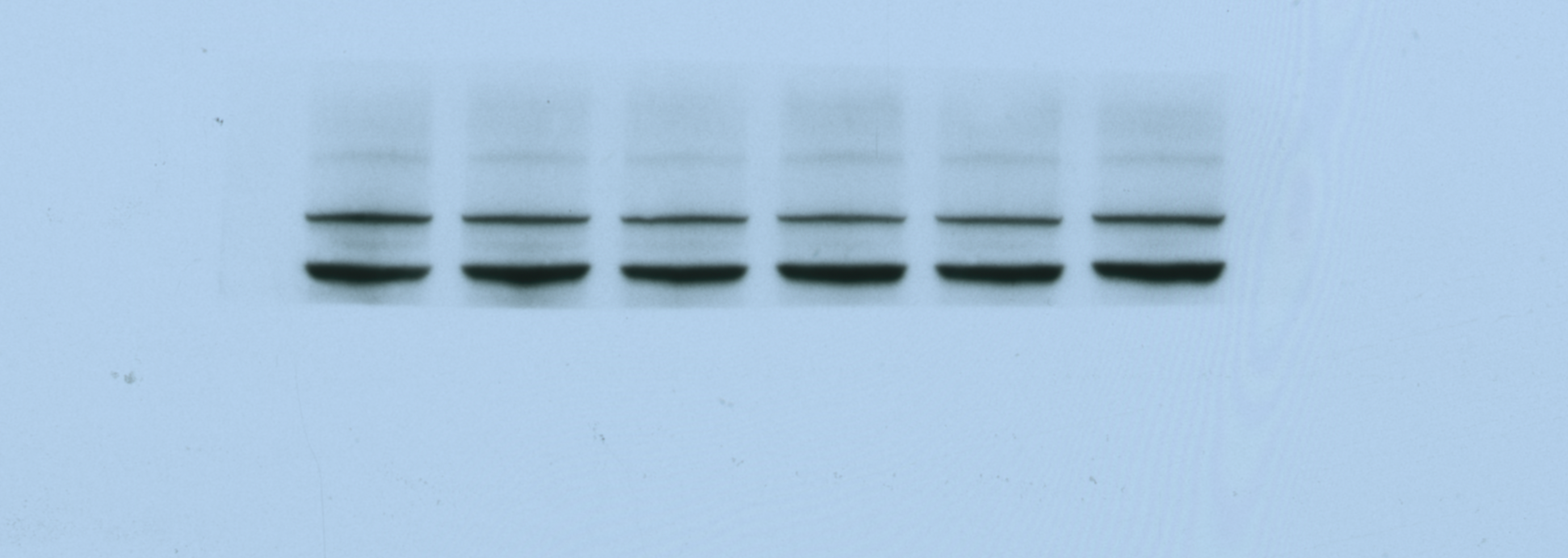

Supplement: Supplementary file 6 — Source data Fig. 3 [file 44319_2024_250_MOESM6_ESM.zip › EMBOR-2024-59387_SourceDataForFigure3/EMBOR-2024-59387_SourceDataForFigure3F/3F Replicate 1/western Hsp90.tif]

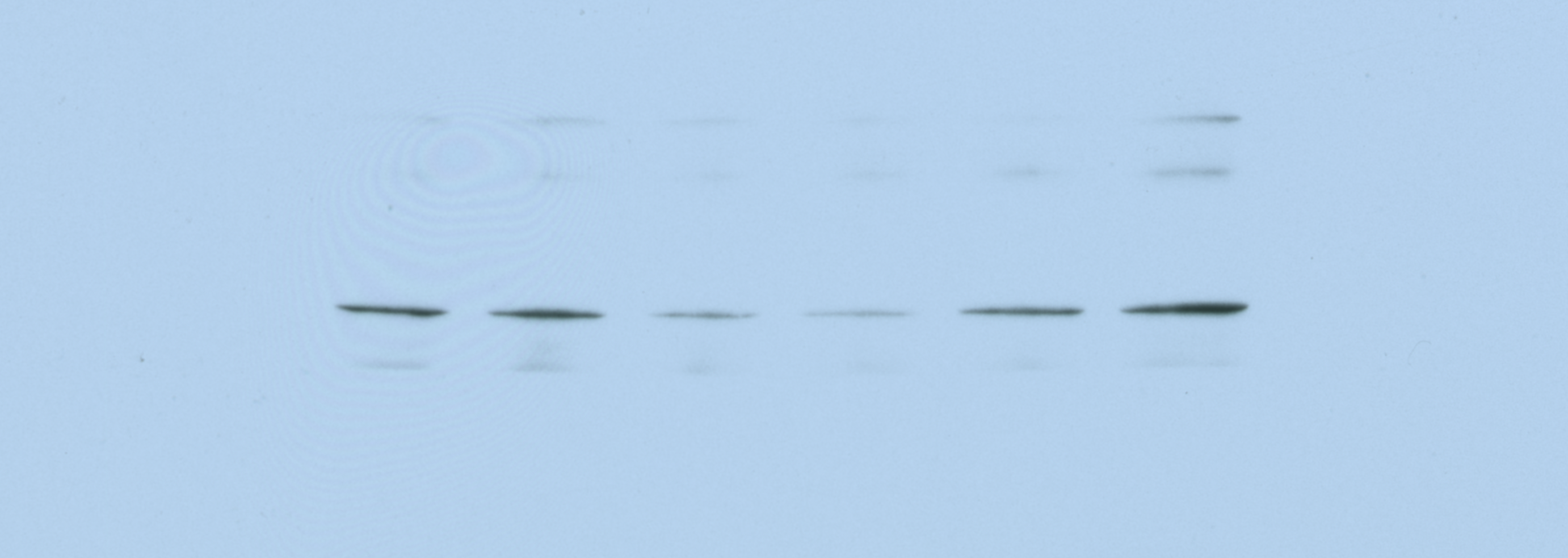

Supplement: Supplementary file 6 — Source data Fig. 3 [file 44319_2024_250_MOESM6_ESM.zip › EMBOR-2024-59387_SourceDataForFigure3/EMBOR-2024-59387_SourceDataForFigure3F/3F Replicate 1/western pGR-S211.tif]

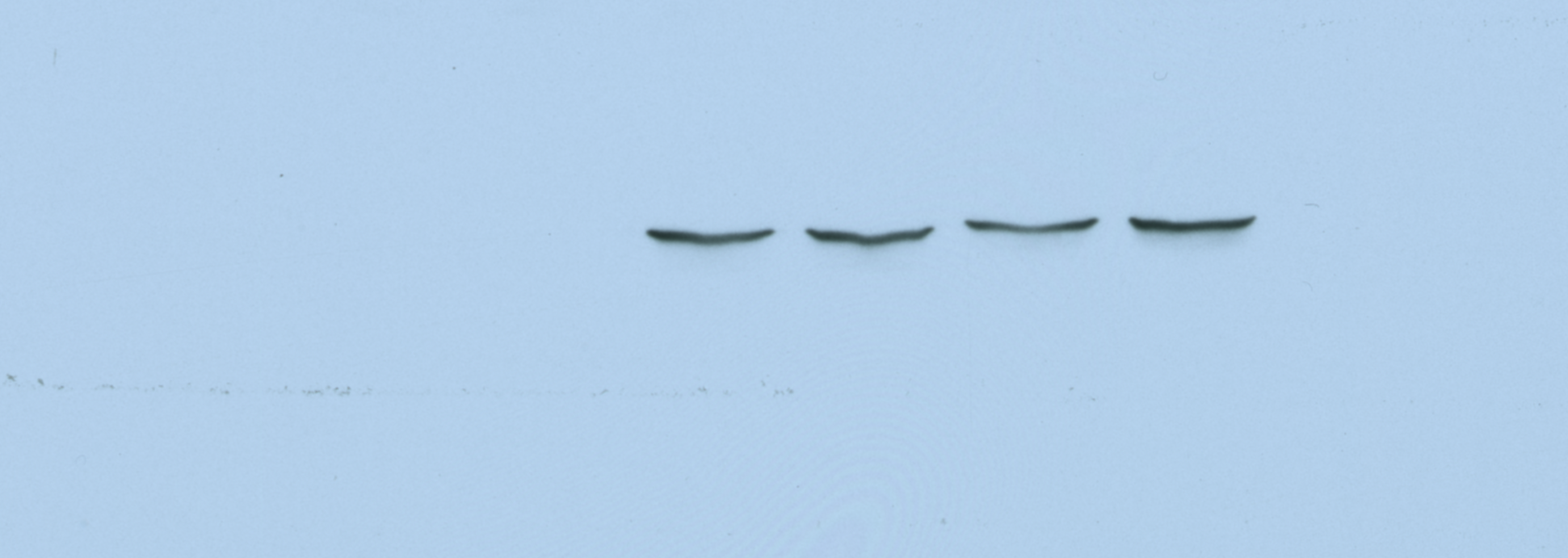

Supplement: Supplementary file 6 — Source data Fig. 3 [file 44319_2024_250_MOESM6_ESM.zip › EMBOR-2024-59387_SourceDataForFigure3/EMBOR-2024-59387_SourceDataForFigure3F/3F Replicate 1/western PP5-FLAG.tif]

# 3F-Replicate 1

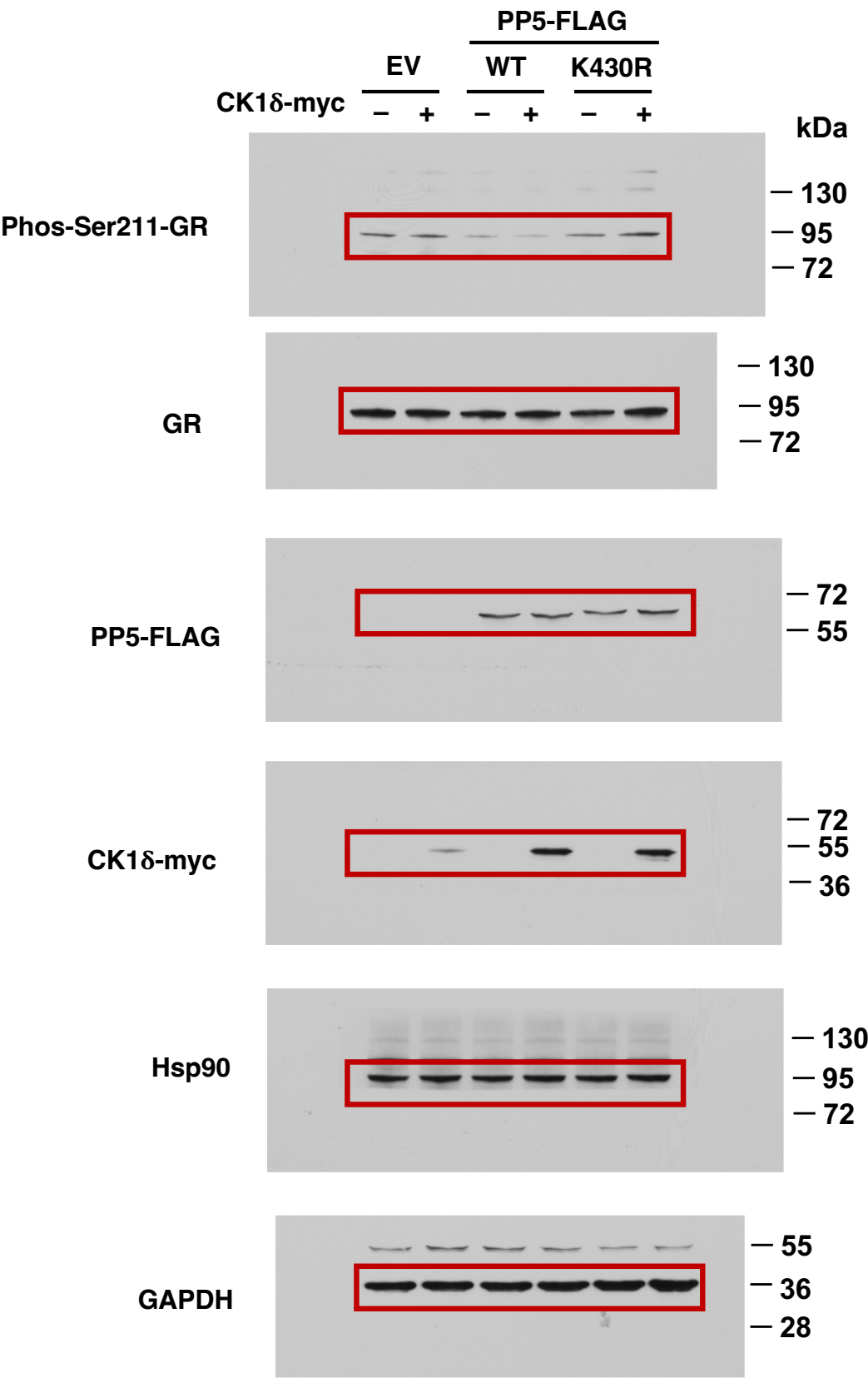

Supplement: Supplementary file 6 — Source data Fig. 3 [file 44319_2024_250_MOESM6_ESM.zip › EMBOR-2024-59387_SourceDataForFigure3/EMBOR-2024-59387_SourceDataForFigure3F/3F Replicate 1/western uncropped annotated.pdf]

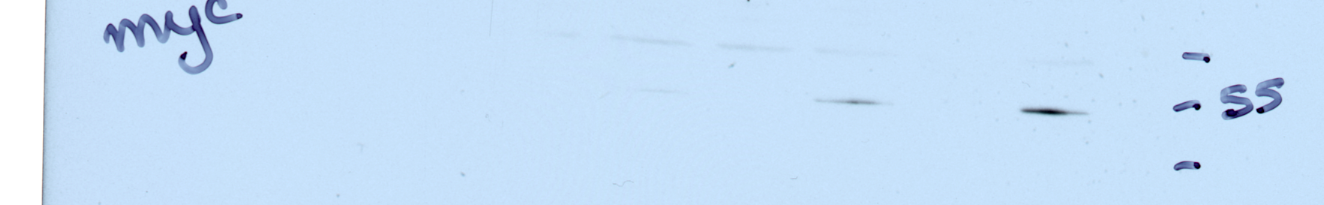

Supplement: Supplementary file 6 — Source data Fig. 3 [file 44319_2024_250_MOESM6_ESM.zip › EMBOR-2024-59387_SourceDataForFigure3/EMBOR-2024-59387_SourceDataForFigure3F/3F Replicate 2/western CK1-myc.tif]

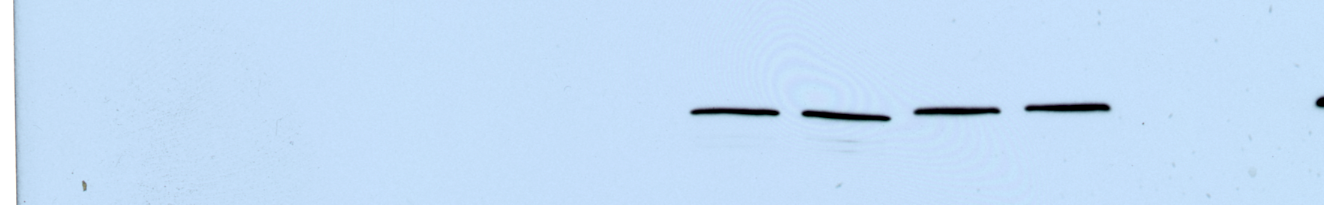

Supplement: Supplementary file 6 — Source data Fig. 3 [file 44319_2024_250_MOESM6_ESM.zip › EMBOR-2024-59387_SourceDataForFigure3/EMBOR-2024-59387_SourceDataForFigure3F/3F Replicate 2/western FLAG IP.tif]

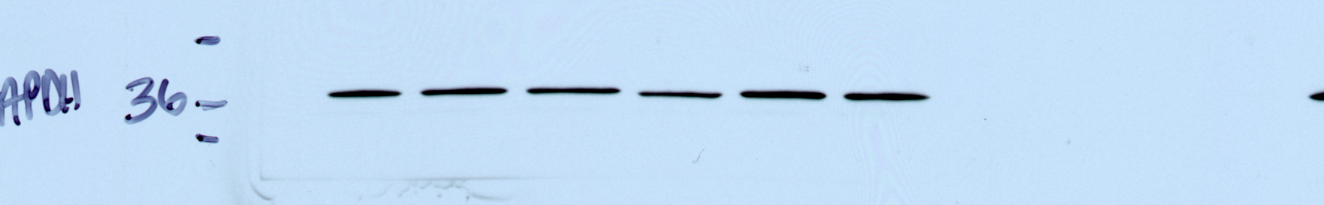

Supplement: Supplementary file 6 — Source data Fig. 3 [file 44319_2024_250_MOESM6_ESM.zip › EMBOR-2024-59387_SourceDataForFigure3/EMBOR-2024-59387_SourceDataForFigure3F/3F Replicate 2/western GAPDH.tif]

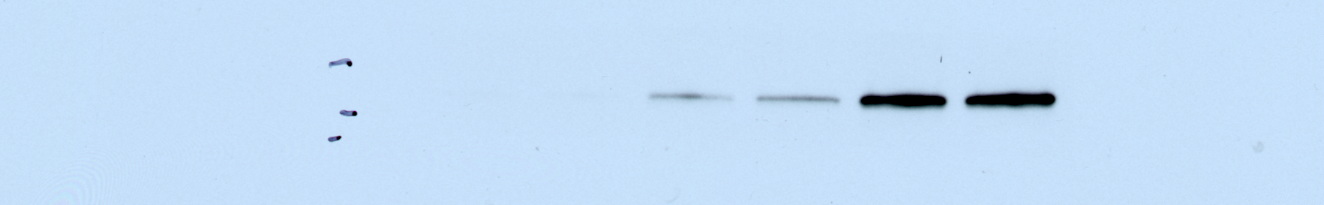

Supplement: Supplementary file 6 — Source data Fig. 3 [file 44319_2024_250_MOESM6_ESM.zip › EMBOR-2024-59387_SourceDataForFigure3/EMBOR-2024-59387_SourceDataForFigure3F/3F Replicate 2/western GR Co-IP.tif]

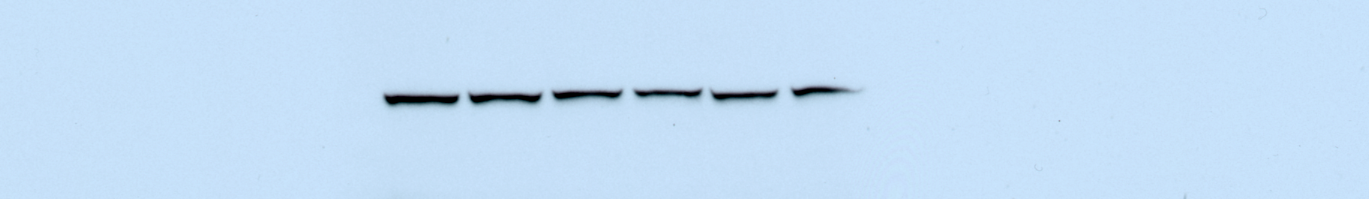

Supplement: Supplementary file 6 — Source data Fig. 3 [file 44319_2024_250_MOESM6_ESM.zip › EMBOR-2024-59387_SourceDataForFigure3/EMBOR-2024-59387_SourceDataForFigure3F/3F Replicate 2/western GR.tif]

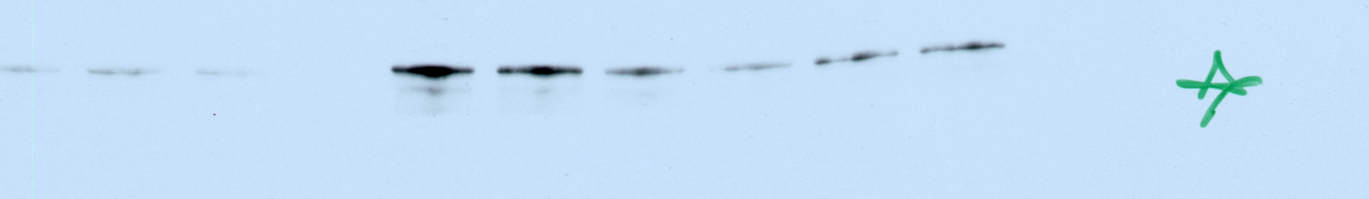

Supplement: Supplementary file 6 — Source data Fig. 3 [file 44319_2024_250_MOESM6_ESM.zip › EMBOR-2024-59387_SourceDataForFigure3/EMBOR-2024-59387_SourceDataForFigure3F/3F Replicate 2/western pS211-GR.tif]

# 3F-Replicate 2

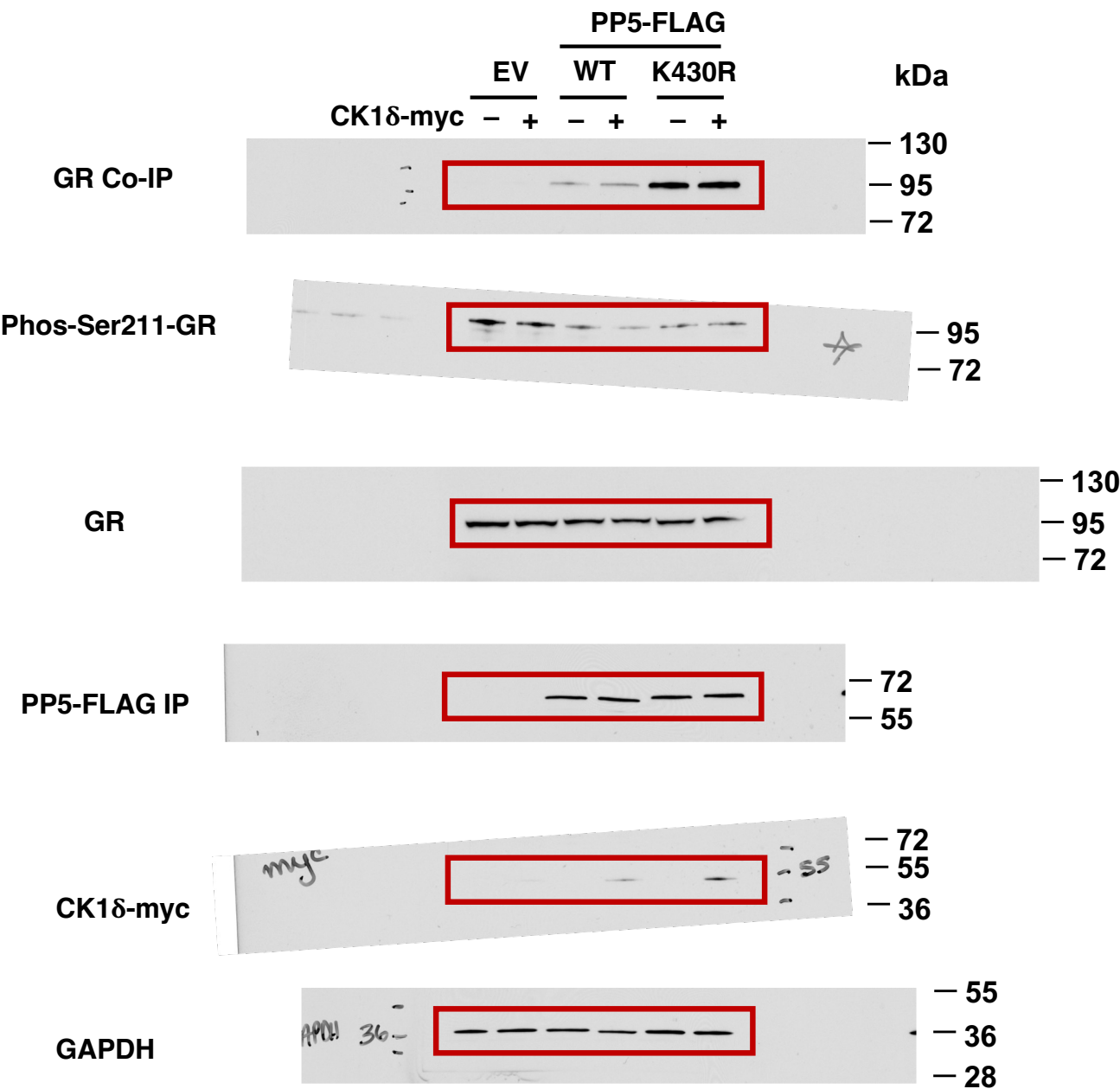

Supplement: Supplementary file 6 — Source data Fig. 3 [file 44319_2024_250_MOESM6_ESM.zip › EMBOR-2024-59387_SourceDataForFigure3/EMBOR-2024-59387_SourceDataForFigure3F/3F Replicate 2/western uncropped annotated.pdf]

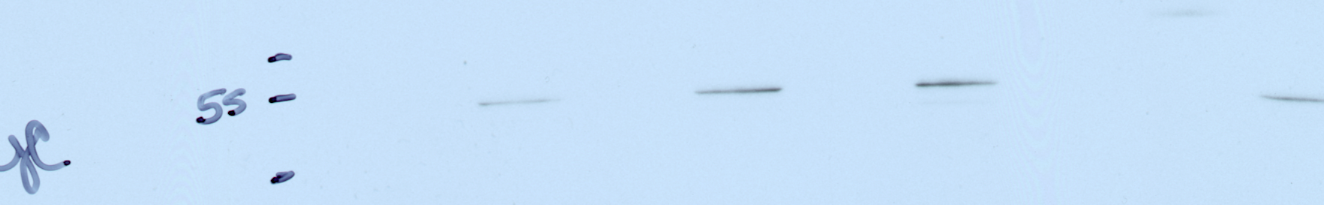

Supplement: Supplementary file 6 — Source data Fig. 3 [file 44319_2024_250_MOESM6_ESM.zip › EMBOR-2024-59387_SourceDataForFigure3/EMBOR-2024-59387_SourceDataForFigure3F/3F Replicate 3/western CK1-myc.tif]

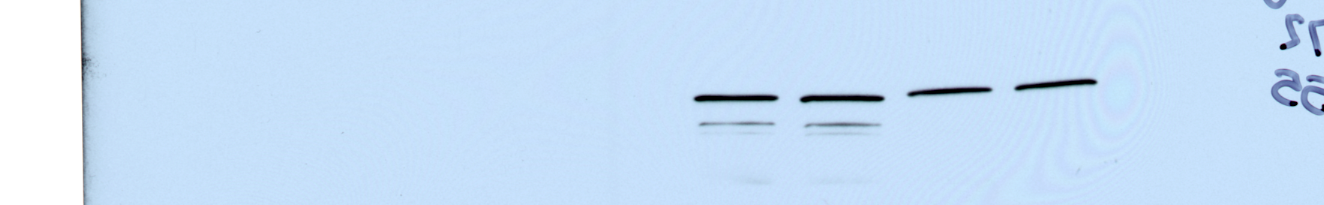

Supplement: Supplementary file 6 — Source data Fig. 3 [file 44319_2024_250_MOESM6_ESM.zip › EMBOR-2024-59387_SourceDataForFigure3/EMBOR-2024-59387_SourceDataForFigure3F/3F Replicate 3/western FLAG IP.tif]

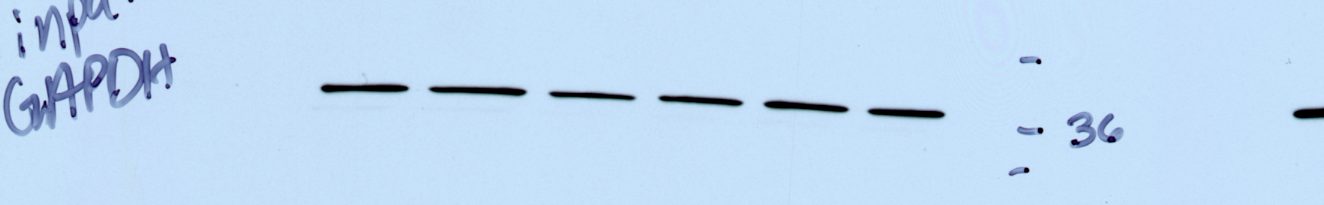

Supplement: Supplementary file 6 — Source data Fig. 3 [file 44319_2024_250_MOESM6_ESM.zip › EMBOR-2024-59387_SourceDataForFigure3/EMBOR-2024-59387_SourceDataForFigure3F/3F Replicate 3/western GAPDH.tif]

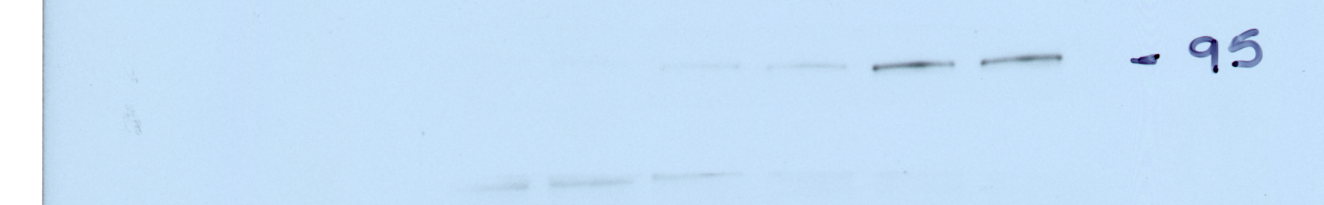

Supplement: Supplementary file 6 — Source data Fig. 3 [file 44319_2024_250_MOESM6_ESM.zip › EMBOR-2024-59387_SourceDataForFigure3/EMBOR-2024-59387_SourceDataForFigure3F/3F Replicate 3/western GR Co-IP.tif]

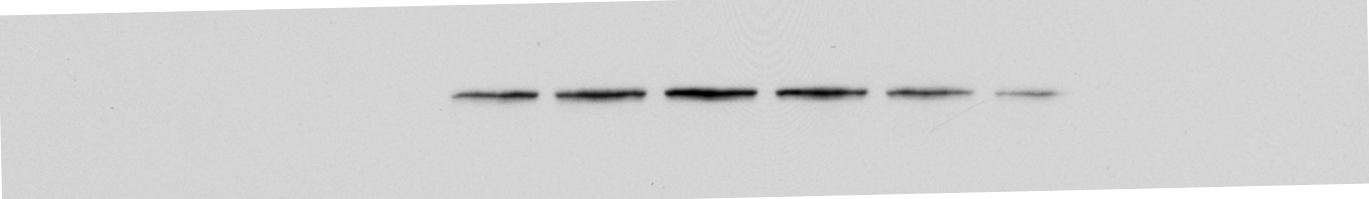

Supplement: Supplementary file 6 — Source data Fig. 3 [file 44319_2024_250_MOESM6_ESM.zip › EMBOR-2024-59387_SourceDataForFigure3/EMBOR-2024-59387_SourceDataForFigure3F/3F Replicate 3/western GR.tif]

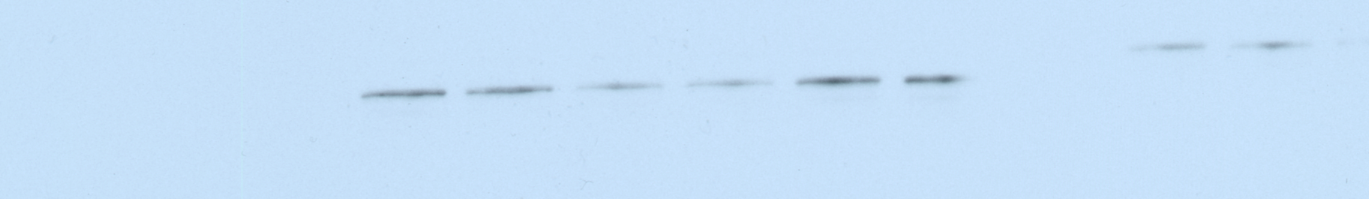

Supplement: Supplementary file 6 — Source data Fig. 3 [file 44319_2024_250_MOESM6_ESM.zip › EMBOR-2024-59387_SourceDataForFigure3/EMBOR-2024-59387_SourceDataForFigure3F/3F Replicate 3/western pS211-GR.tif]

# 3F-Replicate 3

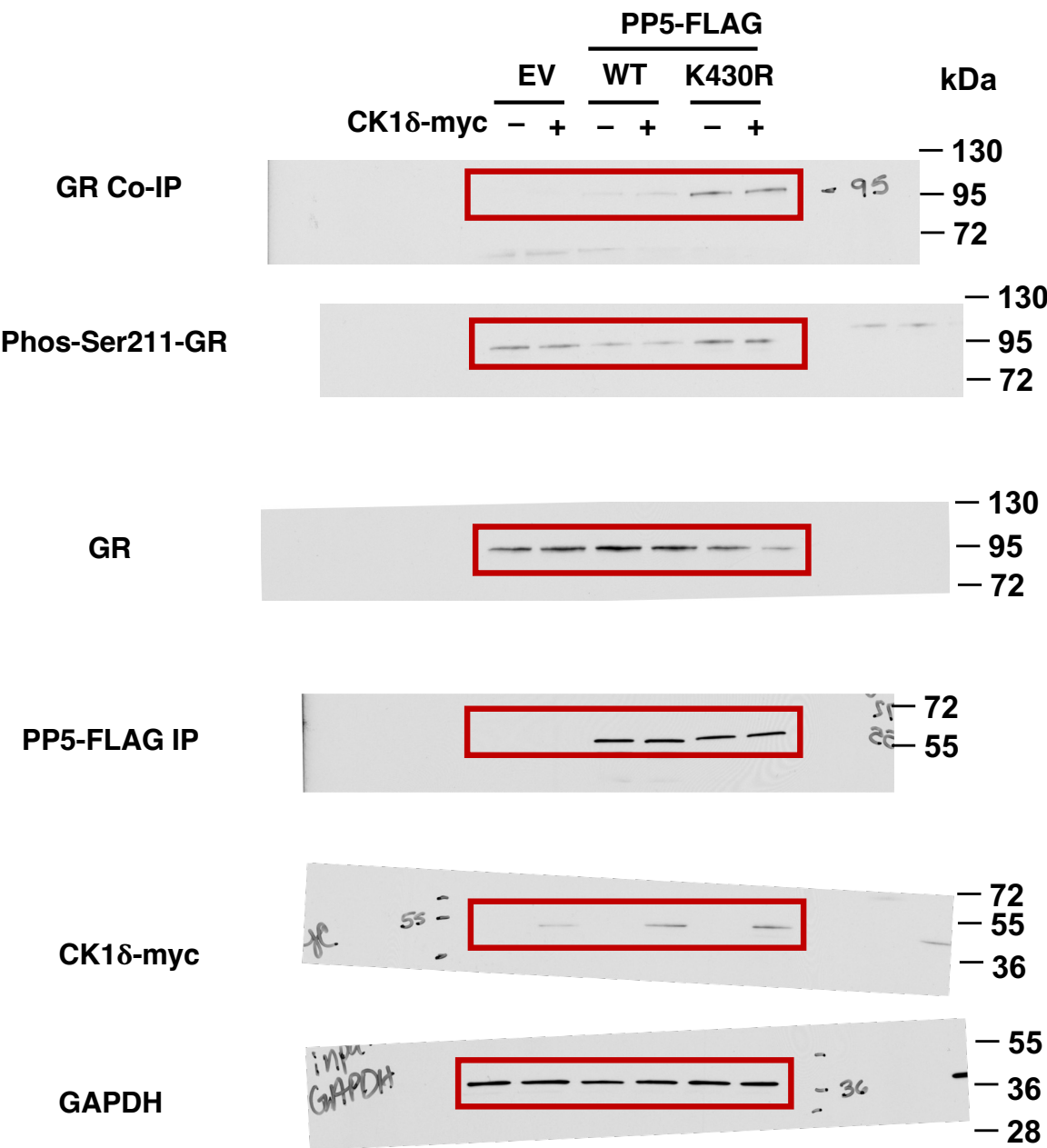

Supplement: Supplementary file 6 — Source data Fig. 3 [file 44319_2024_250_MOESM6_ESM.zip › EMBOR-2024-59387_SourceDataForFigure3/EMBOR-2024-59387_SourceDataForFigure3F/3F Replicate 3/western uncropped annotated.pdf]

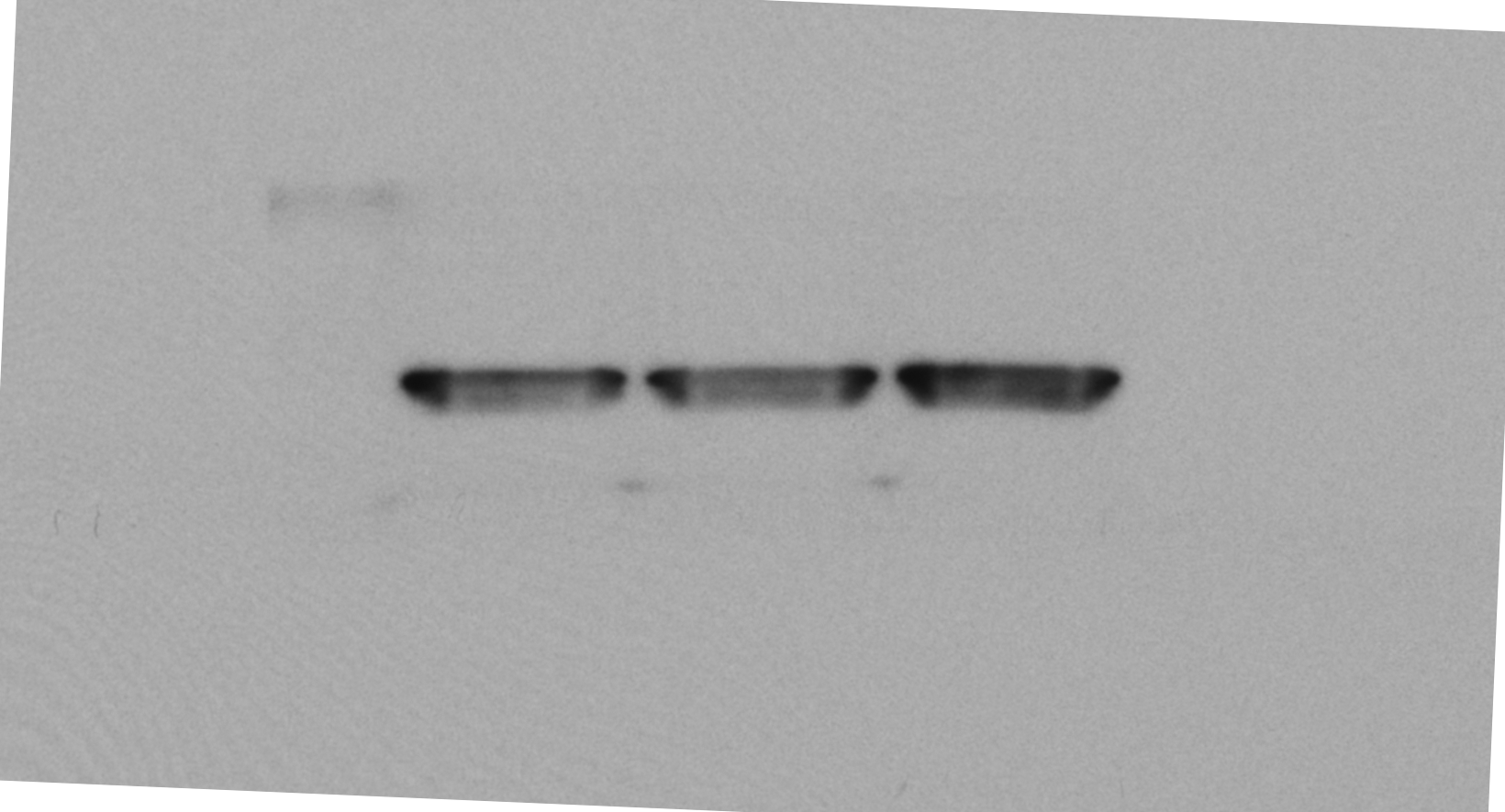

Supplement: Supplementary file 7 — Source data Fig. 4 [file 44319_2024_250_MOESM7_ESM.zip › EMBOR-2024-59387_SourceDataForFigure4/EMBOR-2024-59387_SourceDataForFigure4A/western GAPDH.tif]

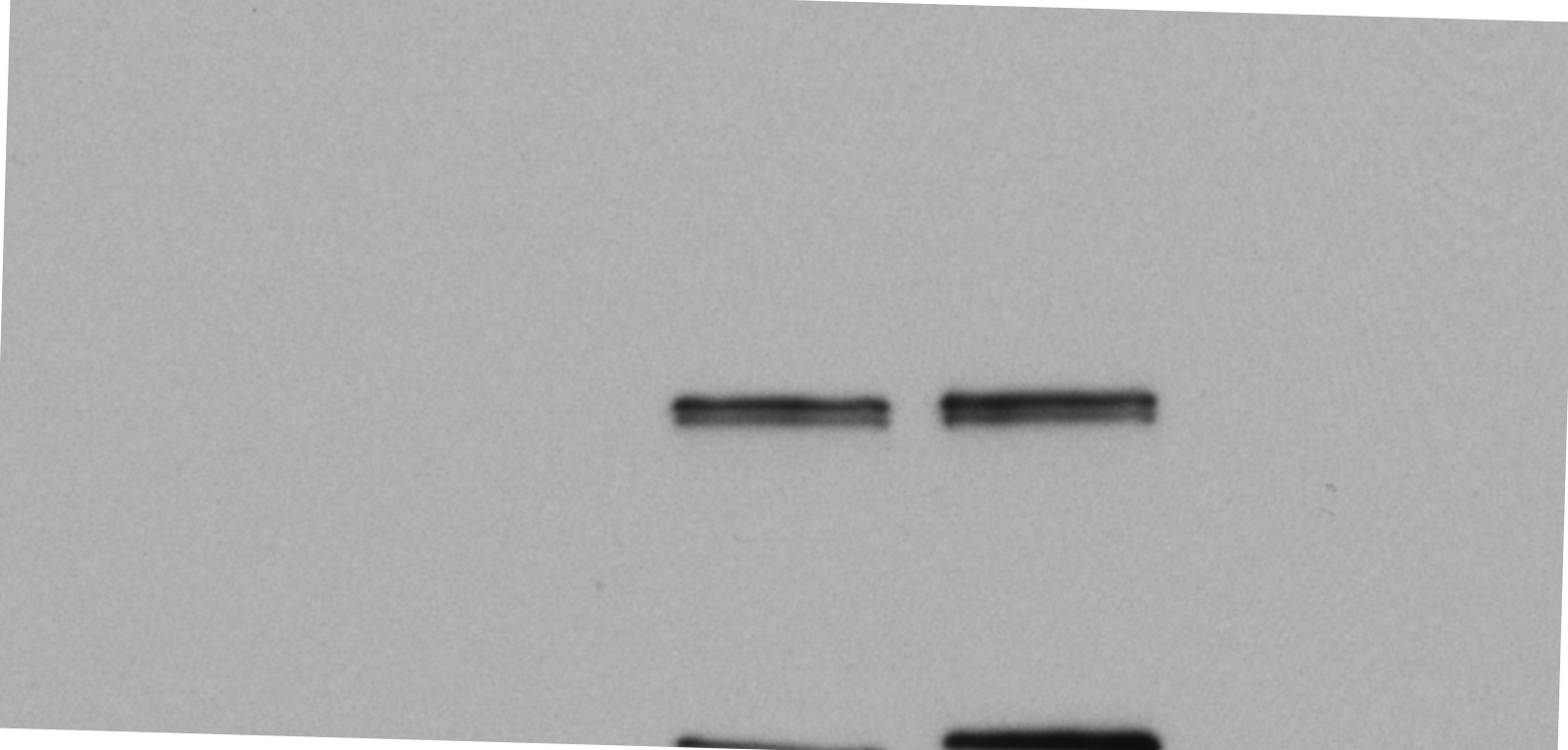

Supplement: Supplementary file 7 — Source data Fig. 4 [file 44319_2024_250_MOESM7_ESM.zip › EMBOR-2024-59387_SourceDataForFigure4/EMBOR-2024-59387_SourceDataForFigure4A/western GR IP.tif]

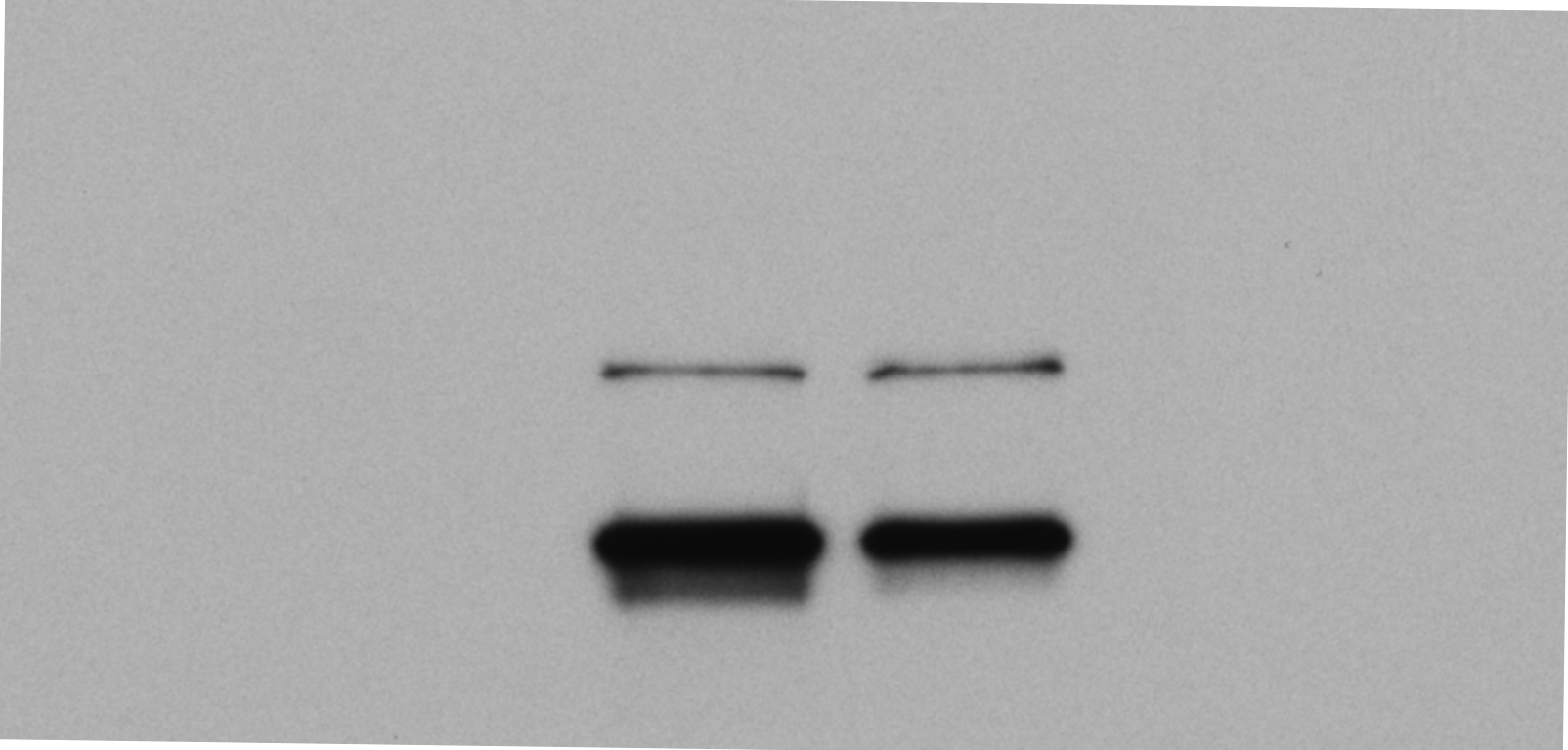

Supplement: Supplementary file 7 — Source data Fig. 4 [file 44319_2024_250_MOESM7_ESM.zip › EMBOR-2024-59387_SourceDataForFigure4/EMBOR-2024-59387_SourceDataForFigure4A/western Hsp90 coIP.tif]

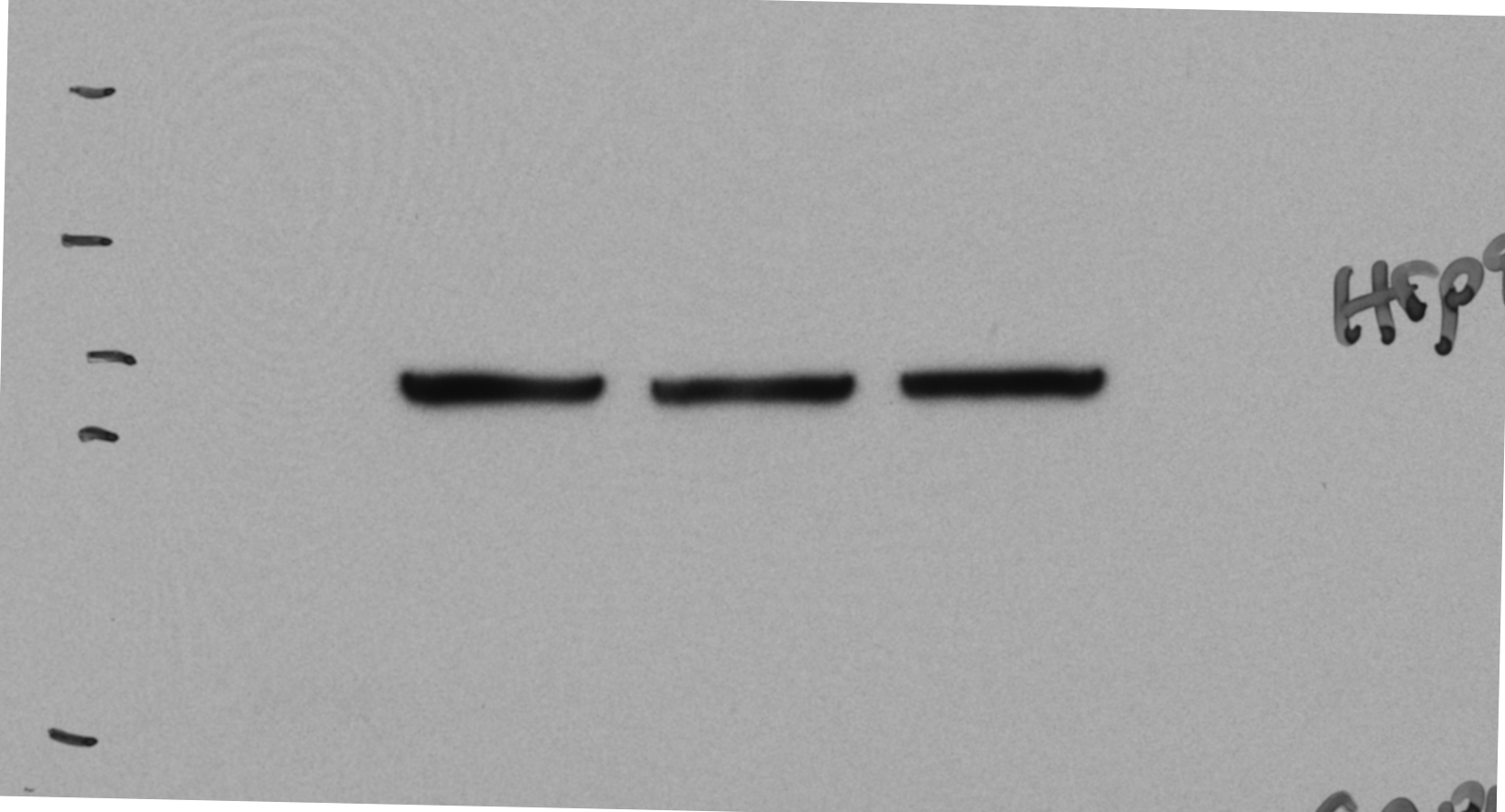

Supplement: Supplementary file 7 — Source data Fig. 4 [file 44319_2024_250_MOESM7_ESM.zip › EMBOR-2024-59387_SourceDataForFigure4/EMBOR-2024-59387_SourceDataForFigure4A/western Hsp90.tif]

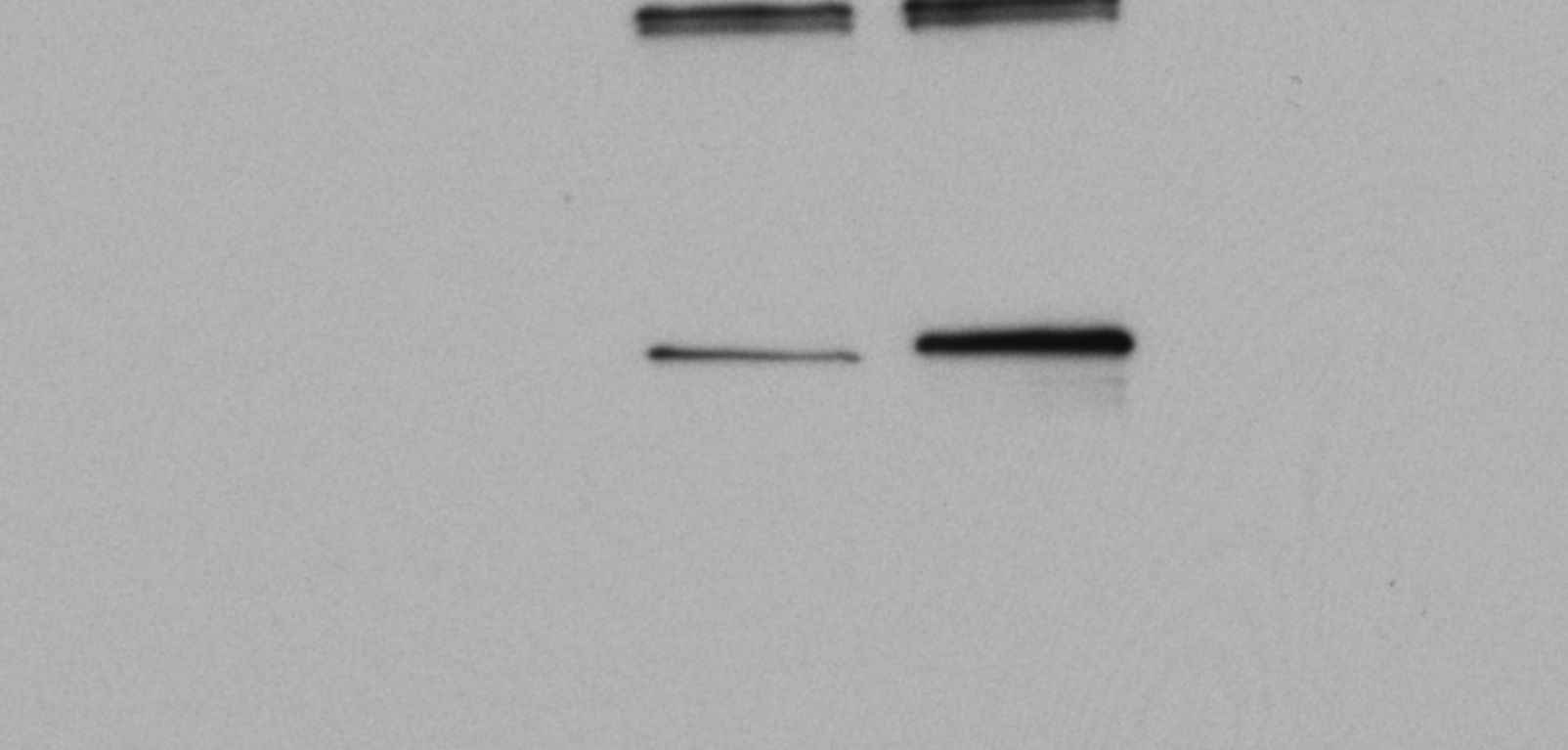

Supplement: Supplementary file 7 — Source data Fig. 4 [file 44319_2024_250_MOESM7_ESM.zip › EMBOR-2024-59387_SourceDataForFigure4/EMBOR-2024-59387_SourceDataForFigure4A/western PP5-FLAG IP.tif]
