# Supplementary figures and images for: SUMOylation of protein phosphatase 5 regulates phosphatase activity and substrate release (part 2 of 2)
Source: EMBO Rep. 2024 Sep 20;25(11):4. doi: 10.1038/s44319-024-00250-2 (PMC11549447; doi:10.1038/s44319-024-00250-2)

4A

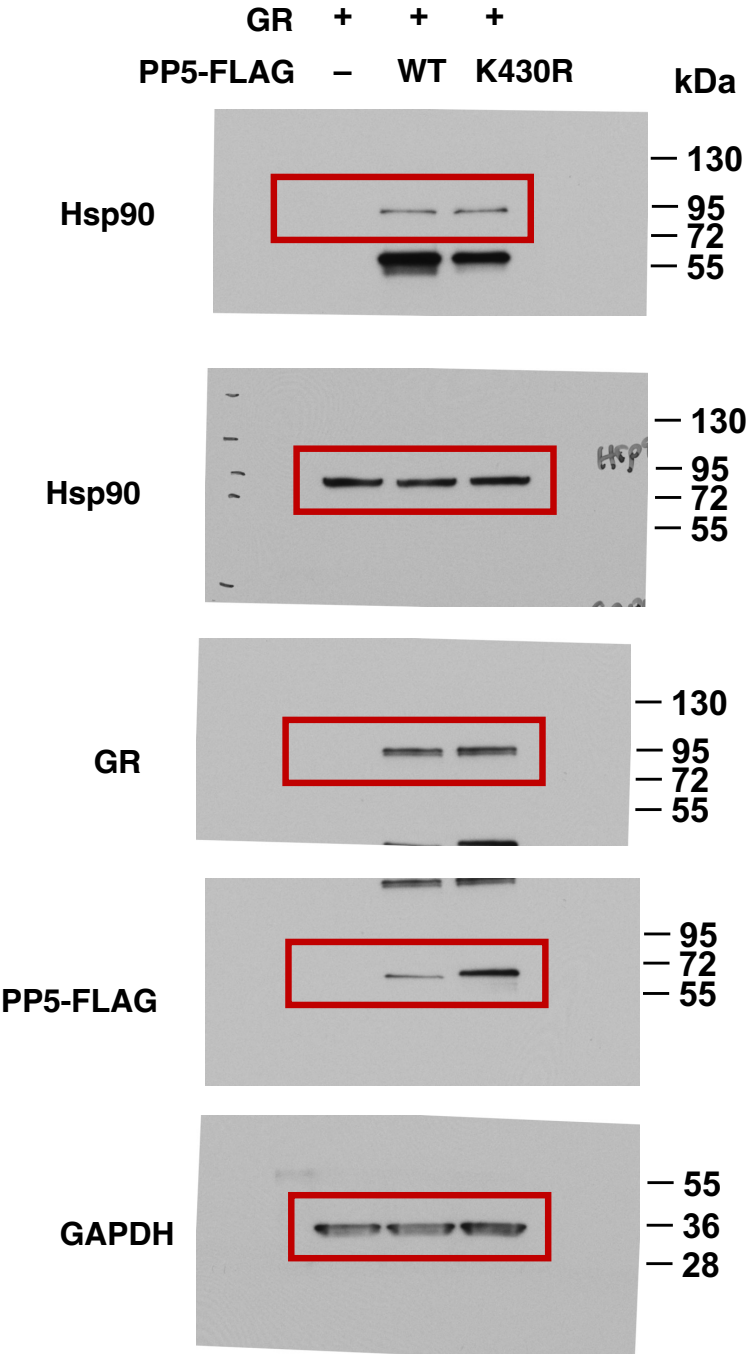

Supplement: Supplementary file 7 — Source data Fig. 4 [file 44319_2024_250_MOESM7_ESM.zip › EMBOR-2024-59387_SourceDataForFigure4/EMBOR-2024-59387_SourceDataForFigure4A/western uncropped annotated.pdf]

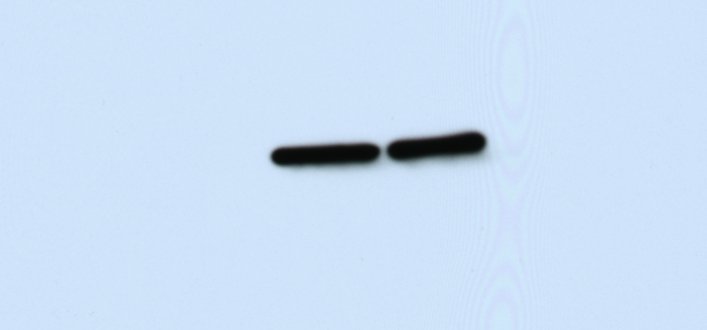

Supplement: Supplementary file 7 — Source data Fig. 4 [file 44319_2024_250_MOESM7_ESM.zip › EMBOR-2024-59387_SourceDataForFigure4/EMBOR-2024-59387_SourceDataForFigure4B/westen GAPDH.jpg]

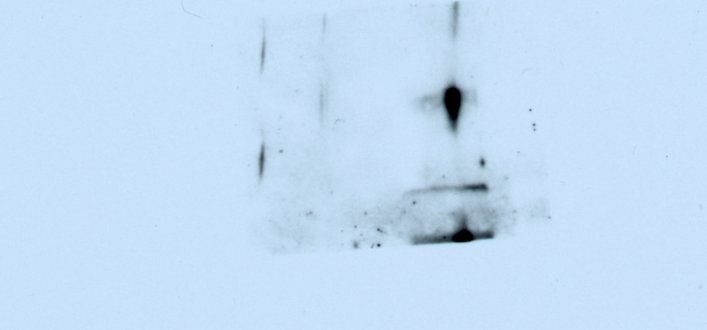

Supplement: Supplementary file 7 — Source data Fig. 4 [file 44319_2024_250_MOESM7_ESM.zip › EMBOR-2024-59387_SourceDataForFigure4/EMBOR-2024-59387_SourceDataForFigure4B/western Hsp90 coIP.jpg]

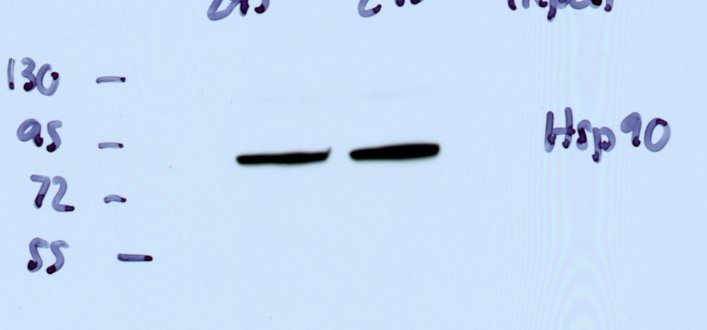

Supplement: Supplementary file 7 — Source data Fig. 4 [file 44319_2024_250_MOESM7_ESM.zip › EMBOR-2024-59387_SourceDataForFigure4/EMBOR-2024-59387_SourceDataForFigure4B/western Hsp90.jpg]

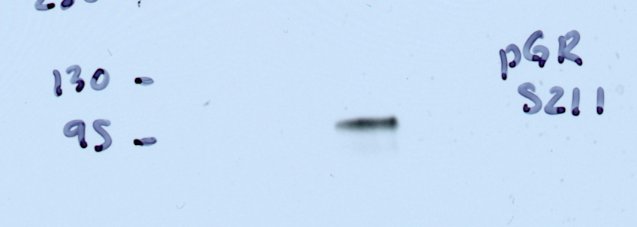

Supplement: Supplementary file 7 — Source data Fig. 4 [file 44319_2024_250_MOESM7_ESM.zip › EMBOR-2024-59387_SourceDataForFigure4/EMBOR-2024-59387_SourceDataForFigure4B/western pGR S211 IP.jpg]

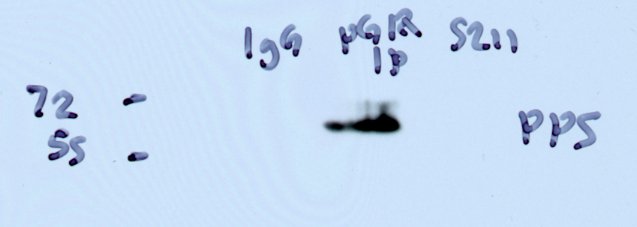

Supplement: Supplementary file 7 — Source data Fig. 4 [file 44319_2024_250_MOESM7_ESM.zip › EMBOR-2024-59387_SourceDataForFigure4/EMBOR-2024-59387_SourceDataForFigure4B/western PP5 coIP.jpg]

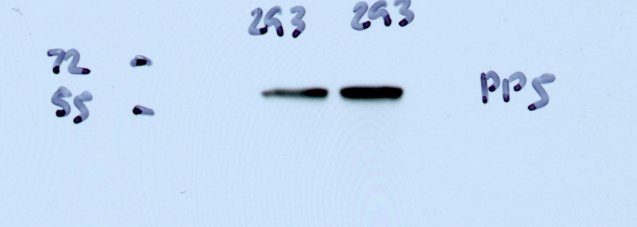

Supplement: Supplementary file 7 — Source data Fig. 4 [file 44319_2024_250_MOESM7_ESM.zip › EMBOR-2024-59387_SourceDataForFigure4/EMBOR-2024-59387_SourceDataForFigure4B/western PP5.jpg]

4B

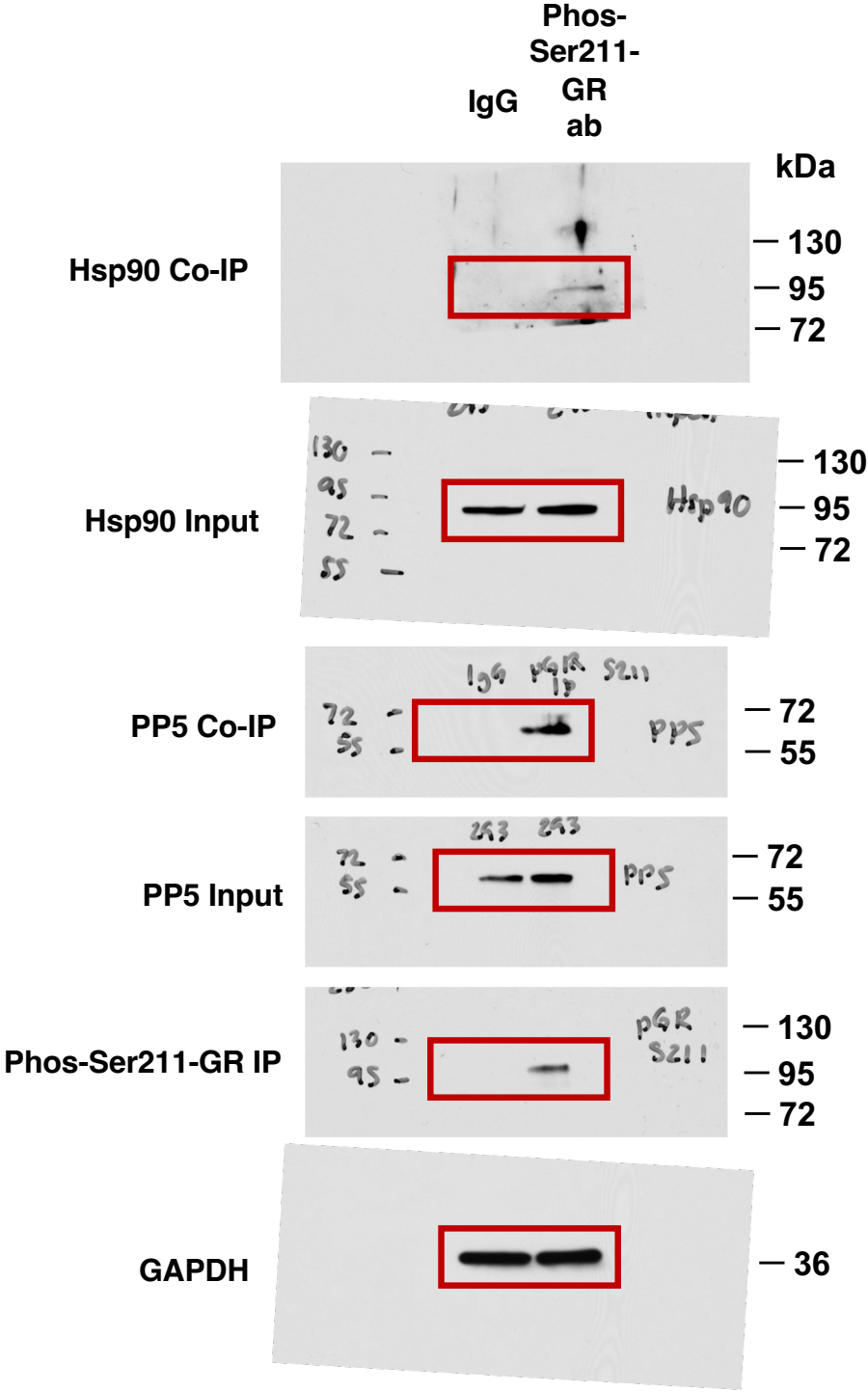

Supplement: Supplementary file 7 — Source data Fig. 4 [file 44319_2024_250_MOESM7_ESM.zip › EMBOR-2024-59387_SourceDataForFigure4/EMBOR-2024-59387_SourceDataForFigure4B/western uncropped annotated.pdf]

4E

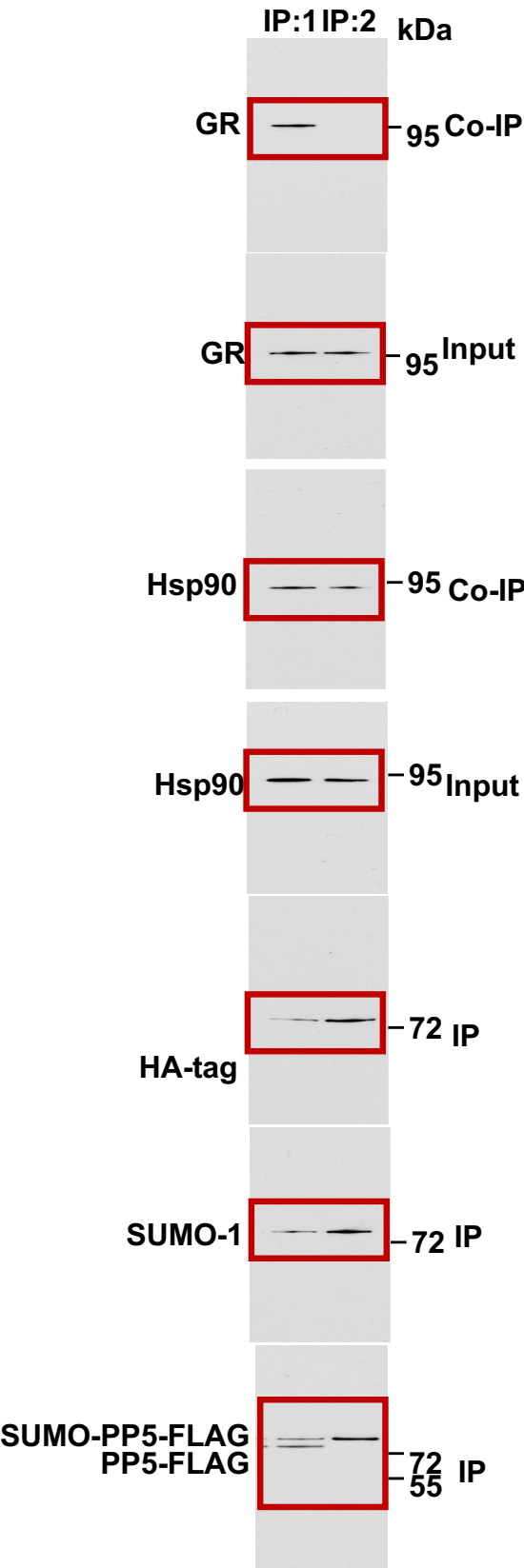

Supplement: Supplementary file 7 — Source data Fig. 4 [file 44319_2024_250_MOESM7_ESM.zip › EMBOR-2024-59387_SourceDataForFigure4/EMBOR-2024-59387_SourceDataForFigure4E/western uncropped annotated.pdf]

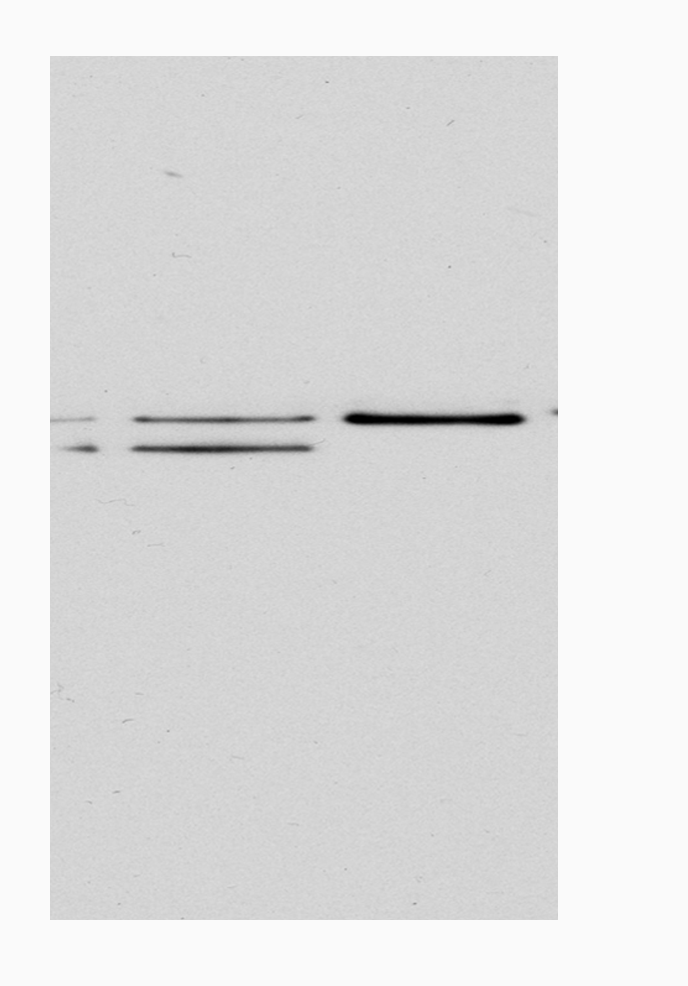

Supplement: Supplementary file 7 — Source data Fig. 4 [file 44319_2024_250_MOESM7_ESM.zip › EMBOR-2024-59387_SourceDataForFigure4/EMBOR-2024-59387_SourceDataForFigure4E/Western-FLAG -IP.tif]

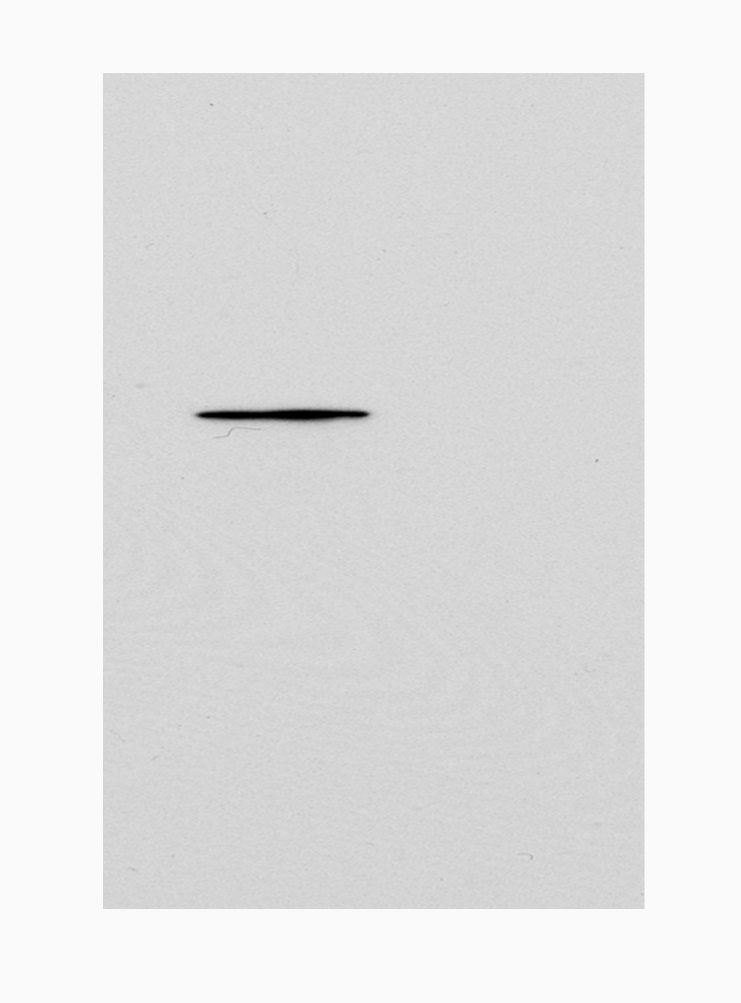

Supplement: Supplementary file 7 — Source data Fig. 4 [file 44319_2024_250_MOESM7_ESM.zip › EMBOR-2024-59387_SourceDataForFigure4/EMBOR-2024-59387_SourceDataForFigure4E/Western-GR-coIP.tif]

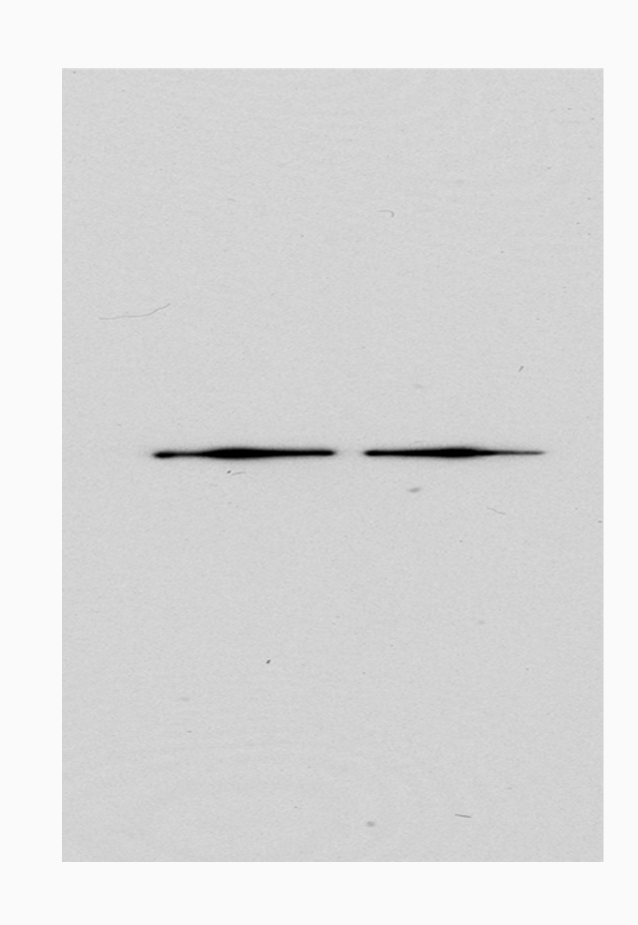

Supplement: Supplementary file 7 — Source data Fig. 4 [file 44319_2024_250_MOESM7_ESM.zip › EMBOR-2024-59387_SourceDataForFigure4/EMBOR-2024-59387_SourceDataForFigure4E/Western-GR-Input.tif]

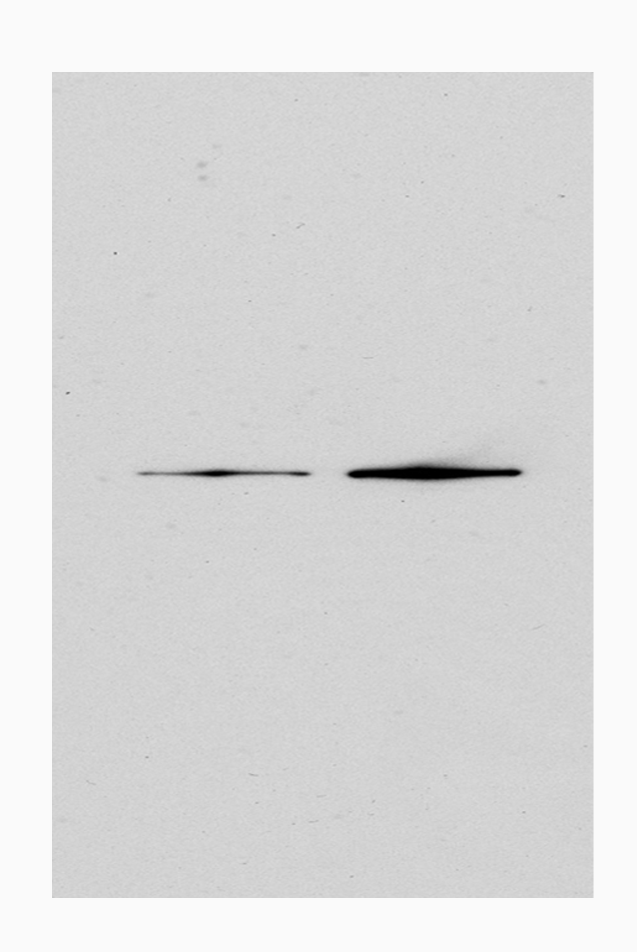

Supplement: Supplementary file 7 — Source data Fig. 4 [file 44319_2024_250_MOESM7_ESM.zip › EMBOR-2024-59387_SourceDataForFigure4/EMBOR-2024-59387_SourceDataForFigure4E/Western-HAtag-IP.tif]

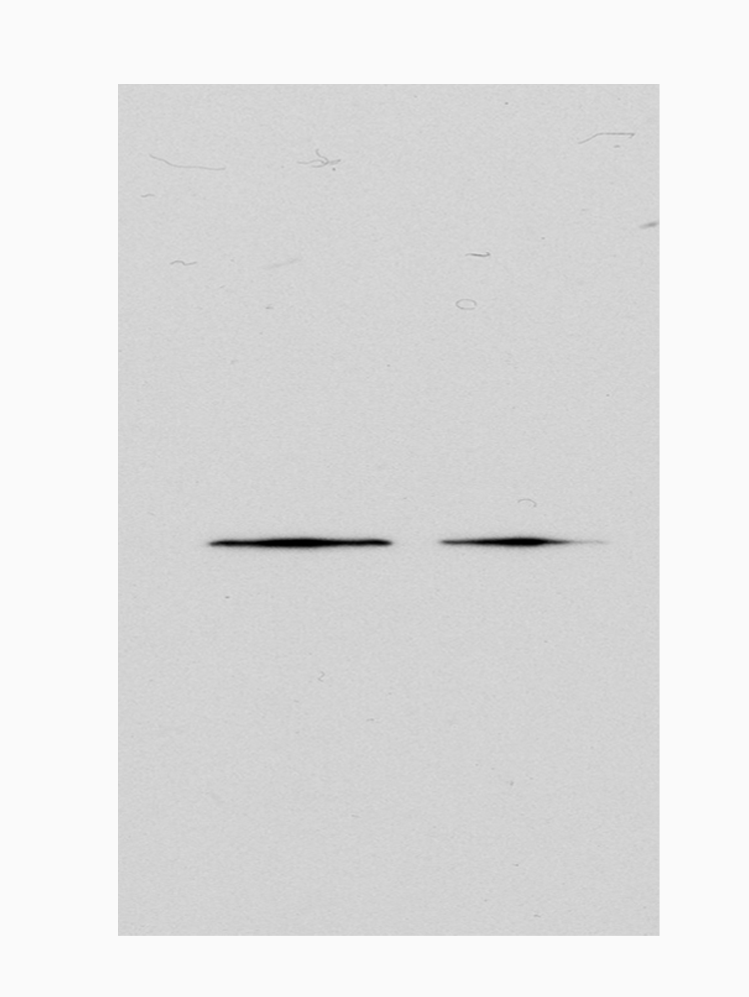

Supplement: Supplementary file 7 — Source data Fig. 4 [file 44319_2024_250_MOESM7_ESM.zip › EMBOR-2024-59387_SourceDataForFigure4/EMBOR-2024-59387_SourceDataForFigure4E/Western-Hsp90CoIP.tif]

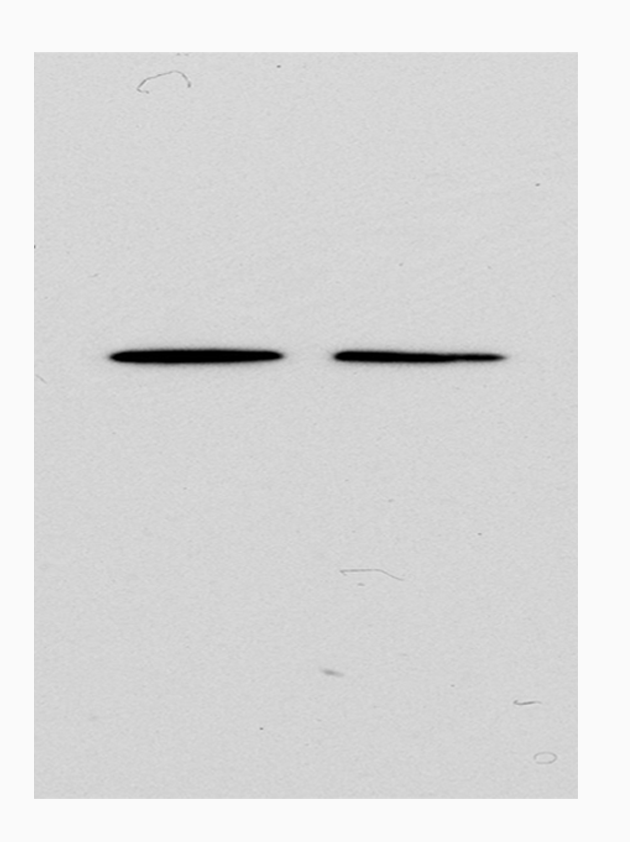

Supplement: Supplementary file 7 — Source data Fig. 4 [file 44319_2024_250_MOESM7_ESM.zip › EMBOR-2024-59387_SourceDataForFigure4/EMBOR-2024-59387_SourceDataForFigure4E/Western-Hsp90Input.tif]

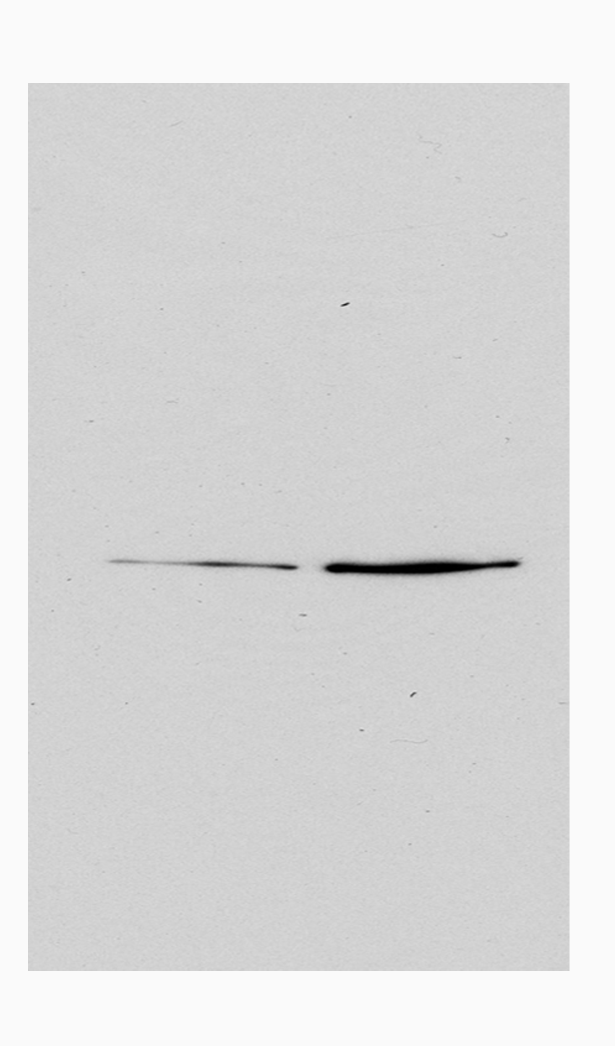

Supplement: Supplementary file 7 — Source data Fig. 4 [file 44319_2024_250_MOESM7_ESM.zip › EMBOR-2024-59387_SourceDataForFigure4/EMBOR-2024-59387_SourceDataForFigure4E/WesternSUMO1-IP.tif]

# 4C-Replicate 1

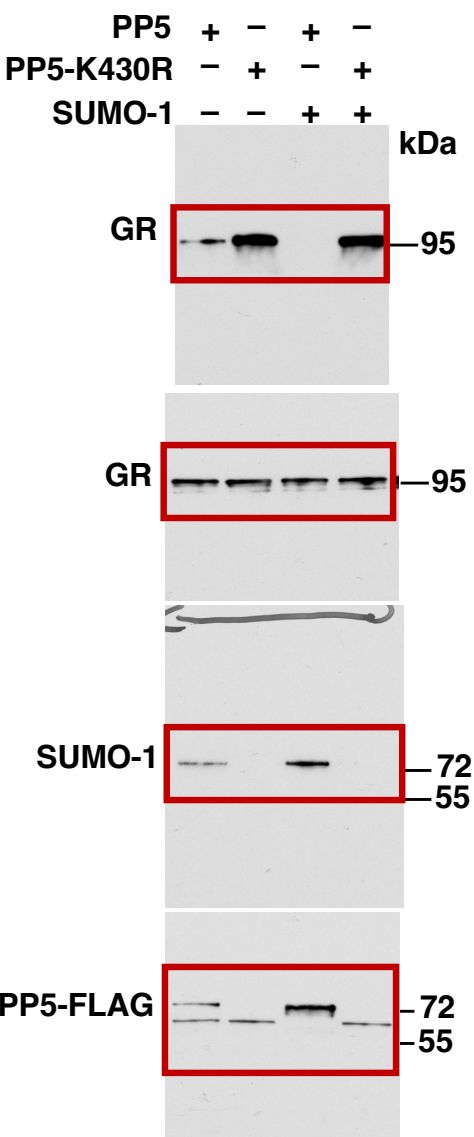

Supplement: Supplementary file 7 — Source data Fig. 4 [file 44319_2024_250_MOESM7_ESM.zip › EMBOR-2024-59387_SourceDataForFigure4/EMBOR-2024-59387_SourceDataForFigure4C/Replica-1/western uncropped annotated.pdf]

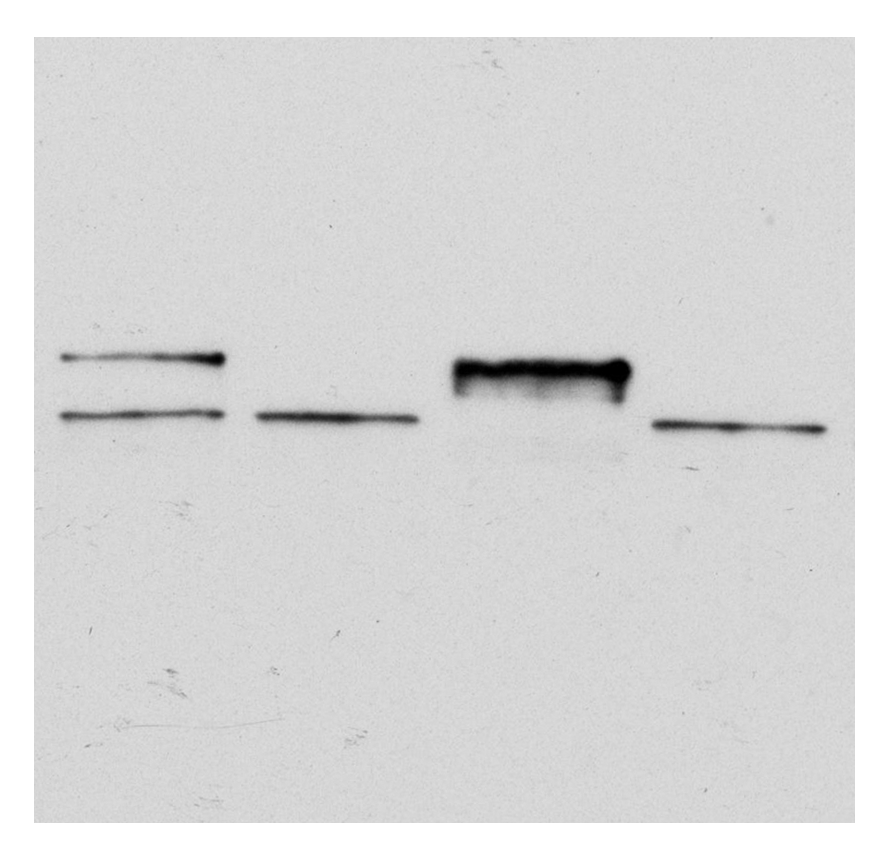

Supplement: Supplementary file 7 — Source data Fig. 4 [file 44319_2024_250_MOESM7_ESM.zip › EMBOR-2024-59387_SourceDataForFigure4/EMBOR-2024-59387_SourceDataForFigure4C/Replica-1/Western-FLAGPP5-IP-REP1.tif]

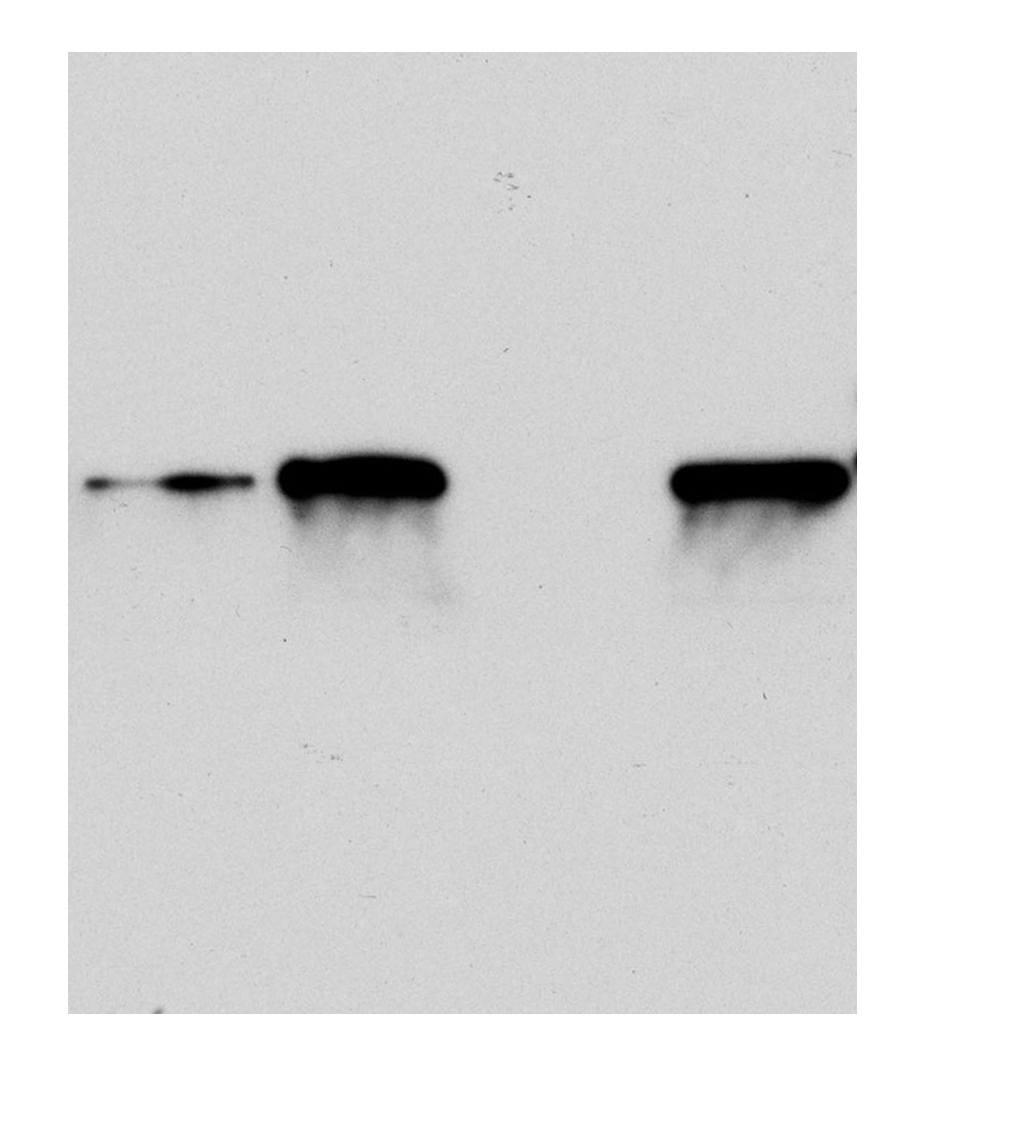

Supplement: Supplementary file 7 — Source data Fig. 4 [file 44319_2024_250_MOESM7_ESM.zip › EMBOR-2024-59387_SourceDataForFigure4/EMBOR-2024-59387_SourceDataForFigure4C/Replica-1/Western-GR-Co-IP-REP1.tif]

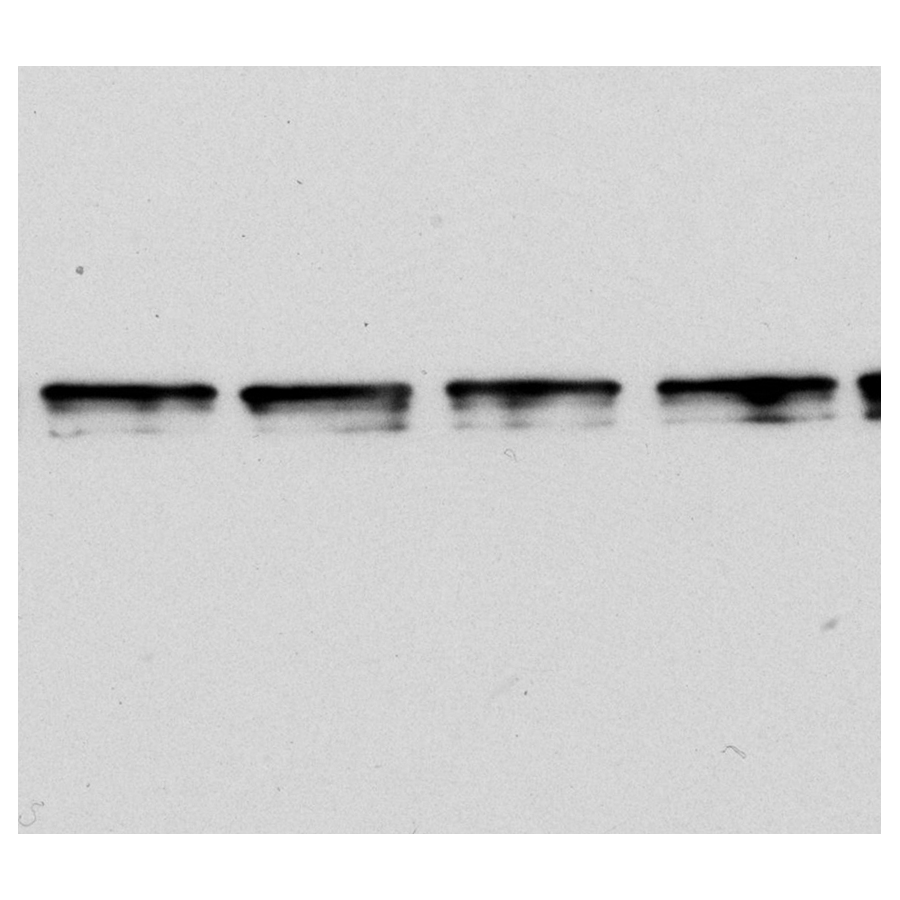

Supplement: Supplementary file 7 — Source data Fig. 4 [file 44319_2024_250_MOESM7_ESM.zip › EMBOR-2024-59387_SourceDataForFigure4/EMBOR-2024-59387_SourceDataForFigure4C/Replica-1/Western-GR-input-REP1.tif]

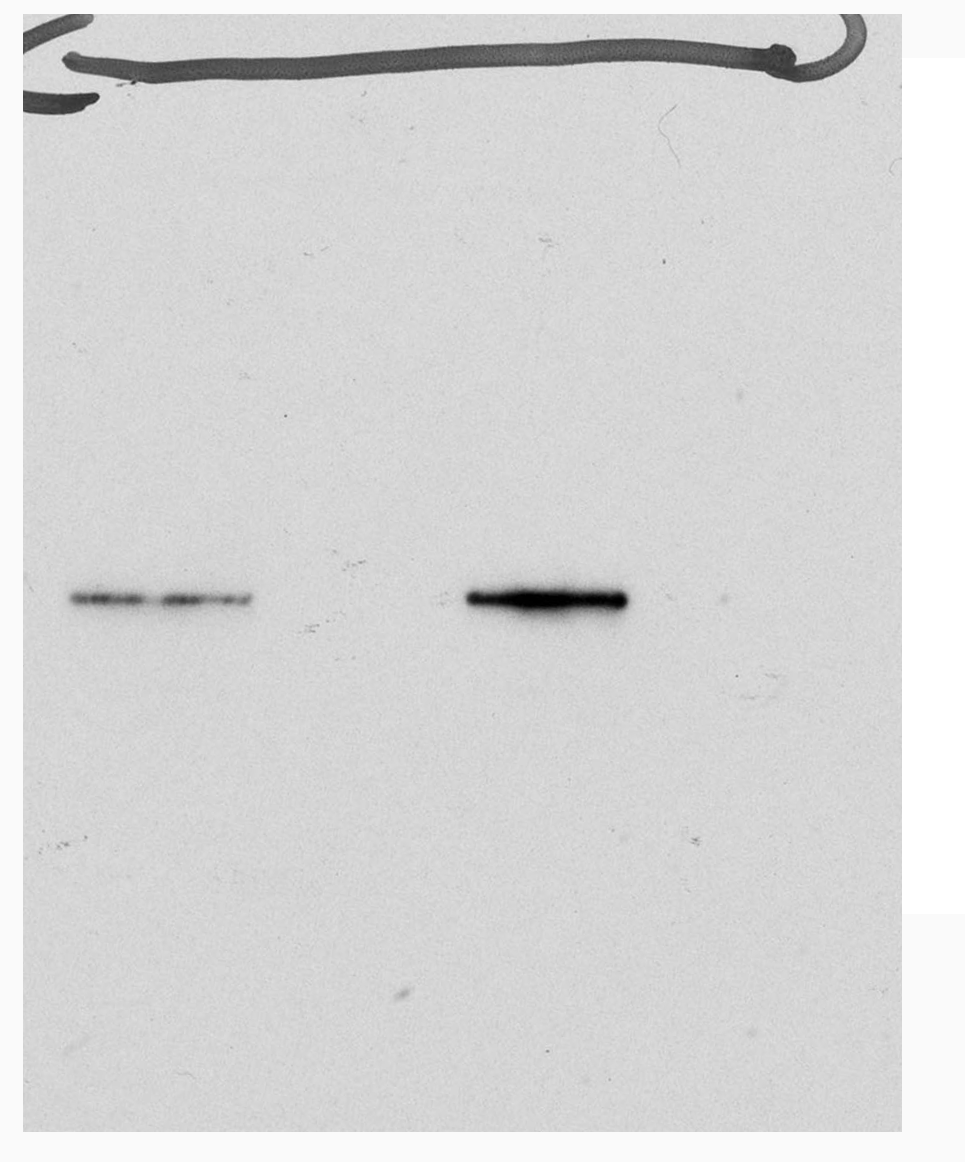

Supplement: Supplementary file 7 — Source data Fig. 4 [file 44319_2024_250_MOESM7_ESM.zip › EMBOR-2024-59387_SourceDataForFigure4/EMBOR-2024-59387_SourceDataForFigure4C/Replica-1/Western-SUMO1-PP5-IP-REP1.tif]

# 4C-Replicate 2

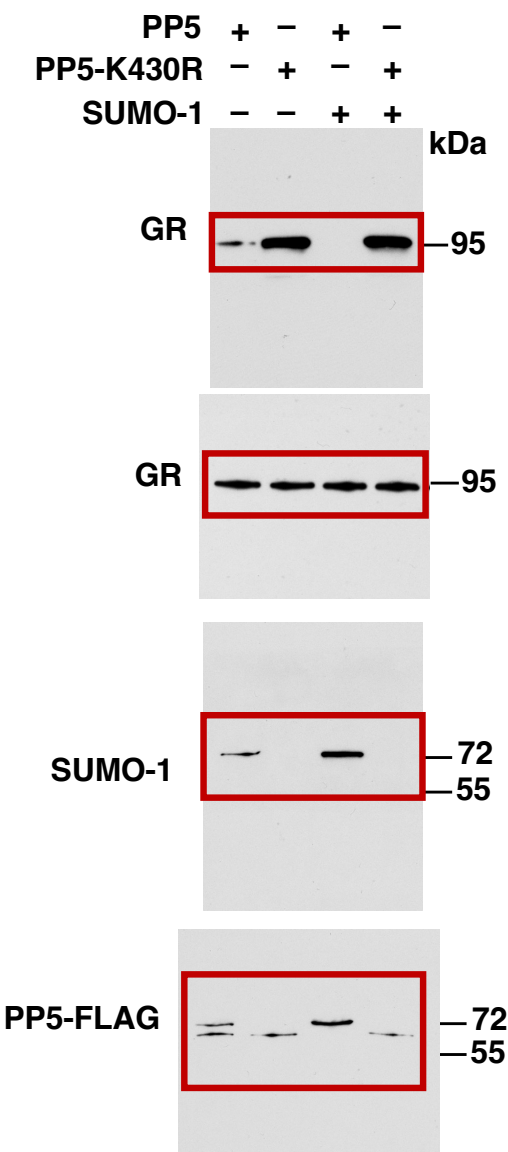

Supplement: Supplementary file 7 — Source data Fig. 4 [file 44319_2024_250_MOESM7_ESM.zip › EMBOR-2024-59387_SourceDataForFigure4/EMBOR-2024-59387_SourceDataForFigure4C/Replica-2/western uncropped annotated.pdf]

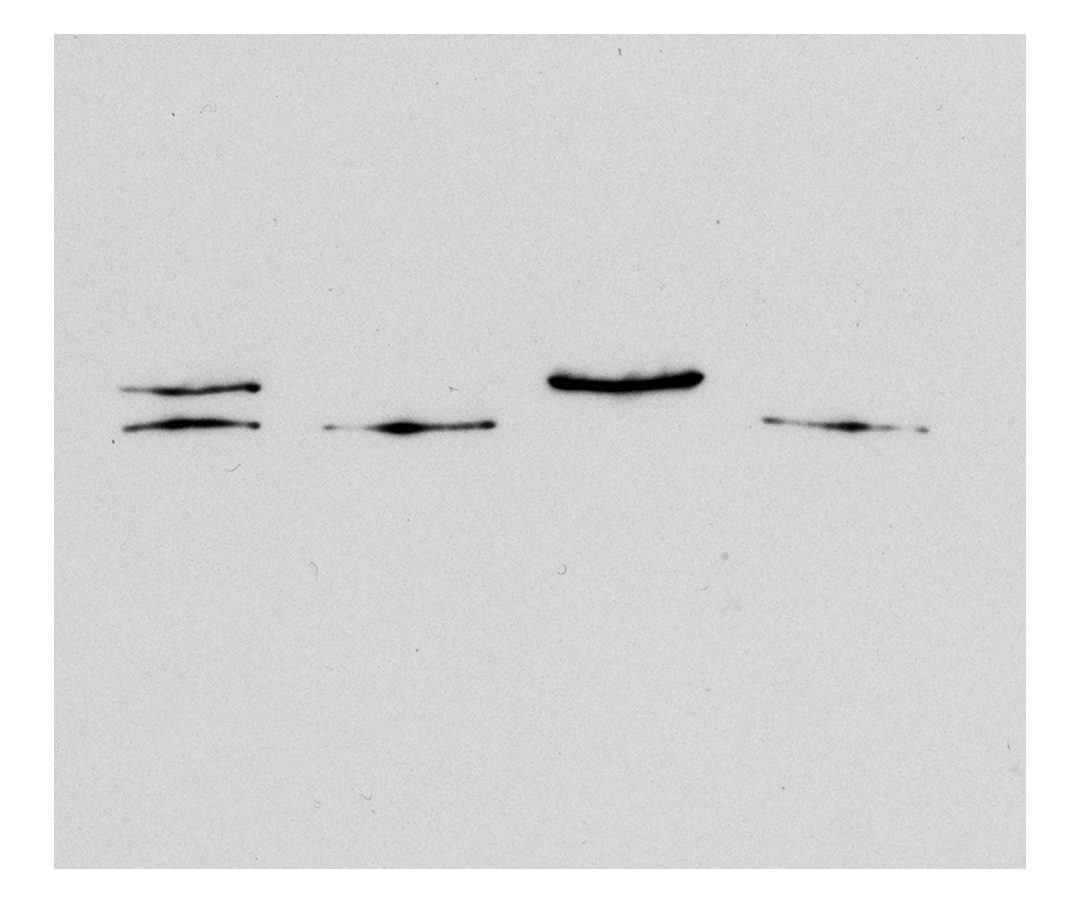

Supplement: Supplementary file 7 — Source data Fig. 4 [file 44319_2024_250_MOESM7_ESM.zip › EMBOR-2024-59387_SourceDataForFigure4/EMBOR-2024-59387_SourceDataForFigure4C/Replica-2/Western-FLAGPP5-IP-REP2.tif]

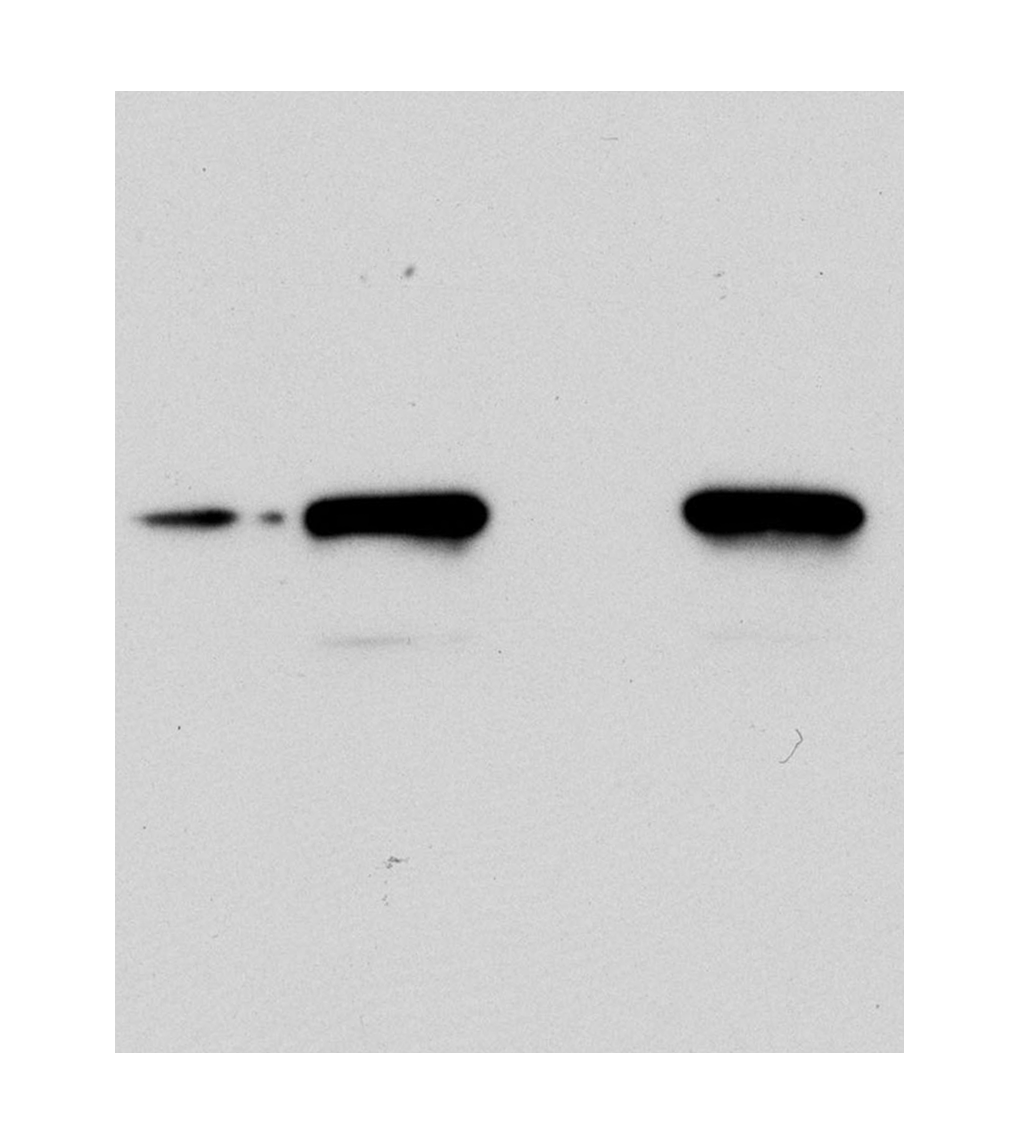

Supplement: Supplementary file 7 — Source data Fig. 4 [file 44319_2024_250_MOESM7_ESM.zip › EMBOR-2024-59387_SourceDataForFigure4/EMBOR-2024-59387_SourceDataForFigure4C/Replica-2/Western-GR-Co-IP-REP2.tif]

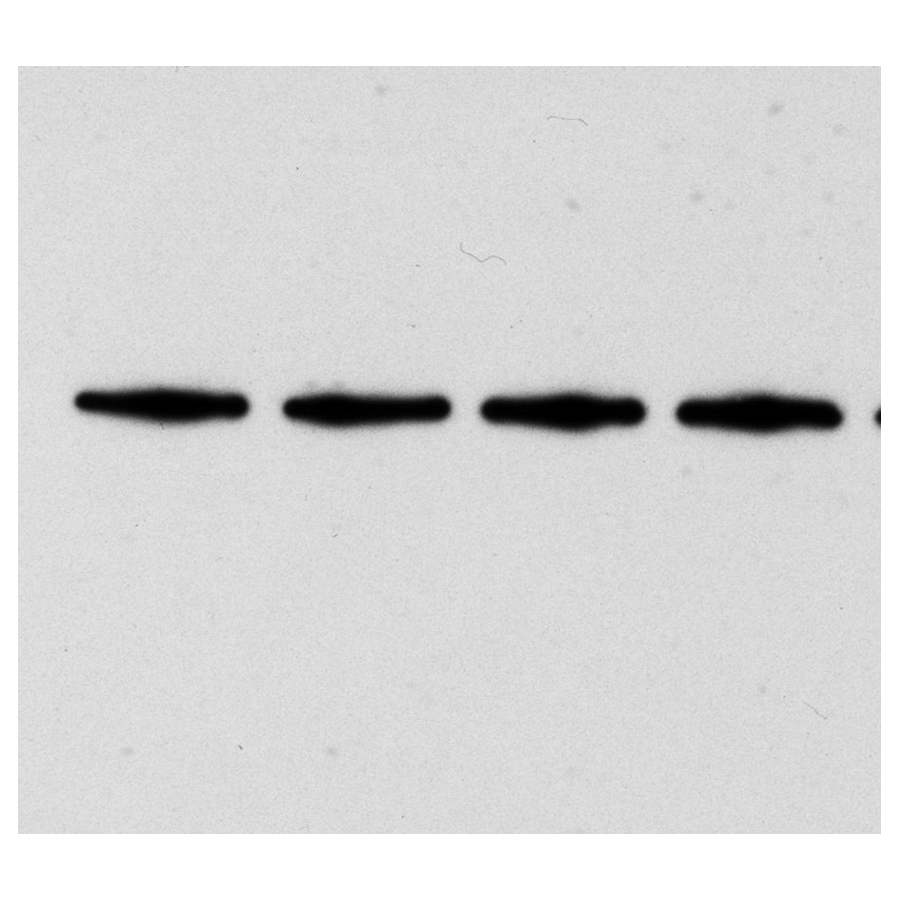

Supplement: Supplementary file 7 — Source data Fig. 4 [file 44319_2024_250_MOESM7_ESM.zip › EMBOR-2024-59387_SourceDataForFigure4/EMBOR-2024-59387_SourceDataForFigure4C/Replica-2/Western-GR-input-REP2.tif]

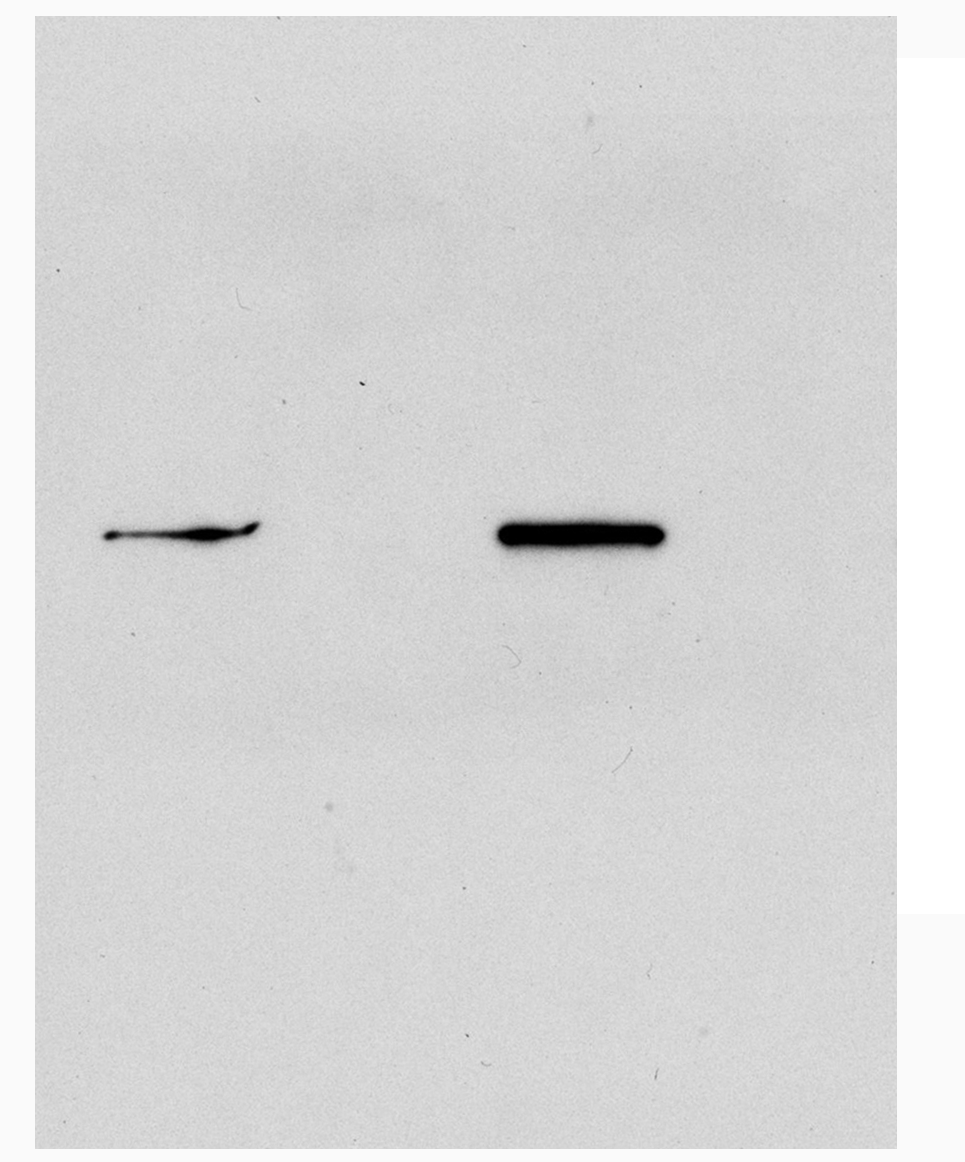

Supplement: Supplementary file 7 — Source data Fig. 4 [file 44319_2024_250_MOESM7_ESM.zip › EMBOR-2024-59387_SourceDataForFigure4/EMBOR-2024-59387_SourceDataForFigure4C/Replica-2/Western-SUMO1-PP5-IP-REP2.tif]

# 4C-Replicate 3

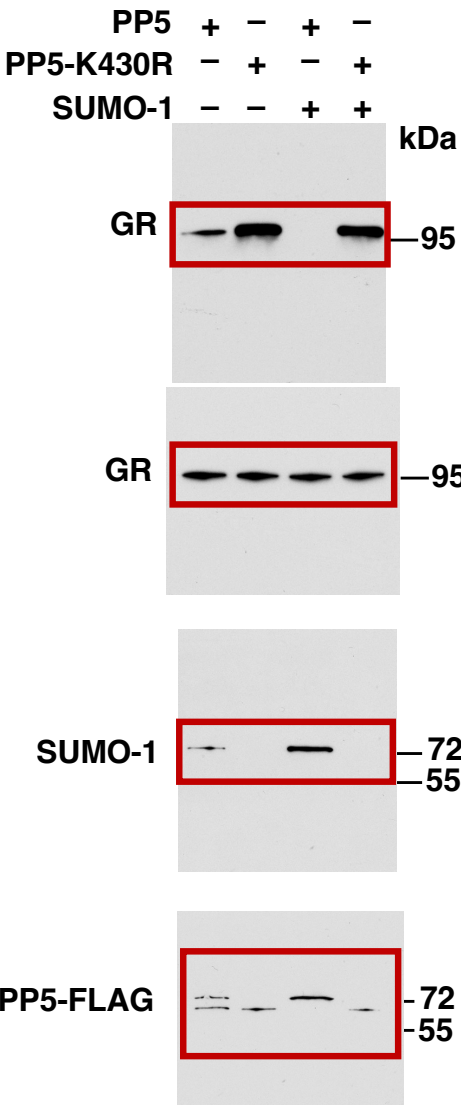

Supplement: Supplementary file 7 — Source data Fig. 4 [file 44319_2024_250_MOESM7_ESM.zip › EMBOR-2024-59387_SourceDataForFigure4/EMBOR-2024-59387_SourceDataForFigure4C/Replica-3/western uncropped annotated.pdf]

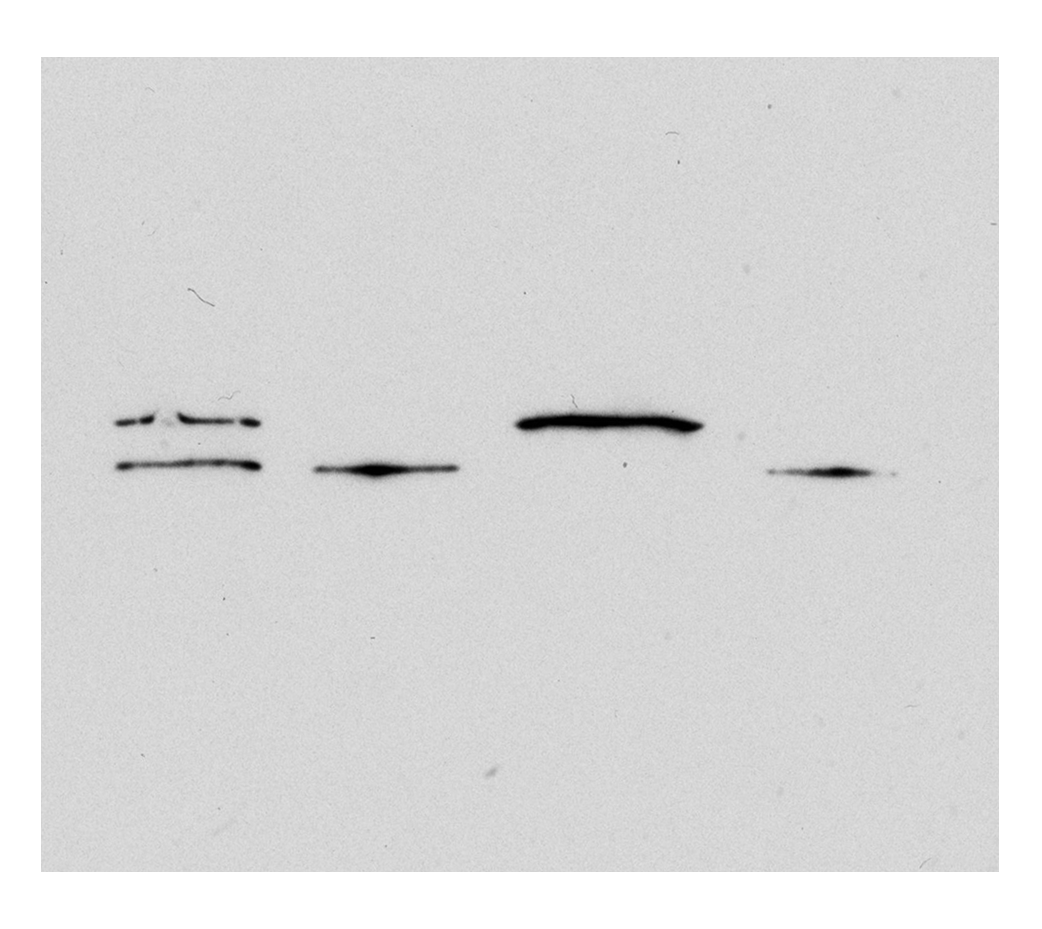

Supplement: Supplementary file 7 — Source data Fig. 4 [file 44319_2024_250_MOESM7_ESM.zip › EMBOR-2024-59387_SourceDataForFigure4/EMBOR-2024-59387_SourceDataForFigure4C/Replica-3/Western-FLAGPP5-IP-REP3.tif]

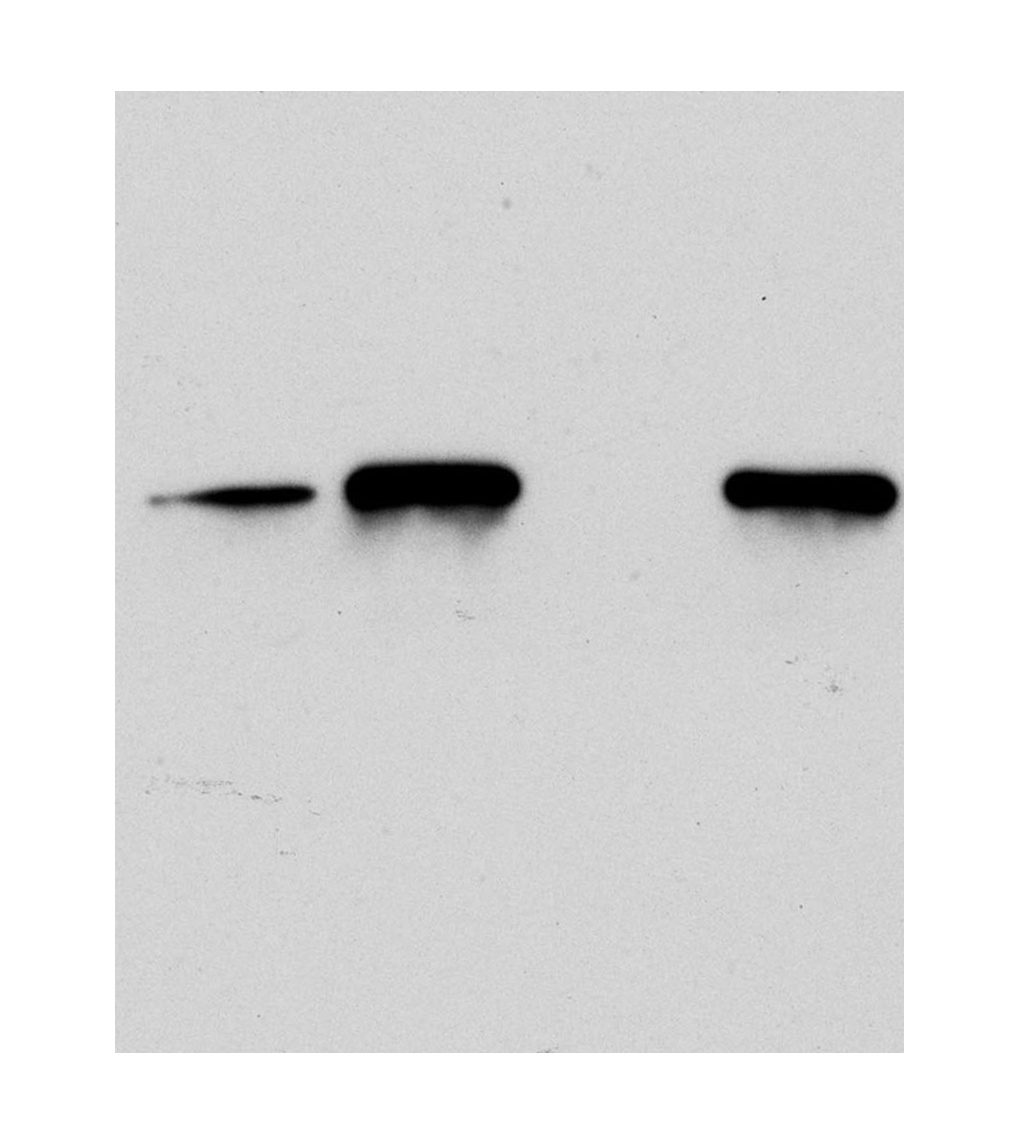

Supplement: Supplementary file 7 — Source data Fig. 4 [file 44319_2024_250_MOESM7_ESM.zip › EMBOR-2024-59387_SourceDataForFigure4/EMBOR-2024-59387_SourceDataForFigure4C/Replica-3/Western-GR-Co-IP-REP3.tif]

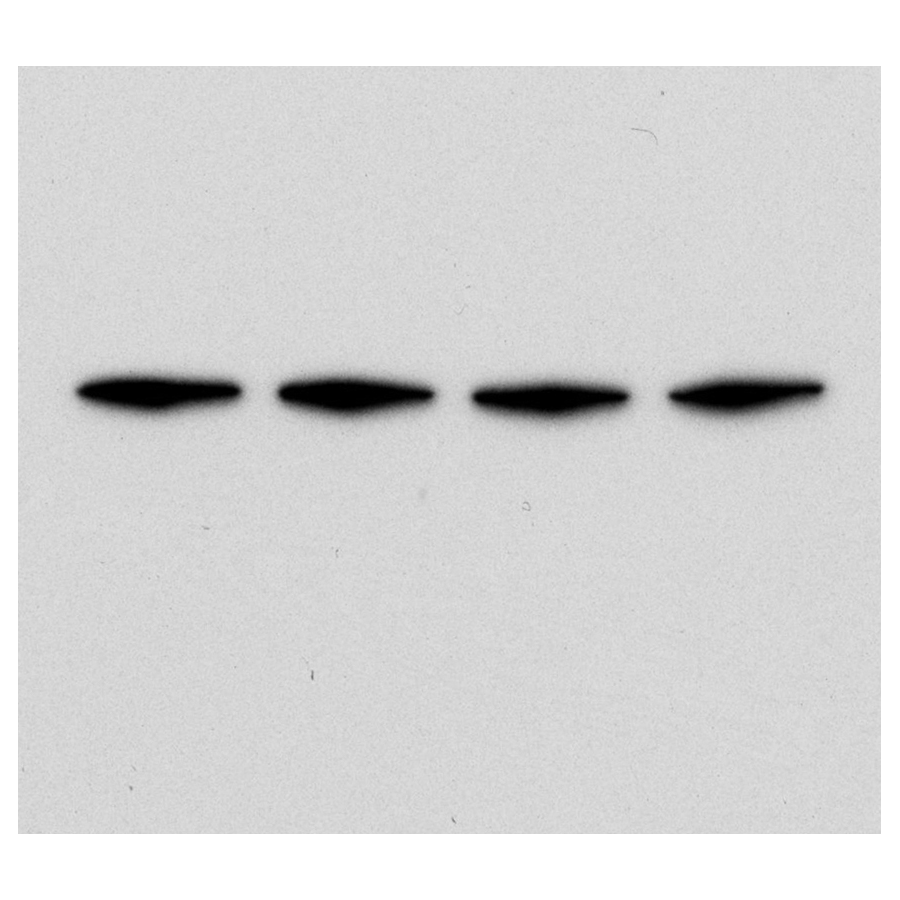

Supplement: Supplementary file 7 — Source data Fig. 4 [file 44319_2024_250_MOESM7_ESM.zip › EMBOR-2024-59387_SourceDataForFigure4/EMBOR-2024-59387_SourceDataForFigure4C/Replica-3/Western-GR-input-REP3.tif]

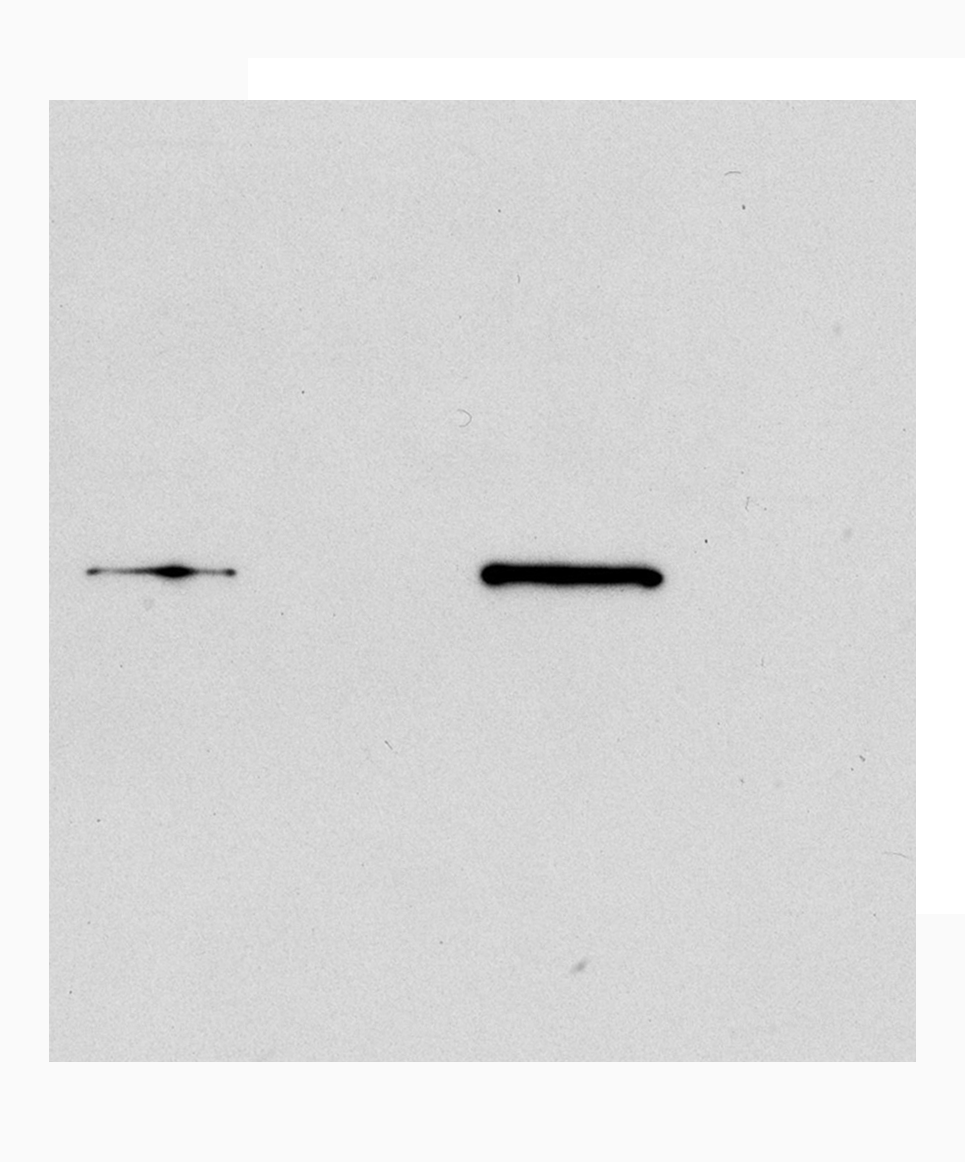

Supplement: Supplementary file 7 — Source data Fig. 4 [file 44319_2024_250_MOESM7_ESM.zip › EMBOR-2024-59387_SourceDataForFigure4/EMBOR-2024-59387_SourceDataForFigure4C/Replica-3/Western-SUMO1-PP5-IP-REP3.tif]

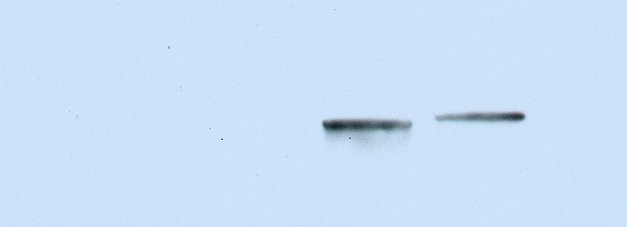

Supplement: Supplementary file 8 — Source data Fig. 5 [file 44319_2024_250_MOESM8_ESM.zip › EMBOR-2024-59387_SourceDataForFigure5/EMBOR-2024-59387_SourceDataForFigure5D/western FLAG.jpg]

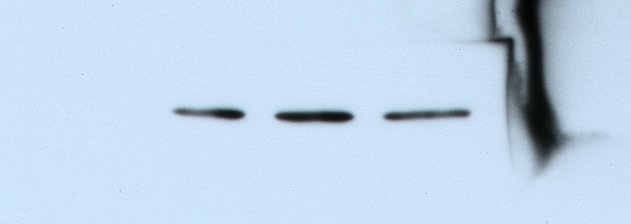

Supplement: Supplementary file 8 — Source data Fig. 5 [file 44319_2024_250_MOESM8_ESM.zip › EMBOR-2024-59387_SourceDataForFigure5/EMBOR-2024-59387_SourceDataForFigure5D/western GAPDH.jpg]

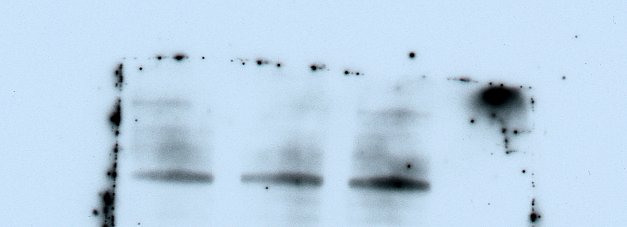

Supplement: Supplementary file 8 — Source data Fig. 5 [file 44319_2024_250_MOESM8_ESM.zip › EMBOR-2024-59387_SourceDataForFigure5/EMBOR-2024-59387_SourceDataForFigure5D/western pTOP2A.jpg]

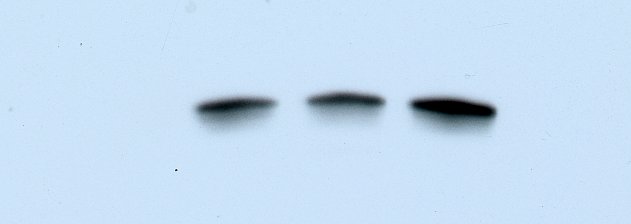

Supplement: Supplementary file 8 — Source data Fig. 5 [file 44319_2024_250_MOESM8_ESM.zip › EMBOR-2024-59387_SourceDataForFigure5/EMBOR-2024-59387_SourceDataForFigure5D/western TOP2A.jpg]

5D

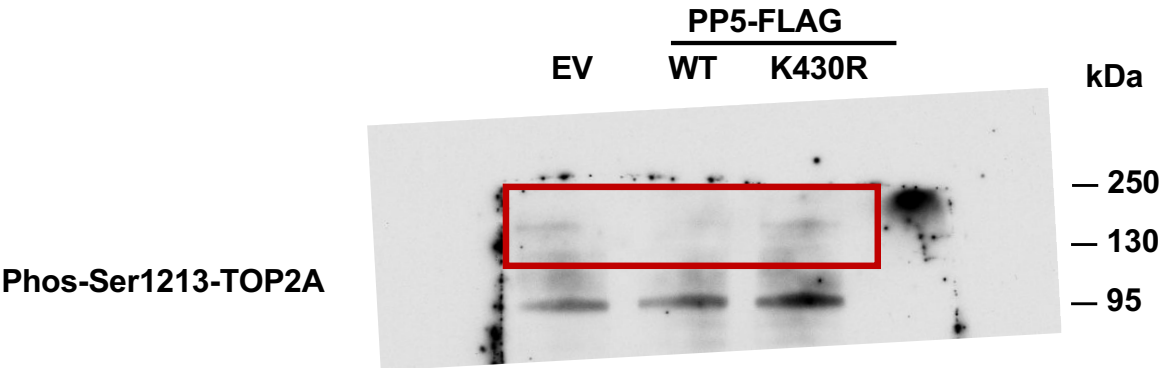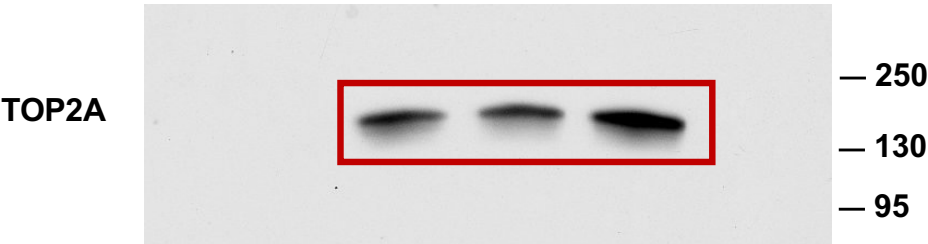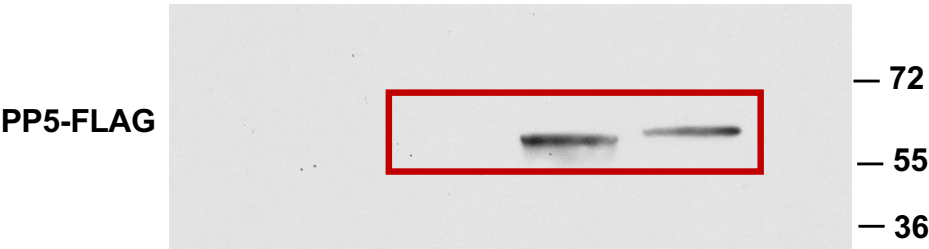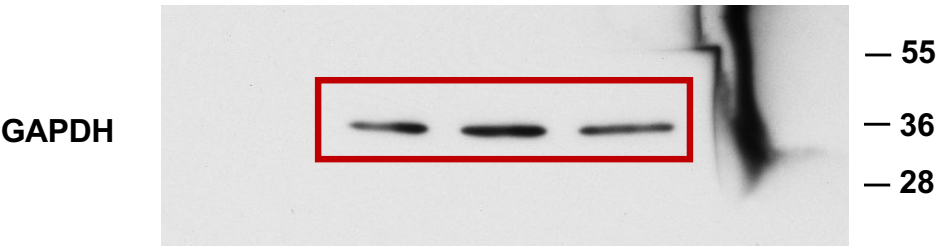

Supplement: Supplementary file 8 — Source data Fig. 5 [file 44319_2024_250_MOESM8_ESM.zip › EMBOR-2024-59387_SourceDataForFigure5/EMBOR-2024-59387_SourceDataForFigure5D/western uncropped annotated.pdf]
