# Supplementary material for: SUMOylation of protein phosphatase 5 regulates phosphatase activity and substrate release
Source: EMBO Rep. 2024 Sep 20;25(11):4. doi: 10.1038/s44319-024-00250-2 (PMC11549447; doi:10.1038/s44319-024-00250-2)
Supplement: Supplementary file 9 — Expanded View Figures [file 44319_2024_250_MOESM9_ESM.pdf]

Expanded View Figures

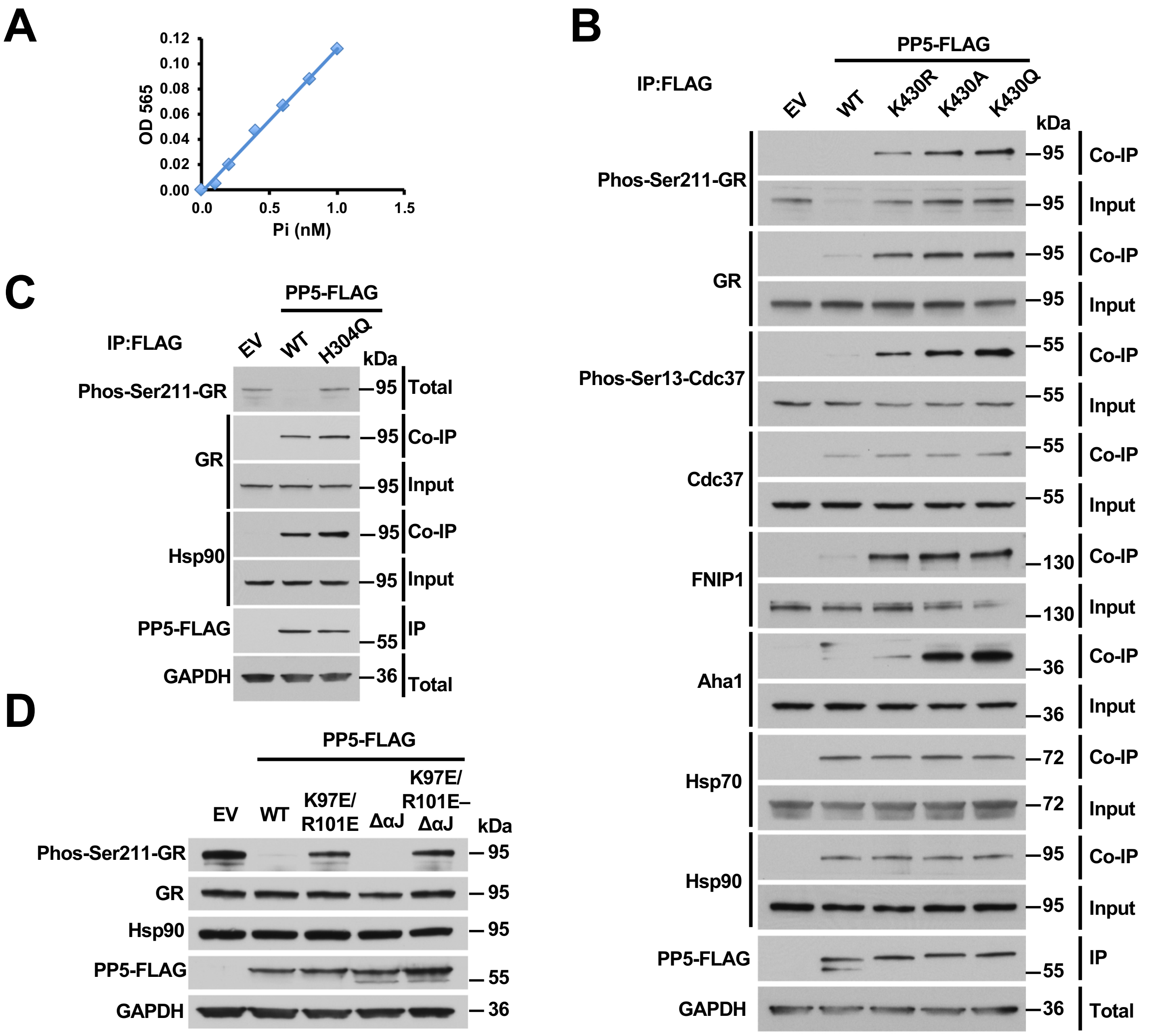

Figure EV1. Role of PP5 SUMOylation toward phosphatase activity.

(**A**) Phosphate standard curve for in vitro PP5 activity measured using PiPer phosphate assay in Fig. 2A. (**B**) PP5-FLAG WT, K430R, K430A, and K430Q were transiently transfected and IP. Co-IP of chaperones, co-chaperones, and known PP5 substrates were analyzed by immunoblotting. EV was used as a control. (**C**) Wild-type PP5-FLAG and H304Q mutant were expressed in an IP. Activity and binding were analyzed by Western blot. EV was used as a control. (**D**) PP5-FLAG WT, K97E/R101E, ΔαJ, and the double mutant PP5-K97E/R101E-ΔαJ were transiently expressed. The activity of PP5, as assessed by dephosphorylation of phospho-GR-S211 was assessed by immunoblotting.

This is a U.S. Government work and not under copyright protection in the US; foreign copyright protection may apply

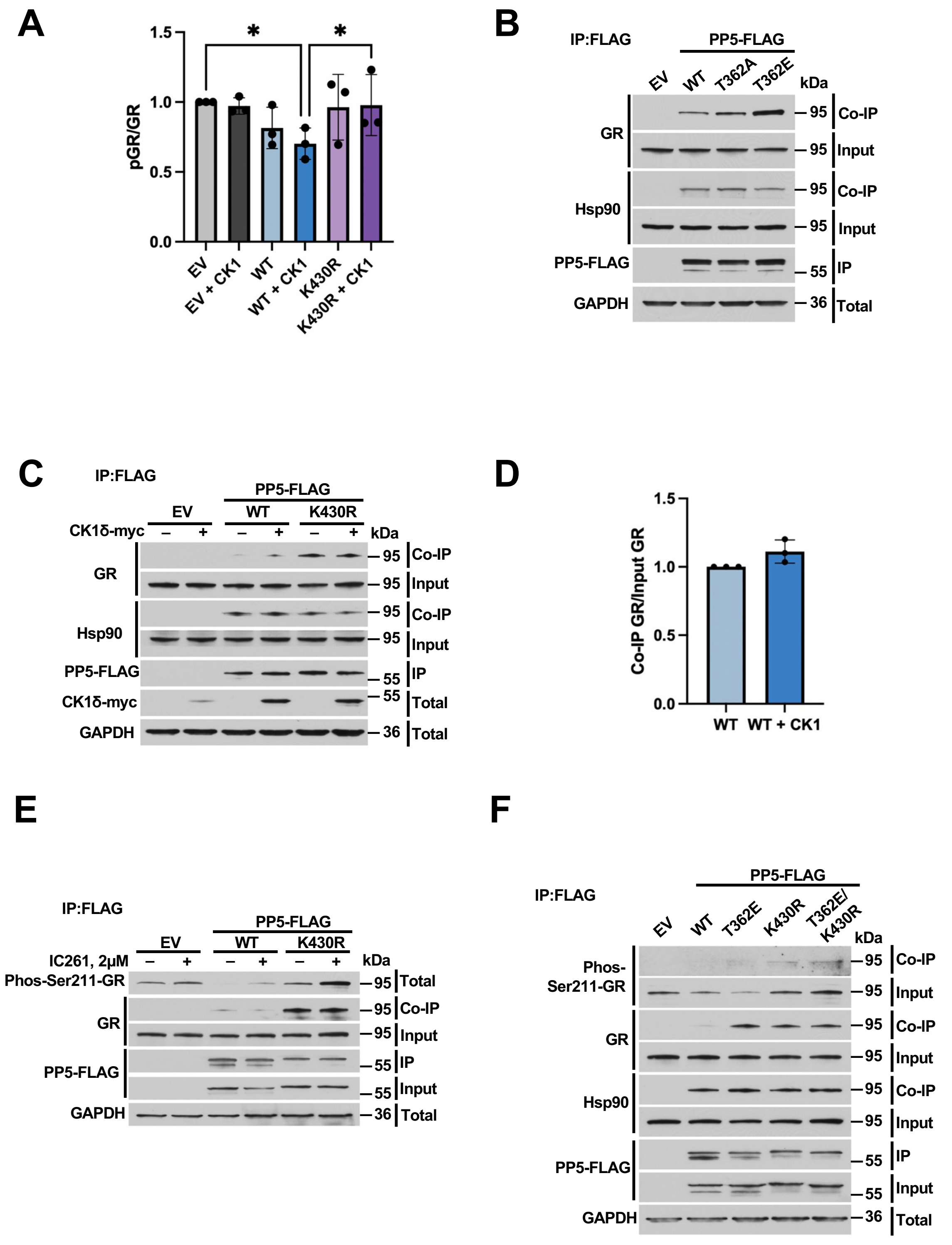

This is a U.S. Government work and not under copyright protection in the US; foreign copyright protection may apply

Figure EV2. Cross-talk of PP5 phosphorylation and SUMOylation.

(**A**) Densitometric analysis of GR-S211 phosphorylation normalized to total GR protein levels from Fig. 3F. Ratios were normalized to EV. Ordinary one-way ANOVA was used to determine statistical significance between samples from three biological replicates ($n = 3$). Data presented show mean ± standard deviation. EV vs. PP5-WT + CK1δ $p = 0.0344$, PP5-WT + CK1δ vs. PP5-K430R + CK1δ $p = 0.0471$. (**B**) Wild-type PP5-FLAG, PP5-T362A, and PP5-T362E were IP, and the binding of the substrate GR and chaperone Hsp90 were assessed by immunoblotting. (**C**) PP5-FLAG WT or K430R were co-transfected with EV or CK1δ-myc. Following IP of PP5-FLAG, co-IP of GR, and Hsp90 were assessed by Western blot. EV was used as a control. Samples and representative input blots for GR, Hsp90, CK1δ-myc, and GAPDH are the same as seen in Fig. 3F. (**D**) Densitometric analysis of GR Co-IP normalized to total GR protein levels from Fig. EV2C. Ratios were normalized to WT. Data were presented as mean ± standard deviation derived from three biologiclal replicates ($n = 3$). (**E**) HEK293 cells were transfected with EV, PP5-FLAG WT, or PP5-K430R. Cells were then treated with vehicle or 2 μM IC261 (CK1δ inhibitor) for 16 h. PP5 activity and binding to the substrate GR were assessed by immunoblotting. (**F**) PP5-FLAG WT, T362E, PP5-K430R, and the PP5-T362E-K430R double mutant were IP. The binding of PP5-FLAG mutants to the chaperone Hsp90 and substrate GR were assessed by immunoblotting. EV was used as a control. Samples and representative input blots for phos-S211-GR, GR, Hsp90, PP5-FLAG, and GAPDH are the same as seen in Fig. 3E.

This is a U.S. Government work and not under copyright protection in the US; foreign copyright protection may apply

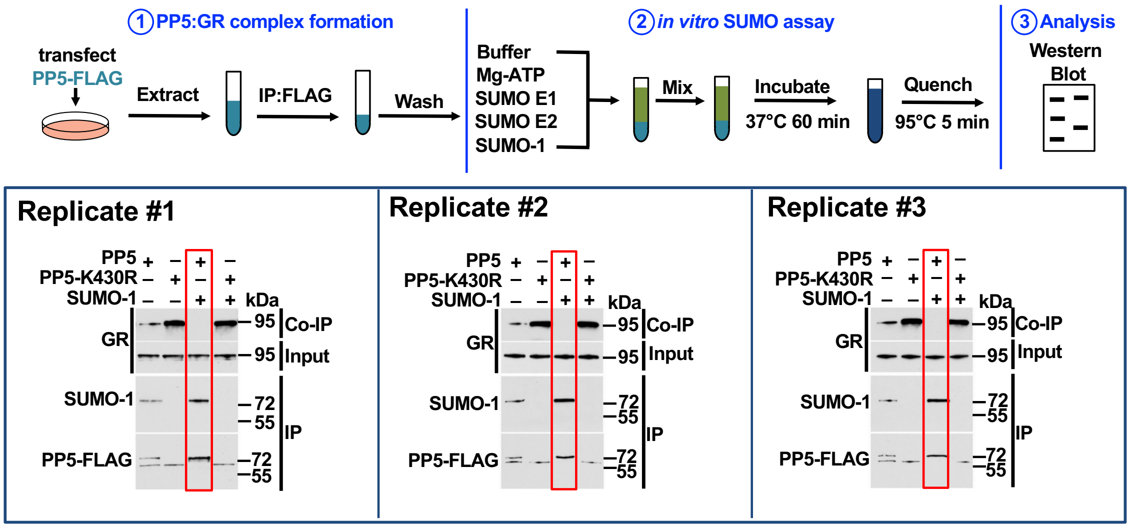

Figure EV3. PP5 in vitro SUMOylation and substrate release.

PP5-FLAG WT or K430R were expressed and isolated for use in an in vitro SUMOylation assay. PP5-WT and K430R were incubated with ATP in the presence and absence of recombinant SUMO-1. FLAG IP was used to isolate PP5 following in vitro SUMOylation and interaction with GR was evaluated by immunoblot. PP5 SUMOylation was assessed by immunoblotting. The data shown are three biological replicates. Replicate three is shown in Fig. 4C.

This is a U.S. Government work and not under copyright protection in the US; foreign copyright protection may apply

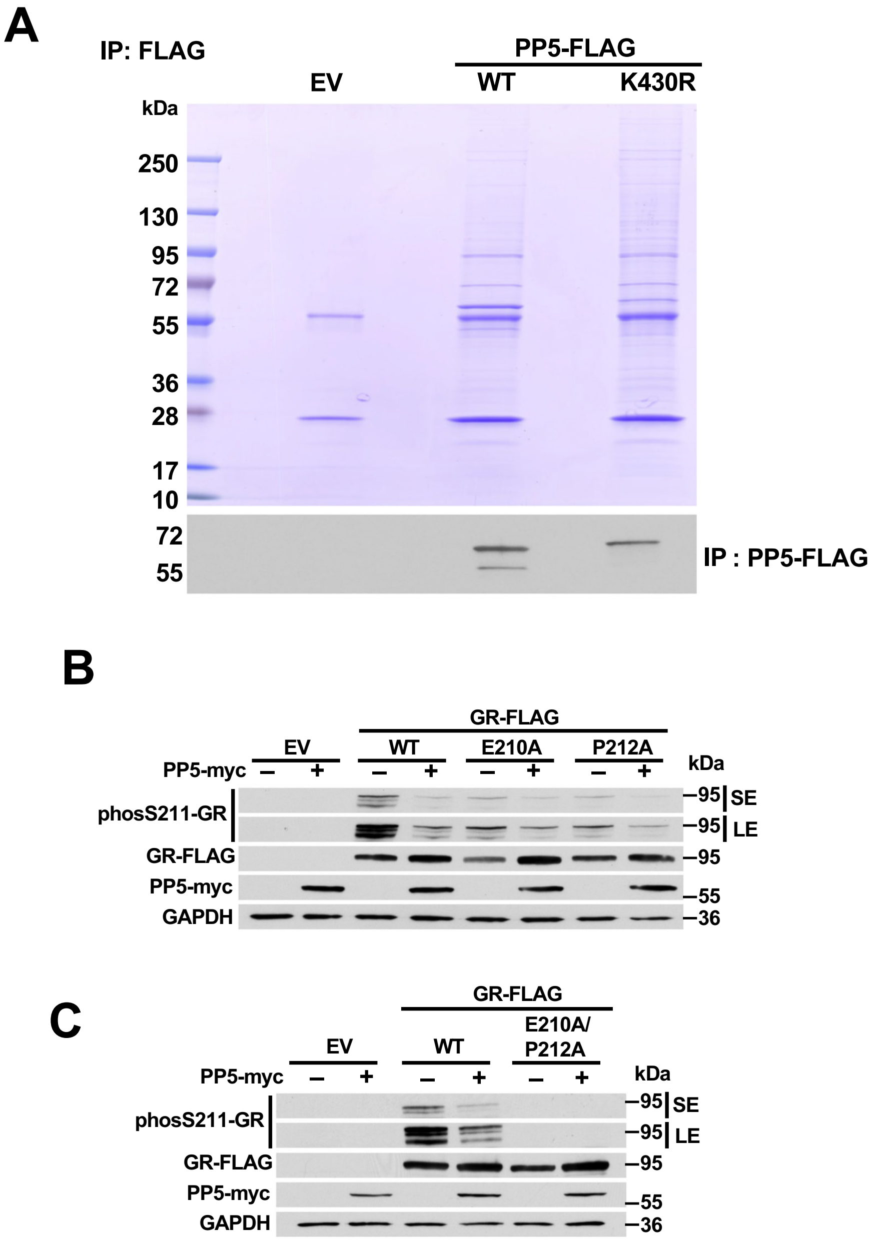

Figure EV4. Generation and analysis of interactome of PP5-K430R.

(**A**) PP5-FLAG WT and K430R were IP from HEK293 cell lysate and subject to mass spectrometry analysis to identify interacting proteins in Fig. 5A. A small aliquot of each sample was also run on SDS-PAGE for examination by Coomassie staining (above) and Western blot for PP5-FLAG (below). EV was used as a control. (**B**) GR-FLAG WT, GR-E210A, or GR-P212A were transiently transfected with or without co-transfection of PP5-myc. The activity of PP5-FLAG towards dephosphorylation of total phospho-GR-S211 was assessed by immunoblotting. EV was used as a control. (**C**) GR-FLAG WT or GR-E210A/P212A double mutants were transiently transfected with or without co-transfection of PP5-myc. Activity of PP5-FLAG towards dephosphorylation of phospho-GR-S211 was assessed by immunoblotting. EV was used as a control.

This is a U.S. Government work and not under copyright protection in the US; foreign copyright protection may apply
